# Tetraaza[7]–[15]helicenes Synthesized by Two‐Step Strategy: Length‐Controlled Chiral π‐Systems Exhibiting Amplified Circularly Polarized Luminescence

**DOI:** 10.1002/anie.202524463

**Published:** 2026-01-09

**Authors:** Takashi Otani, Yuchen Wu, Kohei Ueda, Yuki Ikeda, Yuna Tada, Natsuna Kinoshita, Takanori Shibata

**Affiliations:** ^1^ Course of Chemical Engineering National Institute of Technology Anan College 265 Aoki, Minobayashi Anan Tokushima 774‐0017 Japan; ^2^ School of Advanced Science and Engineering Waseda University 3‐4‐1 Okubo Shinjuku Tokyo 169‐8555 Japan

**Keywords:** Azahelicenes, Chiroptical properties, Circularly polarized luminescence, Helicenes, π‐Conjugated materials

## Abstract

Helicenes are chiral π‐conjugated molecules whose properties strongly depend on their lengths. Systematic studies of these compounds have been limited by synthetic challenges. Here we report a concise two‐step strategy (defined as the helicene‐forming sequence from aminohelicene precursors) to access a homologous series of tetraaza[7]–[15]helicenes. Optical spectra converge beyond [11]H, defining a conjugation ceiling, while chiroptical responses amplify sharply, yielding |*g*_lum_| up to 0.028. Fluorescence quantum yields show a nonmonotonic dependence, with [7]H and [15]H maintaining both high *Φ*_F_ (0.39 and 0.36) and large |*g*_lum_|, resulting in outstanding, albeit semi‐quantitative, CPL performance, with figures of merit reaching 0.010 and CPL brightness values of approximately 490. TD‐DFT calculations attribute this amplification to the delayed alignment of electric and magnetic transition dipoles, while ^1^H NMR shifts of inner protons provide an independent probe of structural reorganization within the helical cavity. Additionally, experiment and theory have consistently identified [11]H as the critical helicene length at which the framework undergoes a qualitative transition. Notably, the [15]helicene constitutes the longest helicene ever resolved into its enantiomers, underscoring the synthetic power of this modular approach. Importantly, our synthetic route is effective for constructing higher‐order helicenes, offering a generalizable platform for length‐controlled, heteroatom‐containing helicenes. These findings establish long tetraazahelicenes as a rare platform where through‐bond conjugation and through‐space orbital coupling act cooperatively to govern photophysical and chiroptical properties.

## Graphical abstract

A modular two‐step strategy provides tetraaza[*n*]helicenes (*n* = 7–15) as a homologous series with tunable photophysical and chiroptical properties. Extension beyond *n* = 11 induces pronounced through‐space π–π interactions, narrowing the energy gap and amplifying circularly polarized luminescence.

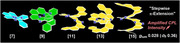

## Introduction

**Figure 1:**
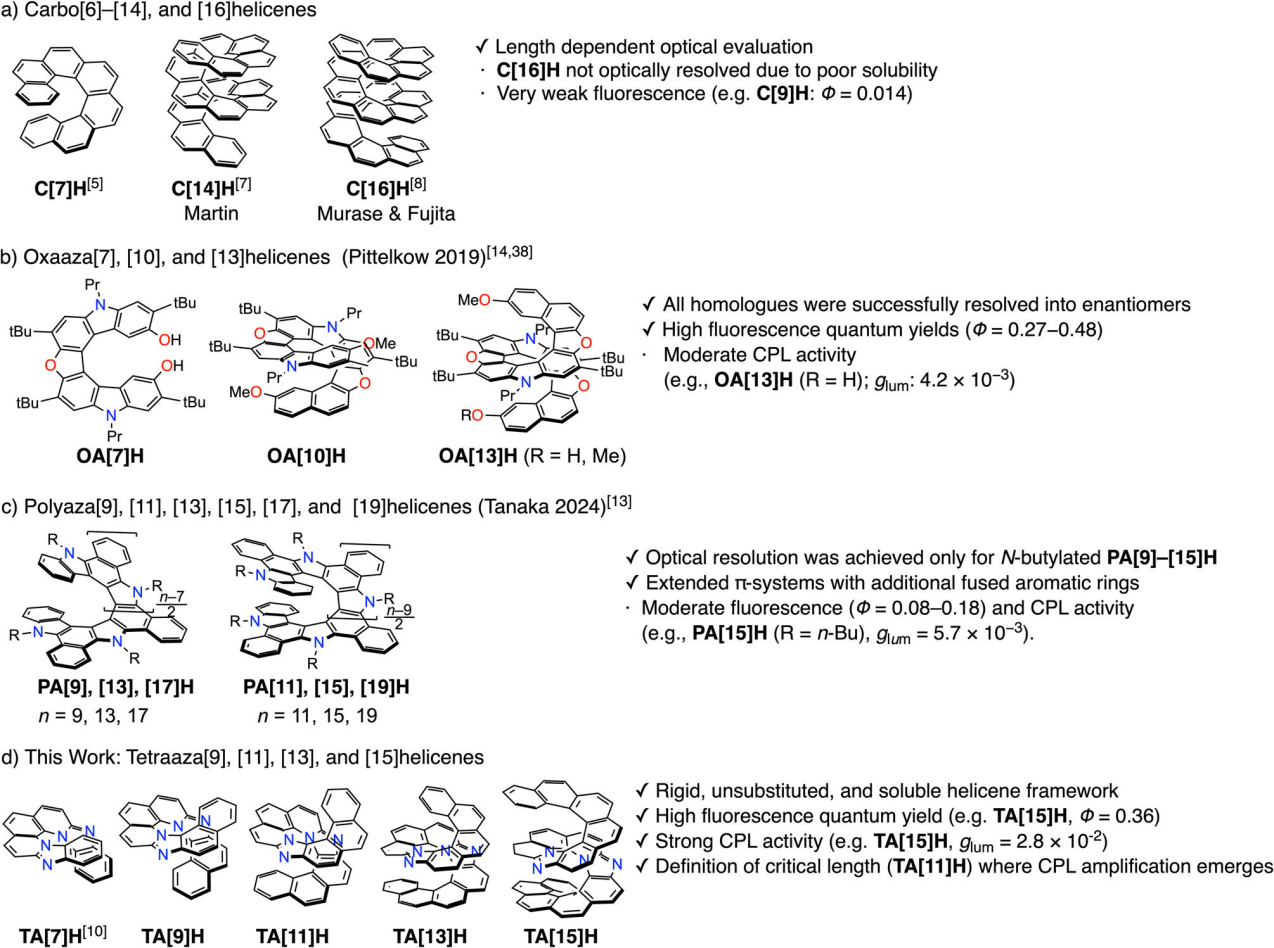
a)–c) All helicenes with 13 or more rings reported to date; d) the newly synthesized tetraazahelicenes presented in this study.

Helicenes are chiral π‐conjugated molecules composed of ortho‐fused aromatic rings that adopt a helical arrangement.^[^ ^1^, ^2^, ^3^, ^4^ ^]^ Their screw‐shaped geometry leads to chirality in the absence of stereogenic centers, particularly when the number of fused rings (*n* in [*n*]helicene) exceeds five. While classical carbohelicenes consist solely of benzene rings,^[^ ^5^, ^6^, ^7^, ^8^ ^]^ heterohelicenes containing heteroatom(s), such as nitrogen and oxygen atoms, have attracted increasing attention due to their tunable electronic and optical properties.^[^ ^9^, ^10^, ^11^, ^12^, ^13^, ^14^ ^]^ Helicenes have long fascinated both experimental and theoretical scientists, and studies have examined electronic structures and ionization potentials,^[^ ^15^, ^16^ ^]^ mechanical elasticity,^[^ ^17^, ^18^ ^]^ nonlinear optical properties,^[^ ^19^, ^20^ ^]^ racemization dynamics,^[^ ^21^, ^22^ ^]^ and optical resolution.^[^ ^23^, ^24^ ^]^ More recently, their use as circularly polarized light emitters has motivated intensive research on their circular dichroism (CD)^[^ ^25^, ^26^, ^27^ ^]^ and circularly polarized luminescence (CPL).^[^ ^28^, ^29^, ^30^, ^31^ ^]^ The effect of helicene length on optical and chiroptical properties has been a particular focus of theoretical investigations.^[^ ^15^, ^32^ ^]^ Such studies ideally require a homologous series of helicenes differing only in the number of fused rings, but synthetic access has been limited. Classical methods include photooxidative cyclization,^[^ ^7^, ^8^ ^]^ [2+2+2] cycloaddition,^[^ ^33^, ^34^, ^35^ ^]^ and Friedel–Crafts annulations,^[^ ^36^ ^]^ while oxidative C─H/N─H couplings with hypervalent iodine reagents^[^ ^10^, ^11^, ^12^, ^13^ ^]^ have broadened synthetic options. Nevertheless, the preparation of higher‐order helicenes—especially triple‐layer structures with more than 13 rings—remains challenging (Figure 1).^[^ ^7^, ^8^, ^13^, ^14^ ^]^

The longest fully benzene‐annulated helicene synthesized to date is [16]helicene (**C[16]H**) reported by Fujita, which exhibits poor solubility and is non‐emissive (Figure 1a).^[^ ^8^ ^]^ Even for the shorter homologue **C[9]H**, the fluorescence quantum yield is as low as 0.014.^[^ ^37^ ^]^ Pittelkow and co‐workers subsequently developed an iterative oxidative annulation strategy to access oxaazahelicenes up to [13]H (**OA[13]H**), including symmetric, unsymmetrical, and asymmetric variants, and successfully achieved optical resolution of **OA[13]H** enantiomers, which at the time represented the longest optically pure helicenes reported (Figure 1b).^[^ ^14^ ^]^ **OA[13]H** is highly fluorescent, but its circularly polarized luminescence (CPL) activity is moderate (*g*_lum_ = 4.2 × 10^−3^).^[^ ^38^ ^]^ More recently, Tanaka and co‐workers synthesized polyazahelicenes up to [19]H (**PA[19]H**), featuring multiple additional fused aromatic rings and fully conjugated triple‐layered structures (Figure 1c).^[^ ^13^ ^]^ Although this series extended the record helicene length, optical resolution was achieved only for *N*‐butylated derivatives up to [15]H (**PA[15]H**), leaving the longest members unresolved. **PA[*n*]Hs** are also fluorescent, their CPL activities are also moderate (e.g., **PA[15]H** (R = *n* Bu) *g*_lum_ = 5.7 × 10^−3^).

Against this background, we set out to develop an original series of polyazahelicenes that are rigid, soluble, length‐defined, and free from peripheral substituents, enabling meaningful structure–property correlations (Figure 1d). We previously established a concise two‐step strategy to tetraaza[7]helicene (**TA[7]H**),^[^ ^10^, ^11^, ^12^ ^]^ affording a soluble, fluorescent, and CPL‐active framework. Building on this strategy, we now report tetraaza[9]–[15]helicenes and present a combined experimental and theoretical investigation of their photophysical and chiroptical properties. While the present work builds on our earlier approach to tetraaza[7]helicenes,^[^ ^10^ ^]^ its conceptual advance lies in demonstrating that this concise platform can be systematically extended to much longer homologues, thereby enabling meaningful length‐dependent structure–property correlations.

Here, the term “two‐step strategy” denotes the helicene‐forming sequence from aminohelicene precursors, rather than the total number of synthetic operations starting from commercially available materials. As described below, this concise sequence enables efficient construction of tetraaza[*n*]helicenes over a wide range of helicene lengths.

Importantly, in contrast to the polyazahelicenes (**PA[*n*]Hs**), which possess additional fused benzene rings beyond the helical skeleton, our tetraazahelicenes retain a structurally pure helicene framework without extra annulation. This simplicity not only enhances solubility but also allows intrinsic structure–property relationships to be clearly defined. Moreover, both fluorescence quantum yields and CPL dissymmetry factors (*g*_lum_) of our tetraazahelicenes significantly surpass those of the polyaza series, demonstrating their superior performance as chiral π‐conjugated emitters. Remarkably, the homologous series reveals a critical length at **TA[11]H**, where the optical spectra converge while the CPL activity is dramatically amplified, highlighting a pronounced length‐dependent enhancement of CPL performance.

## Results and Discussion

### Synthesis

**Scheme 1:**
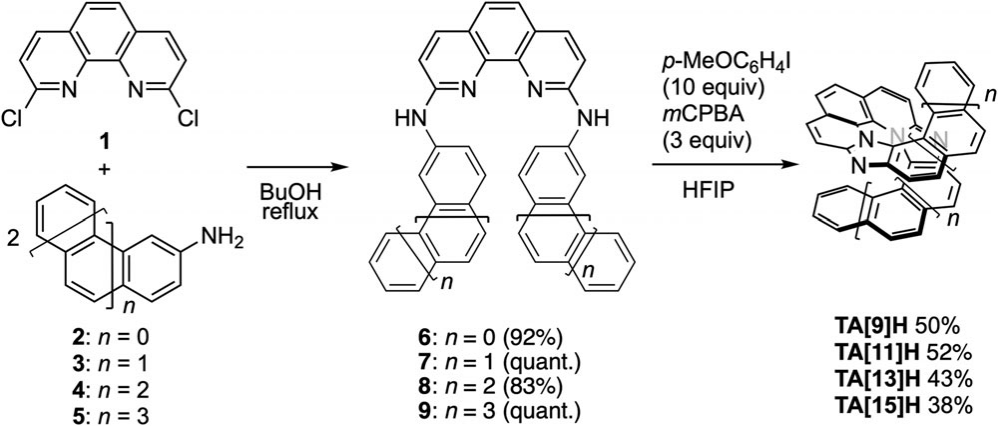
Modular two‐step helicene‐forming strategy for the construction of **TA[9]–[15]H**. [Correction added on January 30, 2026, after first online publication: Scheme 1 and Supporting Information has been updated.]

**Figure 2:**
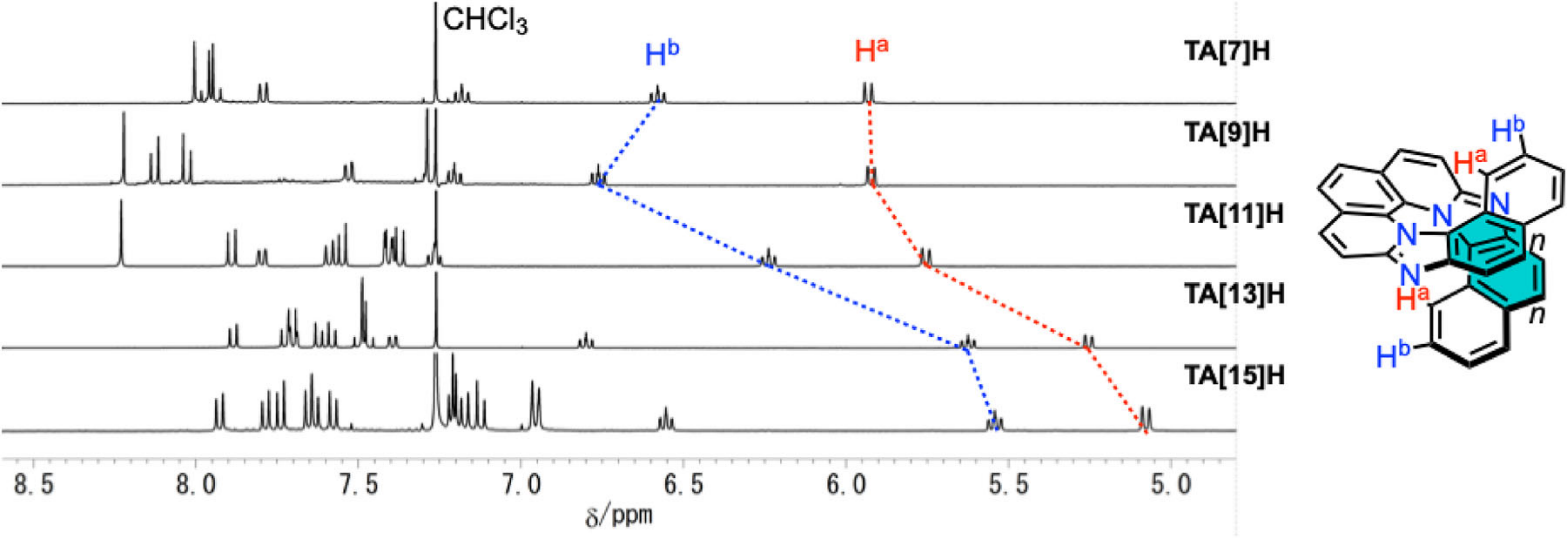
Aromatic region of ^1^H NMR spectra of **TA[7]–[15]H**.

Scheme 1 outlines the modular two‐step strategy of **TA[9]–[15]H**, obtained in 36–52% total yield. In line with our previous report,^[^ ^10^ ^]^ nucleophilic substitution of 2,9‐dichloro‐1,10‐phenanthroline (**1**) with helicene amines **2–5** in boiling *n*‐butanol afforded precursors **6–9**. Subsequent oxidative C─H/N─H coupling was performed in hexafluoro‐2‐propanol (HFIP) with excess amounts of 4‐iodoanisole, while three equivalents of *m*CPBA were added in six portions, giving the corresponding tetraazahelicenes **TA[9]–[15]H** differing only in the number of rings. The preferential formation of helicenes in these cyclizations can be rationalized by the fact that ortho‐fusion maximizes aromatic stabilization and π‐conjugation in the final products, reminiscent of the α‐position selectivity observed in naphthalene electrophilic substitution. In helicene synthesis, however, the selectivity is governed primarily by product stabilization rather than intermediate stability. Importantly, all homologues show good solubility in common organic solvents. Even **TA[15]**H, the least soluble member of the series, dissolves 3 mg in ca. 2 mL of dichloromethane, ca. 10 mL of methanol, and ca. 16 mL of ethyl acetate, thereby overcoming a major barrier to carbohelicenes.^[^ ^8^ ^]^ Although attempts at single‐crystal X‐ray diffraction analyses were hampered mainly by low crystallization propensity of the tetraaza[*n*]helicenes, an X‐ray crystal structure of **TA[11]H** was obtained, confirming the overall helical framework (CCDC 2516246; see Figure S1). The progressive development of the helical cavity is clearly reflected in the ^1^H NMR spectra of **TA[7]–[15]H**, where the innermost (H^a^) and adjacent (H^b^) protons shift systematically upfield and the Δ*δ*(H^a^ – H^b^) decreases with the number of rings (Figure 2). These trends provide a straightforward spectroscopic probe of cavity growth in long azahelicenes. Gauge‐independent atomic orbital (GIAO) calculations reproduce the shifts with high accuracy, supporting the assigned conformations in solution (Figure S9). Notably, in contrast to **TA[7]–[15]H**, the innermost protons of carbo[16]helicene (**C[16]H**) appear downfield,^[^ ^8^ ^]^ underscoring the sensitivity of inner protons to structural compression and multilayer formation in extended helicenes. Finally, to evaluate the generality of the present platform beyond the odd‐numbered series, we explored the feasibility of even‐membered tetraazahelicenes by attempting the synthesis of **TA[10]H,** which was obtained in low yield with limited purity. To assess possible odd–even effects in helicene length, complementary theoretical analyses of even‐membered **TA[*n*]H** were performed; details are provided in the Figure S17.

### Optical Properties

**Figure 3:**
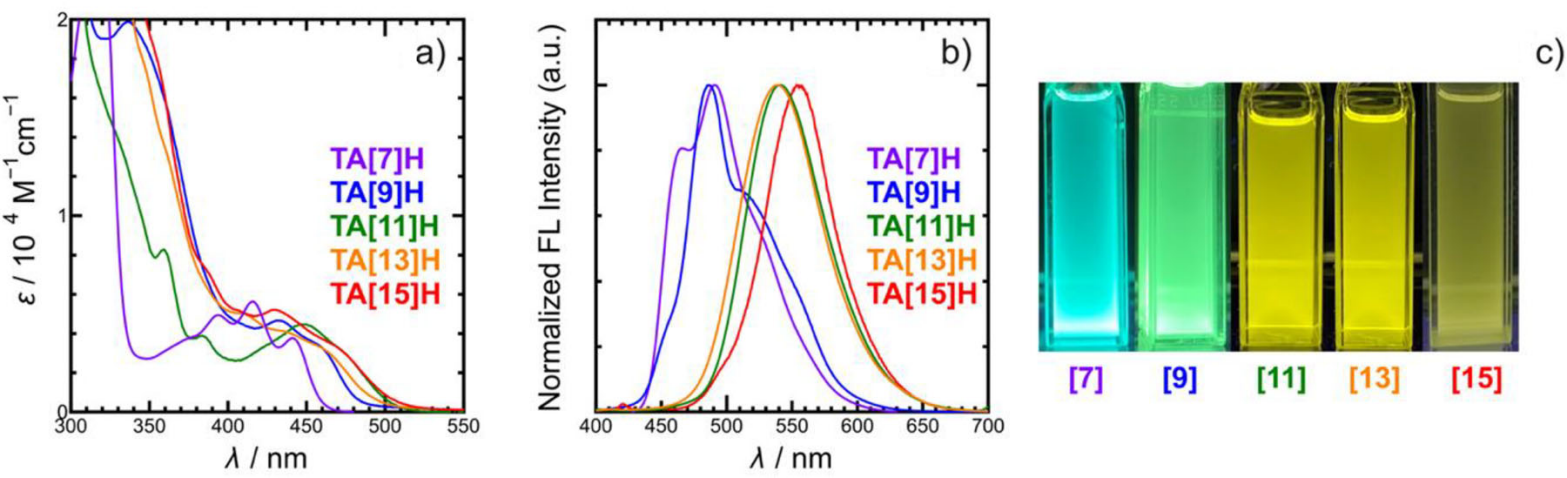
UV–vis absorption spectra a), fluorescence spectra b) and photos under irradiation of UV (*λ* = 365 nm) light c) for **TA[7]–[15]H**.

In the UV–vis spectra of **TA[7]–[15]H** (Figure 3a), intense π–π bands appear at 300–380 nm, while weaker absorptions extend to 380–500 nm and red‐shift systematically with increasing ring number, showing clear vibronic features except for **TA[11]H**. The absorption edges correlate well with the calculated decrease in HOMO–LUMO gaps. Fluorescence spectra (Figure 3b) display strong emission between 450 and 600 nm, with maxima shifting from blue–green (**TA[7],[9]H**) to yellow‐orange (**TA[11]–[15]H**), consistent with the color changes observed under UV light (Figure 3c). The bands remain relatively narrow, consistent with emission from the lowest π–π* state. The quantum yields (*Φ*_F_) vary across the series—0.39 (**TA[7]H**), 0.15 (**TA[9]H**), 0.23 (**TA[11]H**), 0.14 (**TA[13]H**), and 0.36 (**TA[15]H**)—reflecting nonmonotonic changes in nonradiative decay (Table 1). Importantly, all homologues retain strong emission, in contrast to fully benzenoid carbohelicenes of comparable length, which are typically quenched.^[^ ^37^ ^]^ Nitrogen incorporation thus plays a decisive role in maintaining efficient radiative pathways.

**Table 1:** Photophysical properties of polyazahelicenes **TA[7]–[15]H** in dichloromethane.

Comp.	λ_max(abs)_ (nm)	λ_ max(em)_ (nm)^a)^	*Φ*_F_
**TA[7]H** ^[^ ^10^ ^]^	394,416, 441	492	0.39
**TA[9]H**	336, 432, 460 (sh)	486	0.15
**TA[11]H**	359, 384, 448	541	0.23
**TA[13]H**	410 (sh), 436 (sh), 459 (sh)	539	0.14
**TA[15]H**	416 (sh), 488 (sh), 501 (sh)	553	0.36

^a)^ Excitation wavelength: **TA[7]H**: 416 nm; **TA[9]H**: 336 nm; **TA[11]H**: 359 nm; **TA[13]H**: 314 nm; **TA[15]H**: 305 nm.

**Figure 4:**
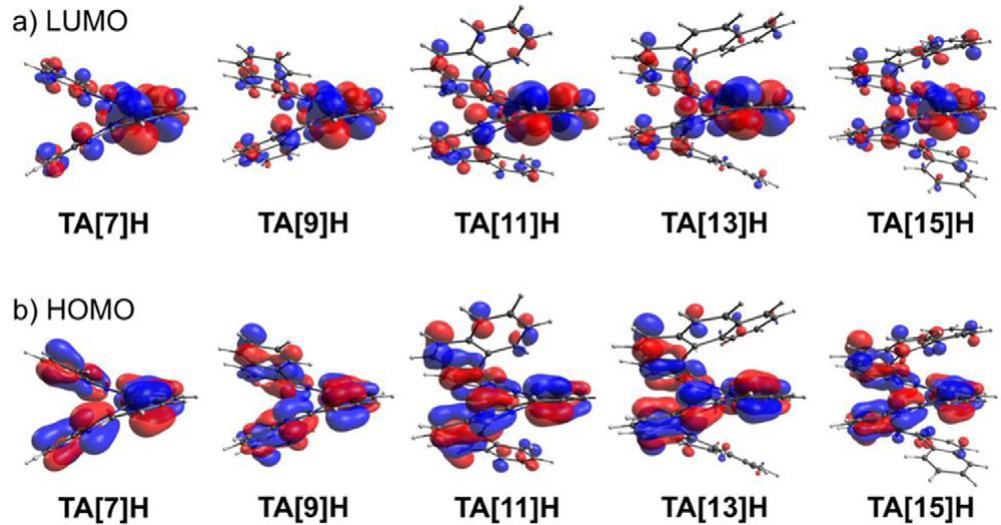
Side views of the a) LUMO and b) HOMO of TA[7]–[15]H, calculated at the PBE0/6–31G(d)/SMD level with an isosurface value set to 0.03.

TD‐DFT simulations reproduce the experimental spectra with remarkable fidelity. As shown in Figure S3, the calculated absorption and CD spectra converge beyond **TA[9]–[11]H**, in agreement with experimental findings. Frontier orbital analysis (Figure 4) reveals that, for **TA[7]–TA[9]H**, both HOMO and LUMO are delocalized along the helical backbone. From **TA[11]H** onward, however, the LUMO becomes increasingly localized on the aza‐phenanthroline core, while the HOMO remains more delocalized. This localization imposes a “conjugation ceiling,” limiting further red‐shifts and resulting in nearly constant absorption maxima for **TA[13]H** and longer.

The HOMO–LUMO gap evolution (Figure S6) highlights **TA[11]H** as a turning point, where the gap narrows abruptly. This correlates with the pronounced red‐shift in both absorption and emission observed experimentally for **TA[11]H**. We attribute this behavior to structural extension beyond *n* = 11, where the helicene subunits expand above and below the aza‐phenanthroline core, promoting pronounced through‐space π–π overlap between non‐bonded rings. This enhanced 3D orbital interaction increases HOMO–LUMO mixing and thereby destabilizes the energy gap. For longer homologues, the absorption and emission maxima converge around **TA[15]H**, indicating saturation of the optical properties within the accessible series.

### Chiroptical Properties

**Figure 5:**
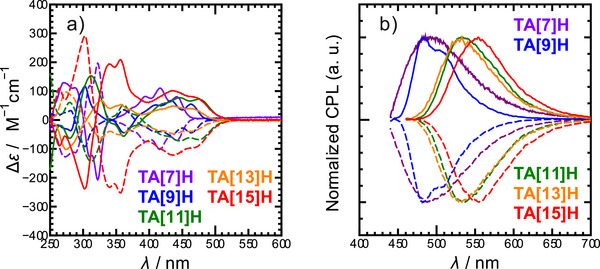
a) Circular dichroism (CD) spectra and b) normalized circularly polarized luminescence (CPL) spectra of TA[7]–[15]H measured in dichloromethane. CPL spectra were recorded at an excitation wavelength of 380 nm and normalized to the maximum emission intensity for clarity.

Racemic samples of **TA[9]–[15]H** were successfully resolved into enantiopure fractions using chiral medium‐pressure liquid chromatography. Notably, **TA[15]H** represents, alongside Tanaka's **PA[15]H** (R = Bu, Figure 1), the longest helicene ever separated into its enantiomers. The corresponding CD spectra (Figure 5a) generally followed the trends observed in the absorption spectra, with the CD intensity extending toward longer wavelengths as the helicene length increased. In the long‐wavelength region above approximately 400 nm, only weak CD signals were observed, which were attributable to low‐intensity electronic transitions. Clear bisignate (S‐shaped) Cotton effects were not observed in this region. While the overall CD response evolved with helicene length, the maximum Δε values did not increase monotonically across the series. Rather than a simple systematic amplification of rotational strength, the CD spectra reflect length‐dependent changes in the distribution and relative intensity of chiroptical transitions.

The CPL spectra of **TA[7]–[15]H** (Figure 5b) closely parallel their fluorescence profiles, with mirror‐image signals of opposite sign observed for each pair of enantiomers. The emission dissymmetry factors (*g*_lum_) increase markedly with helicene length—0.0087 (**TA[7]H**), 0.012 (**TA[9]H**), 0.026 (**TA[11]H**), 0.022 (**TA[13]H**), and 0.028 (**TA[15]H**)—revealing a clear correlation between helical expansion and CPL activity. Notably, **TA[11]H** already displays a sharp rise in *g*_lum_ (0.026), marking the onset of strong chiroptical amplification. When combined with the fluorescence quantum yields (*Φ*_F_), these *g*_lum_ values define the overall CPL figures of merit (*Φ*_F_ × *g*_lum_). The highest values are obtained for **TA[7]H** and **TA[15]H**, due to their synergistic combination of high *Φ*_F_ (0.39 and 0.36, respectively) and sizable *g*_lum_. In contrast, intermediate helicenes, such as **TA[9]H** and **TA[13]H**, suffer from reduced *Φ*_F_, which suppresses their CPL efficiencies despite respectable *g*_lum_ values. These results demonstrate that both radiative efficiency and electronic asymmetry must be optimized simultaneously, and highlight **TA[15]H** as one of the most efficient helicene‐based CPL emitters reported to date.

### Role of Transition Dipole Orientations in Governing *g*‐Value Enhancement

**Figure 6:**
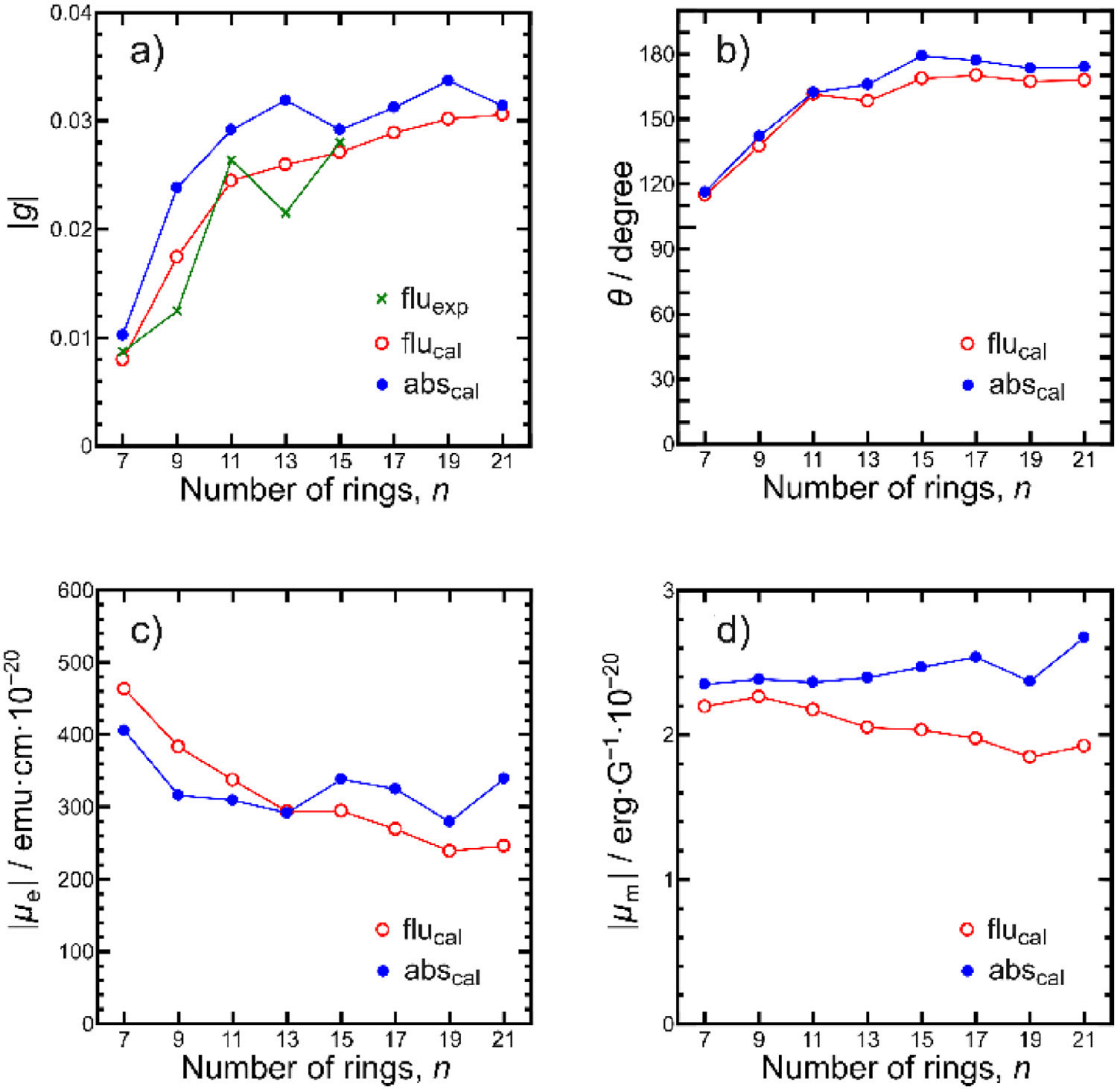
Chiroptical properties of **TA[*n*]H** molecules for different [*n*] values. a) Calculated *g* values for absorption (blue closed circles) and fluorescence (red open circles), and experimental fluorescence *g* values (green crosses). b) Angle *θ* between the electric (*µ*_e_) and magnetic (*µ_m_*) transition dipole moments. c) Magnitude of the electric transition dipole moment |*µ*_e_|. d) Magnitude of the magnetic transition dipole moment |*µ_m_* |. Calculations were performed at the TD‐DFT level (PBE0/6–31G(d)/SMD) based on S_0_ and S_1_ optimized structures.

To understand the evolution of chiroptical properties across the series, we analyzed the electric (*µ*_e_) and magnetic (*µ_m_*) transition dipole moments obtained from TD‐DFT calculations. This type of *µ*_e_/*µ_m_*‐based analysis has been widely adopted in helicene studies.^[^ ^32^, ^36^ ^]^ Vectorial analysis of transition dipole moments provides a clear rationale for the nonlinear increase in CPL dissymmetry factors. Transition dipole moments were obtained from TD‐DFT calculations at the S_0_ geometry for absorption and at the S_1_ geometry for fluorescence, and the *g* values were estimated according to Equation (1):

(1) $$g=\frac{4\left|\mu_{m}\right|\left|\mu_{e}\right|}{{\left|\mu_{m}\right|}^{2}+{\left|\mu_{e}\right|}^{2}}\;\cos\left(\theta \right)$$

where *θ* is the angle between *µ*_e_ and *µ*_m_. As shown in Figure 6a, the calculated fluorescence *g* values reproduce the experimental trend, exhibiting a sharp increase up to **TA[11]H**, followed by a more gradual rise that ultimately saturated around *g*_lum_ ≈ 0.03.

**Figure 7:**
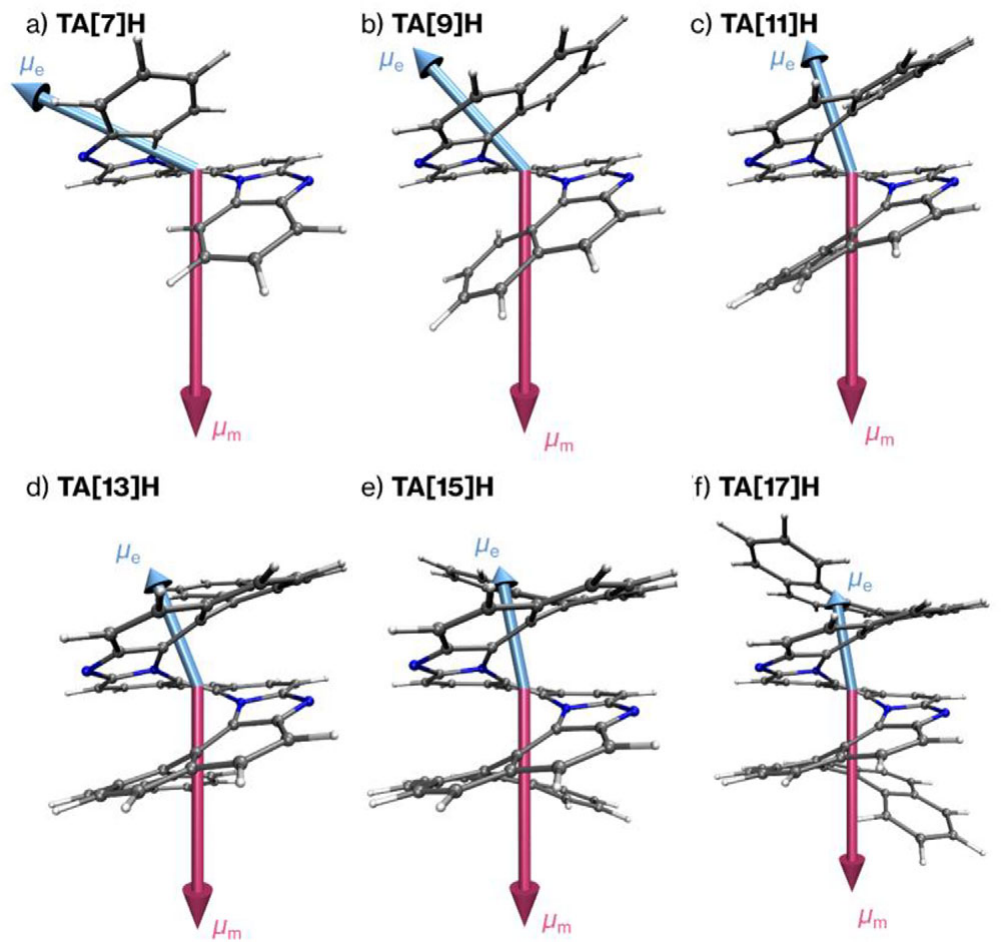
Visualization of the electric (*µ*_e_, light blue arrow) and magnetic (*µ_m_*, pink arrow) transition dipole moment vectors for the fluorescence of **TA[*n*]H**. Calculations were performed at the TD‐DFT level (PBE0/6–31G(d)/SMD) based on the S_1_ optimized structure.

Figure 6c,d show a moderate (≤ 2×) change in |*µ*_e_| and only minor changes in |*µ_m_* |. While the change in |*µ*_e_| affects *g* as in Equation (1), it explains only part of the trend; the sustained amplification of the *g* value is predominantly due to improved *θ*: *θ* increases rapidly with *n* and approaches ∼180° (close to antiparallel alignment) (Figure 6b). Thus, while the reorientation of transiton dipoles (*θ*) is the dominant factor governing enhanceent of the *g* value, the moderate variation in |*µ*_e_| also provides a secondary contribution that should not be overlooked. A similar strategy of vectorial control of transition dipole moments has been demonstrated in multihelical systems.^[^ ^28^ ^]^ Remarkably, the direction of µ_m_ remained consistently along the helical axis forall **TA[*n*]H** systems, whereas *µ*_e_ progressively reoriented toward this axis with increasing *n* (Figure 7 for fluorescence, Figure S7 for absorption). For instance, in **TA[7]H**, *µ*_e_ formed an oblique angle with the helical axis (*θ* ≈ 115° for fluorescence and 116° for absorption), indicating that it was far from an antiparallel orientation. With increasing *n*, *µ*_e_ progressively reoriented toward the helical axis, leading to near‐antiparallel alignment with *µ_m_* at *n* ≥ 11. These results demonstrate that the *n*‐dependent enhancement of *g* values arises primarily from dipole reorientation (*θ*), with dipole magnitude variations playing only a secondary role. The structural origins of this reorientation will be discussed in detail in the following section.

The calculated *g*_lum_ at the PBE0/6–31G(d)/SMD level reproduces the experimental values within the range of 0.91–1.40 for the series studied. This indicates an overall agreement in the order of magnitude. However, the calculations tend to overestimate the largest *g*_lum_ by up to ≈ 40% in some compounds. To illustrate the degree of agreement, Table S4Ta lists the experimental and calculated *g*_lum_ values along with their ratios, and Figure S8 shows the correlation plot (calc versus exp) with a linear fit (slope = 1.03, *R* ^2^ = 0.980) and RMSE = 0.00306. Such deviations can be attributed to several sources: (i) the intrinsic limitations of TD‐DFT and the particular choice of functional and basis set, (ii) the continuum solvation model which neglects specific solute–solvent interactions and local field effects, and (iii) experimental uncertainties associated with measurements at very low concentrations (signal/noise and instrument calibration).

The robustness of the results was verified by additional tests. Optimizations at the B3LYP/6–31G(d)/SMD and CAM‐B3LYP/6–31G(d)/SMD levels, as well as single‐point TD‐DFT with larger basis sets and functionals (PBE0/def2‐TZVP/SMD, PBE0/6–311 + G(d,p)/SMD, and CAM‐B3LYP/def2‐TZVP/SMD), reproduced the same dipole reorientation and *g*‐value trends. Gas‐phase calculations likewise confirmed that solvent effects do not alter the qualitative conclusions (see Supporting Information, Section S9). We note that TD‐DFT calculations tend to overestimate absolute *g* values; achieving quantitative accuracy will require higher‐level approaches, which are beyond the scope of the present study, but represent an important direction for future work.

**Figure 8:**
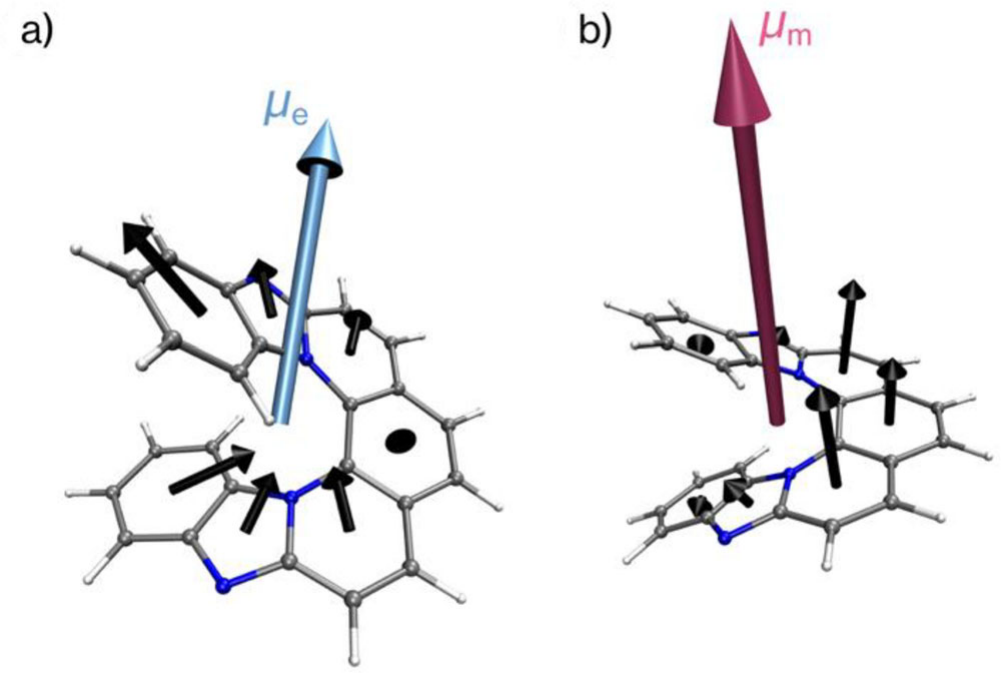
(a) Contributions of each aromatic ring in **TA[7]H** to the electric (*µ*_e_) and (b) magnetic (*µ_m_*) transition dipole moments, indicated by black arrows. The contribution of each atom shared by two aromatic rings was weighted by 0.5. All transition dipole vectors, including both the individual ring contributions (black arrows) and the overall transition dipole moments of **TA[7]H**, *µ*_e_ (light blue) and *µ_m_* (pink), were evaluated by TD‐DFT (PBE0/6–31G(d)/SMD) at the S_1_‐optimized geometry.

In Figure 8, the black arrows represent the contributions of each aromatic ring to the transition dipole moments, obtained from TD‐DFT calculations based on the S_1_ structure and decomposed using Multiwfn 3.7,^[^ ^39^ ^]^ with (a) for *µ*_e_ and (b) for *µm*. In (a), most black arrows are oriented nearly within the planes of the corresponding rings, which is consistent with the characteristic nature of a π–π* transition, although they are not perfectly coplanar. In contrast, in (b), the black arrows are predominantly aligned almost perpendicular to the ring planes, reflecting the helical π‐electron motion in **TA[*n*]H**; such solenoidal electron displacement is an intrinsic feature of the helicene architecture and has also been highlighted by Miguel and co‐workers.^[^ ^28^ ^]^

Closer inspection of (b) reveals that the largest contributions originate from the aza‐phenanthroline core, where the LUMO is mainly localized. These arrows are both large and clearly perpendicular to the local ring planes, whereas those on the peripheral rings are much smaller and largely tilted. A few arrows lie closer to the ring planes, but their magnitudes are very small and they can be neglected, because these rings have only minor LUMO contributions. This distribution fully supports the scenario of a helical electron displacement along the π‐framework, leading to *µm* being oriented nearly antiparallel to the helical axis regardless of *n*, as shown in Figures 6b and 7. In contrast, *µ*_e_, which originates from in‐plane contributions, varies significantly with *n*: in short helicenes, the overall *µ*_e_ is tilted relative to the helical axis, but as *n* increases, the vector becomes progressively aligned with the axis due to cancellation of lateral components, in agreement with the trend illustrated in Figures 6b and 7.

### Quantitative Performance

For comparison of the figures of merit (*Φ*_F_ × *g*_lum_ (FOM)) and CPL brightness (*εΦ*_F _× *g*_lum_), the interplay between fluorescence efficiency and dissymmetry factors is crucial. In the present study, *Φ*_F_ and *ε* were determined at an excitation wavelength of 305 nm, corresponding to the absorption maximum, whereas *g*_lum_ values were obtained from CPL measurements recorded at 380 nm, which was selected as a representative excitation wavelength to ensure consistent and reliable measurements across the homologous series. Because *g*_lum_ is generally only weakly dependent on excitation wavelength within a given electronic transition, the resulting FOM and CPL brightness values can be regarded as approximate (semi‐quantitative) metrics for comparing relative CPL performance within this homologous series.

Whereas intermediate homologues such as **TA[9]H** and **TA[13]H** exhibit reduced *Φ*_F_ values (0.15 and 0.14, respectively), both **TA[7]H** and **TA[15]H** retain high *Φ*_F_ values (0.39 and 0.36) together with sizable *g*_lum_ values, leading to outstanding overall CPL outputs. In particular, **TA[15]H** exhibits a high molar absorptivity at the fluorescence excitation wavelength (*ε* = 4.9 × 10^4^ L mol^−^^1^ cm^−^^1^ at 305 nm) together with a high fluorescence quantum yield (*Φ*_F_ = 0.36) and a sizable CPL dissymmetry factor (*g*_lum_ = 0.028), resulting in an FOM of 0.010 and a CPL brightness of approximately 490. This value far surpasses those of thiadiazole‐based helicenes (FOM ≈ 0.0004), despite their larger *g*_lum_ values (≈ 0.04) but very low fluorescence quantum yields (*Φ*_F_ ≈ 0.01).^[^ ^40^ ^]^

This analysis highlights that maximizing CPL performance requires a delicate balance between radiative and non‐radiative pathways. Intermediate homologues suffer from enhanced non‐radiative decay, whereas the shortest and longest members benefit from more rigidified π‐frameworks that suppress such losses. Consequently, the tetraazahelicene series uniquely combines high fluorescence quantum yields with substantial dissymmetry, affording practical CPL outputs unmatched by existing helicenes.

Carbohelicenes typically display modest *g* values (∼10^−3^) that further diminish with increasing helical length.^[^ ^41^ ^]^ Pittelkow's azaoxahelicenes^[^ ^14^ ^]^ extend up to **OA[13]H** but remain weak emitters, whereas Tanaka's benzannulated azahelicenes^[^ ^13^ ^]^ reach **PA[19]H** yet exhibit diluted CPL activity (Figure 1) due to partially planarized segments. Other heteroatom‐bridged helicenes show improved dissymmetry but still suffer from low fluorescence efficiency, underscoring the exceptional balance achieved by tetraazahelicenes.

## Conclusion

We synthesized a homologous series of tetraaza[7]–[15]helicenes (**TA[7]–[15]H**) via a concise two‐step strategy. Their optical spectra converge beyond **TA[11]H**, defining a conjugation ceiling, whereas their chiroptical responses amplify sharply, yielding *g*_lum_ values up to 0.028 and outstanding, albeit semi‐quantitative, CPL performance, with figures of merit reaching 0.010 and CPL brightness values of approximately 490. TD‐DFT calculations revealed that this amplification originates from the delayed alignment of electric and magnetic transition dipoles, while ^1^H NMR chemical shifts of inner protons independently probed structural reorganization within the helical cavity. Importantly, this critical behavior is corroborated by independent probes: the absorption maxima rapidly saturate, the inner ^1^H NMR protons resonate progressively upfield with length, and CPL dissymmetry factors increase sharply beyond **TA[11]H**. Together, these orthogonal properties consistently identify **TA[11]H** as the critical helicene length at which the framework undergoes a qualitative transition in its physical environment. Notably, **TA[15]H**, together with Tanaka's **PA[15]H** (R = Bu, Figure 1), constitutes the longest helicene species ever resolved into its enantiomers. Compared with carbo‐, expanded, and other heterohelicenes, tetraazahelicenes uniquely combine solubility, rigidity, efficiency, and strong dissymmetry, establishing them as benchmarks for chiral optoelectronic materials. Most importantly, this study demonstrates that helicene length *n* serves as a universal design parameter to direct transition dipole orientations toward near‐antiparallel alignment, thereby enabling systematic enhancement of chiroptical responses.

## Conflict of Interests

The authors declare no conflicts of interest.

## Supporting information



Supporting Information

Supporting Information

## Data Availability

The data that support the findings of this study are available in the Supporting Information of this article.
