# Supplementary material for: Tetraaza[7]–[15]helicenes Synthesized by Two‐Step Strategy: Length‐Controlled Chiral π‐Systems Exhibiting Amplified Circularly Polarized Luminescence
Source: Angew Chem Int Ed Engl. 2026 Jan 9;65(14):e24463. doi: 10.1002/anie.202524463 (PMC13023689; doi:10.1002/anie.202524463)
Supplement: Supplementary file 1 — Supporting Information [file ANIE-65-e24463-s002.pdf]

Supporting Information

Tetraaza[7]–[15]helicenes Synthesized by Two-Step Strategy: Length-Controlled Chiral π-Systems Exhibiting Amplified Circularly Polarized Luminescence

Takashi Otani,*[a] Yuchen Wu,[b] Kohei Ueda,*[a] Yuki Ikeda,[a] Yuna Tada,[a] Natsuna Kinoshita,[a] and Takanori Shibata*[b]

[a] Course of Chemical Engineering, National Institute of Technology Anan College, 265 Aoki, Minobayashi, Anan, Tokushima 774-0017, Japan

[b] Department of Chemistry and Biochemistry, School of Advanced Science and Engineering, Waseda University, 3-4-1 Okubo, Shinjuku, Tokyo 169-8555, Japan

Table of Contents:

A. Experimental Procedures S2

B. Theoretical Calculation S12

C. References S35

D. Copies of ^{1}H and ^{13}C NMR spectra of new compounds S36

A. Experimental Procedures

2,9-Dichloro-1,10-phenanthroline (**1**) was purchased from Tokyo Chemical Industry Co., Ltd and BLD Pharmatech Ltd. 3-Chloroperoxybenzoic acid (*m*CPBA, ca. 70% purity containing ca. 30% water, Tokyo Chemical Industry Co., Ltd.) was used as received, and the molar amounts were calculated on the basis of 70% purity. Palladium on activated charcoal (10 wt% Pd) was purchased from Merck and used as received. The other reagents were purchased from Tokyo Chemical Industry Co., Ltd, Wako Pure Chemical Industries, Ltd., Kanto Chemical Co., Inc., KISHIDA CHEMICAL Co., Ltd., and Sigma-Aldrich Co. LLC. Preparative thin-layer chromatography (PTLC) was performed with silica gel-precoated glass plates prepared in our laboratory. Silica-gel column chromatography was carried out using Silica Gel 60 N (Kanto Chemical Co., spherical, neutral, 0.040–0.050 mm).

^{1}H NMR spectra were recorded on a JEOL ECZ-400S (400 MHz) or a JEOL ECS-400 (400 MHz) spectrometer. The chemical shifts were reported in parts per million (δ) relative to internal standard TMS (0 ppm). Data are reported as follows: chemical shift, multiplicity (s = singlet, d = doublet, t = triplet, dd = doublet of doublet, m = multiplet), coupling constant (Hz), and integration. ^{13}C NMR spectra were obtained by a JEOL ECZ-400S (100 MHz) or a Bruker Avance-600 (150 MHz) spectrometer and referenced to the internal solvent signals (the central peak of the $CDCl_3$ triplet (77.0 ppm)). High-resolution mass spectra (HRMS) were recorded on a JMS-SX102A, a JMS-HX110, a Bruker Daltonics microTOF, a Thermo Fisher Scientific Exactive Plus equipped with a Direct Analysis in Real Time (DART) ion source, or a Bruker QTOF compact (positive mode) equipped with an Atmospheric Pressure Chemical Ionization (APCI) source using the Orbitrap mass spectrometer method.

UV-vis spectra were measured on a JASCO V-630 photometer. Fluorescence spectra were taken on a JASCO FP-8200 spectrofluorometer. Absolute PL quantum yield was measured by Hamamatsu Photonics C9920-02 spectrometer, and quantum yields were determined with an integrating sphere (diameter 10 cm).

Circular dichroism spectra were obtained using a JASCO J-820 (420 W Xe) spectropolarimeter and CPL spectra were obtained using a JASCO CPL-200 at room temperature.

2-Naphthylamine (2)[S1]

Caution: 2-Naphthylamine is a potentially hazardous compound. All manipulations should be performed in a well-ventilated fume hood while wearing appropriate personal protective equipment (PPE).

A solution of *N*-benzyl-2-naphthylamine (233 mg, 1.0 mmol) in ethyl acetate/methanol (v/v = 1:1, 10 mL) was treated with conc. HCl (20 μL) and 10 % Pd/C (23 mg). The mixture was stirred under a hydrogen atmosphere (1 atm) for 4 h. After completion, the mixture was filtered through a Celite pad and concentrated under reduced pressure. The residue was dissolved in ethyl acetate (5 mL) and washed with saturated aqueous $NaHCO_3$ (3 mL). The organic layer was dried over anhydrous Na_2SO_4, filtered, and concentrated to afford 2-naphthylamine (**2**) (140 mg, 98%) as a colourless solid, which was used without further purification.

N^2,N^9-Di(naphthalen-2-yl)-1,10-phenanthroline-2,9-diamine (8)

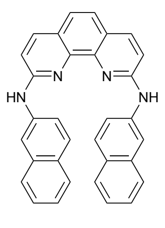

A mixture of 2,9-dichloro-1,10-phenanthroline (100 mg, 0.40 mmol), 2-naphthylamine (140 mg, 0.98 mmol, 2.5 equiv), and *n*-butanol (5 mL) was heated at 120 °C for 6 h. After cooling to room temperature, the reaction mixture was diluted with saturated aqueous $NaHCO_3$ and extracted with chloroform. The combined organic layers were dried over anhydrous Na_2SO_4, filtered, and concentrated under reduced pressure. The crude residue was purified by column chromatography on silica gel using chloroform as the eluent to afford the desired compound **8** as a colourless solid (170 mg, 0.37 mmol, 92%).

^{1}H-NMR (400 MHz, $CDCl_3$): δ 8.05 (d, J = 8.8 Hz, 2H), 7.84 (d, J = 1.4 Hz, 2H), 7.82 (d, J = 8.5 Hz, 2H), 7.80 (d, J = 8.0 Hz, 2H), 7.74 (d, J = 8.2 Hz, 2H), 7.51 (s, 2H), 7.48–7.44 (m, 4H), 7.43–7.37 (m, 4H).

^{13}C-NMR (100 MHz, $CDCl_3$): δ 154.83 (C), 144.49 (C), 138.23 (C), 137.85 (C), 134.25 (CH), 130.38 (C), 129.37 (CH), 127.68 (CH), 127.07 (CH), 126.51 (CH), 124.58 (CH), 124.44 (C), 122.61 (CH), 121.76 (CH), 116.51 (CH), 110.94 (CH).

HRMS-ESI (*m*/*z*): calcd. for $C_{32}H_{23}N_4$ $[M+H]^+$ 463.1917, found 463.1913.

N^2,*N*^{9}-Di(phenanthren-3-yl)-1,10-phenanthroline-2,9-diamine (9)

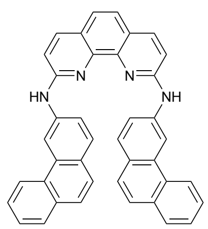

A mixture of 2,9-dichloro-1,10-phenanthroline (75 mg, 0.30 mmol), phenanthren-3-amine[S2] (198 mg, 1.02 mmol, 3.4 equiv), and *n*-butanol (2 mL) was heated at 140 °C for 10 h. After cooling to room temperature, the reaction mixture was diluted with saturated aqueous $NaHCO_3$ and extracted with chloroform. The combined organic layers were dried over anhydrous Na_2SO_4, filtered, and concentrated under reduced pressure. The crude product was purified by column chromatography on silica gel using chloroform as the eluent to afford the desired compound **9** as a yellow solid (168 mg, 0.30 mmol, quantitative yield).

^{1}H-NMR (400 MHz, $CDCl_3$): δ 9.18 (brs, 2H), 8.78 (d, J = 8.8 Hz, 2H), 8.09 (d, J = 8.5 Hz, 2H), 7.88 (d, J = 8.5 Hz, 2H), 7.85 (d, J = 8.1 Hz, 2H), 7.71 (d, J = 8.9 Hz, 2H), 7.66 (d, J = 8.8 Hz, 2H), 7.60 (ddd, J = 1.5, 7.6, 7.6 Hz, 2H), 7.55 (s, 2H), 7.54 (ddd, J = 1.5, 7.6, 7.6 Hz, 2H), 7.49 (dd, J = 1.5, 7.6 Hz, 2H), 7.41 (d, J = 8.5 Hz, 2H).

^{13}C-NMR (100 MHz, $CDCl_3$): δ 154.60 (C), 144.46 (C), 138.95 (C), 138.16 (CH), 132.42 (C), 131.50 (C), 129.99 (C), 129.62 (CH), 128.57 (CH), 128.22 (CH), 126.68 (CH), 126.56 (CH), 126.40 (CH), 125.32 (CH), 124.45 (C), 123.06 (CH), 122.67 (CH), 120.61 (CH), 112.60 (CH), 111.47 (CH).

HRMS-APCI (*m*/*z*): calcd. for $C_{40}H_{27}N_4$ $[M+H]^+$ 563.2230, found 563.2209.

N^2,*N*^{9}-Bis(benzo[*c*]phenanthren-2-yl)-1,10-phenanthroline-2,9-diamine (10)

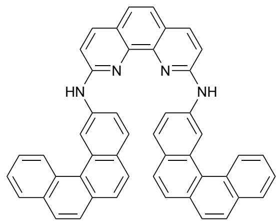

A mixture of 2,9-dichloro-1,10-phenanthroline (36 mg, 0.14 mmol), benzo[*c*]phenanthren-2-amine[S3] (77 mg, 0.32 mmol, 2.2 equiv), and *n*-butanol (1 mL) was heated at 140 °C for 23 h. After cooling to room temperature, the reaction mixture was diluted with saturated aqueous $NaHCO_3$ and extracted with chloroform. The combined organic layers were dried over anhydrous Na_2SO_4, filtered, and concentrated under reduced pressure. The crude product was purified by column chromatography on silica gel using chloroform as the eluent to afford the desired compound **10** as a brown solid (78 mg, 0.12 mmol, 83%).

^{1}H-NMR (400 MHz, $CDCl_3$): *δ* 9.06 (d, *J* = 8.2 Hz, 2H), 9.02 (s, 2H), 8.02 (d, *J* = 8.7 Hz, 2H), 7.93 (dd, *J* = 8.2, 1.8 Hz, 2H), 7.87 (d, *J* = 8.3 Hz, 2H), 7.83 (d, *J* = 8.5 Hz, 2H), 7.77 (d, *J* = 8.3 Hz, 2H), 7.76 (d, *J* = 8.4 Hz, 2H), 7.68 (d, *J* = 8.2 Hz, 2H), 7.57 (dd, *J* = 8.5, 1.8 Hz, 2H), 7.52 (ddd, *J* = 7.4, 7.4, 1.8 Hz, 2H), 7.48–7.44 (m, 4H), 7.37 (d, *J* = 8.5 Hz, 2H).

^{13}C-NMR (100 MHz, $CDCl_3$): *δ* 154.72 (C), 144.41 (C), 138.46 (C), 138.12 (CH), 133.25 (C), 131.44 (C), 131.18 (C), 130.34 (C), 130.14 (CH), 129.66 (CH), 128.45 (CH), 127.56 (CH), 127.48 (CH), 127.11 (CH), 126.83 (CH), 126.64 (C), 126.13 (CH), 125.68 (CH), 125.45 (CH), 124.38 (C), 122.51 (CH), 120.71 (CH), 118.40 (CH), 111.06 (C).

HRMS-APCI (*m*/*z*): calcd. for $C_{48}H_{31}N_4$ $[M+H]^+$ 663.2543, found 663.2517.

N^2,*N*^{9}-Bis(dibenzo[c,g]phenanthren-9-yl)-1,10-phenanthroline-2,9-diamine (11)

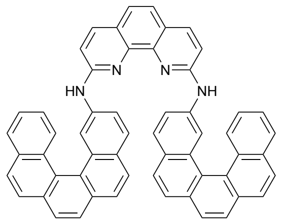

A mixture of 2,9-dichloro-1,10-phenanthroline (25 mg, 0.10 mmol), dibenzo[*c*,*g*]phenanthren-9-amine[S4] (88 mg, 0.30 mmol, 3.0 equiv), and *n*-butanol (2 mL) was heated at 140 °C for 8 h. After cooling to room temperature, the reaction mixture was diluted with saturated aqueous $NaHCO_3$ and extracted with dichloromethane. The combined organic layers were dried over anhydrous Na_2SO_4, filtered, and concentrated under reduced pressure. The residue was dissolved in a small amount of dichloromethane and passed through a short silica gel pad using dichloromethane as the eluent. After removal of the excess amine, the eluent was changed to dichloromethane/methanol (10:1) to elute the desired compound **11** as a yellow solid (76 mg, 0.10 mmol, quantitative yield).

^{1}H-NMR (500 MHz, $CDCl_3$): *δ* 8.59 (d, *J* = 8.5 Hz, 2H), 8.30 (d, *J* = 5.6 Hz, 2H), 7.97–7.77 (m, 18H), 7.59–7.51 (m, 4H), 7.38–7.32 (m, 4H), 7.11 (dd, *J* = 8.7, 4.3 Hz, 2H).

^{13}C-NMR (125 MHz, $CDCl_3$): *δ* 154.20, 144.12, 138.00, 136.85, 132.73, 132.12, 132.02, 132.00, 130.44, 129.22, 129.18, 128.94, 128.04,* 127.43,* 127.30, 127.21, 127.10, 127.07, 126.38, 126.26, 126.16, 124.88, 124.81,* 124.20, 122.36, 120.53,* 118.27, 116.56. *^{13}C signals show slight peak splitting (ca. 2 Hz), which is attributed to incomplete decoupling or minor conformational effects. Each was treated as a single resonance for the purpose of assignment.

HRMS-DART (*m*/*z*): calcd. for $C_{56}H_{35}N_4$ $[M+H]^+$ 763.2856, found 763.2848.

Naphtho[2',1':4,5]imidazo[1,2-*a*]naphtho[2',1':4,5]imidazo[2,1-*k*][1,10]phenanthroline (TA[9]H).

To a solution of compound **8** (40 mg, 0.086 mmol) and 4-iodoanisole (201 mg, 0.86 mmol, 10 equiv) in 1,1,1,3,3,3-hexafluoropropan-2-ol (HFIP, 2 mL) was added *m*-chloroperbenzoic acid (*m*CPBA, 69 mg, 0.26 mmol, 3 equiv) in six portions at 15-min intervals. After the final addition, the reaction was stirred until completion and then quenched by the addition of saturated aqueous Na_2CO_3. The mixture was extracted with chloroform, and the combined organic layers were dried over anhydrous Na_2SO_4, filtered, and concentrated under reduced pressure. The residue was purified by column chromatography on silica gel using chloroform as the eluent to afford **TA[9]H** as a yellow solid (20 mg, 0.044 mmol, 50%).

^{1}H-NMR (400 MHz, $CDCl_3$): *δ* 8.22 (s, 2H), 8.13 (d, *J* = 9.1 Hz, 2H), 8.02 (d, *J* = 9.1 Hz, 2H), 7.53 (d, *J* = 7.9 Hz, 2H), 7.29 (s, 4H), 7.20 (d, *J* = 7.1 Hz, 2H), 6.76 (ddd, *J* = 8.4, 8.4, 1.1 Hz, 2H), 5.92 (d, *J* = 8.3 Hz, 2H).

^{13}C-NMR (100 MHz, $CDCl_3$): *δ* 147.62, 141.32, 128.36, 128.01, 127.97, 127.38, 126.36, 126.04, 125.41, 124.96, 124.84, 123.77, 122.70, 117.72, 117.40, 116.87.
HRMS-ESI (*m*/*z*): calcd. for $C_{32}H_{19}N_4$ $[M+H]^+$ 459.1604, found 459.1593.

Phenanthro[3',4':4,5]imidazo[1,2-*a*]phenanthro[3',4':4,5]imidazo[2,1-*k*][1,10]phenanthroline (TA[11]H).
To a solution of compound **9** (170 mg, 0.30 mmol) and 4-iodoanisole (704 mg, 3.0 mmol, 10 equiv) in HFIP (4 mL) was added *m*-chloroperbenzoic acid (*m*CPBA, 228 mg, 0.93 mmol, 3.1 equiv) in six portions at 15-min intervals. After the final addition, the reaction was stirred until completion and then quenched by the addition of saturated aqueous Na_2CO_3. The mixture was extracted with chloroform, and the combined organic layers were dried over anhydrous Na_2SO_4, filtered, and concentrated under reduced pressure. The crude residue was purified by column chromatography on silica gel using chloroform as the eluent to afford **TA[11]H** as a brown solid (88 mg, 0.16 mmol, 52%).
^{1}H-NMR (400 MHz, $CDCl_3$): *δ* 8.24 (s, 2H), 7.90 (d, *J* = 9.0 Hz, 2H), 7.80 (dd, *J* = 0.7, 7.8 Hz, 2H), 7.59 (d, *J* = 8.6 Hz, 2H), 7.55 (d, *J* = 7.4 Hz, 2H), 7.41 (d, *J* = 8.5 Hz, 2H), 7.41 (d, *J* = 8.5 Hz, 2H), 7.38 (d, *J* = 9.0 Hz, 2H), 7.27 (ddd, *J* = 1.1, 7.0, 7.0 Hz, 2H), 6.24 (ddd, *J* = 1.3, 7.0, 7.0 Hz, 2H), 5.76 (d, *J* = 8.3 Hz, 2H).
^{13}C-NMR (100 MHz, $CDCl_3$): *δ* 146.90, 141.40, 130.97, 128.18, 128.08, 127.28, 126.87, 126.73, 126.17, 125.86, 125.54, 125.05, 124.48, 124.36, 124.31, 123.83, 123.00, 118.55, 118.52, 116.87.
HRMS-ESI (*m*/*z*): calcd. for $C_{40}H_{23}N_4$ $[M+H]^+$ 559.1917, found 559.1920.

Benzo[5',6']phenanthro[3',4':4,5]imidazo[1,2-*a*]benzo[5',6']phenanthro[3',4':4,5]imidazo[2,1-*k*][1,10]phenanthroline (TA[13]H).
To a solution of compound **10** (31 mg, 0.046 mmol) and 4-iodoanisole (108 mg, 0.46 mmol, 10 equiv) in HFIP (1 mL) was added *m*-chloroperbenzoic acid (*m*CPBA, 31 mg, 0.13 mmol, 2.8 equiv) in six portions at 15-min intervals. After the final addition, the reaction was stirred until completion and then quenched by the addition of saturated aqueous Na_2CO_3. The mixture was extracted with chloroform, and the combined organic layers were dried over anhydrous Na_2SO_4, filtered, and concentrated under reduced pressure. The crude residue was purified by column chromatography on silica gel using chloroform as the eluent to afford **TA[13]H** as a brown solid (13 mg, 0.020 mmol, 43%).
^{1}H-NMR (400 MHz, $CDCl_3$): *δ* 7.88 (d, *J* = 8.6 Hz, 2H), 7.72 (d, *J* = 8.6 Hz, 2H), 7.70 (d, *J* = 8.6 Hz, 2H), 7.69 (d, *J* = 8.6 Hz, 2H), 7.62 (d, *J* = 8.4 Hz, 2H), 7.58 (d, *J* = 8.4 Hz, 2H), 7.49 (d, *J* = 8.2 Hz, 2H), 7.48 (s, 2H), 7.46 (d, *J* = 8.6 Hz, 2H), 7.39 (d, *J* = 7.6 Hz, 2H), 6.80 (ddd, *J* = 1.2, 7.0, 7.6 Hz, 2H), 5.62 (ddd, *J* = 1.2, 6.8, 8.4 Hz, 2H), 5.25 (d, *J* = 8.4 Hz, 2H).
^{13}C-NMR (100 MHz, $CDCl_3$): *δ* 145.16 (C), 141.28 (C), 130.14 (C), 129.52 (C), 127.77 (C), 127.68 (C), 127.11 (C), 126.89 (CH), 126.72 (CH), 126.34 (CH), 126.32 (CH), 125.93 (CH), 125.76 (CH), 125.63 (C), 125.37 (CH), 124.22 (CH), 124.01 (CH), 123.75 (C), 122.87 (CH), 122.33 (CH), 121.68 (C), 117.21 (CH)*, 116.31 (C). *Based on the relative intensities and integration, this signal is assigned to two overlapping carbons.

HRMS-ESI (*m/z*): calcd. for $C_{48}H_{27}N_4$ $[M+H]^+$ 659.2230, found 659.2227.

Naphtho[1'',2'':5',6']phenanthro[3',4':4,5]imidazo[1,2-a]naphtho[1'',2'':5',6']phenanthro[3',4':4,5]imidazo[2,1-*k*][1,10]phenanthroline (TA[15]H)

To a solution of compound **11** (38.1 mg, 0.050 mmol) and 4-iodoanisole (117 mg, 0.50 mmol, 10 equiv) in HFIP (1 mL) was added *m*-chloroperbenzoic acid (*m*CPBA, 37.2 mg, 0.15 mmol, 3.0 equiv) in six portions at 15-min intervals. After the final addition, the reaction was stirred until completion and then quenched by the addition of saturated aqueous Na_2CO_3. The mixture was extracted with dichloromethane, and the combined organic layers were dried over anhydrous Na_2SO_4, filtered, and concentrated under reduced pressure. The crude residue was purified by preparative thin-layer chromatography (TLC) using dichloromethane as the eluent to afford **TA[15]H** as a yellow solid (14.5 mg, 0.019 mmol, 38%).

^{1}H-NMR (400 MHz, $CDCl_3$): δ 7.93 (d, J = 8.1 Hz, 2H), 7.79 (d, J = 8.1 Hz, 2H), 7.74 (d, J = 8.1 Hz, 2H), 7.65 (d, J = 7.7 Hz, 2H), 7.63 (d, J = 7.7 Hz, 2H), 7.58 (d, J = 8.1 Hz, 2H), 7.211 (d, J = 8.8 Hz, 2H), 7.209 (s, 2H), 7.17 (d, J = 8.2 Hz, 2H), 7.12 (d, J = 8.8 Hz, 2H), 6.96 (d, J = 8.1 Hz, 4H), 6.55 (ddd, J = 7.0, 7.0, 1.0 Hz, 2H), 5.54 (ddd, J = 7.0, 7.0, 1.0 Hz, 2H), 5.08 (d, J = 8.0 Hz, 2H).

^{13}C-NMR (150 MHz, $CDCl_3/CD_3OD$): δ 145.42, 140.14, 130.50, 129.67, 129.15, 128.74, 128.00, 127.29, 127.07, 127.02,* 126.62, 126.21, 126.08, 125.61, 125.05, 124.71, 124.65, 124.28, 123.75, 123.62, 122.75, 122.32, 121.73, 119.31, 118.10, 116.85, 116.62. *Based on the relative intensities and integration, this signal is assigned to two overlapping carbons.

HRMS-DART (*m/z*): calcd. for $C_{56}H_{31}N_4$ $[M+H]^+$ 759.2543, found 759.2536.

Chiral Resolution of TA[*n*]H

Enantiomers of **TA[*n*]H** were resolved from racemic mixtures by chiral HPLC under the following conditions.

Helicene	Column	Eluent (*v/v*)	Flow rate (mL, min^{-1})	t_{R1} (min)	t_{R2} (min)
**TA[9]H**	CHIRALPAK IE-3	EtOAc/DEA = 100/0.1	1.0	10.5	14.0
**TA[11]H**	CHIRALPAK IA	Hexane/2-propanol = 7/3	1.0	8.9	12.2
**TA[13]H**	CHIRALPAK IA	Hexane/2-propanol = 1/1	1.0	5.4	9.1
**TA[15]H**	CHIRALPAK IC	Hexane/2-propanol = 1/1	1.0	12.2	30.5

Analytical HPLC columns: 4.6 mm I.D. × 250 mm L, particle size 3 μm (Daicel).

Chiral medium-pressure liquid chromatography (CHIRALFLASH, 30 mm I.D. × 100 mm L, particle size 20 μm, flow rate 20 mL min^{-1}) was carried out using the same eluents as above to obtain enantiopure samples. For **TA[9]H**, a CHIRALFLASH IE column was used instead of IE-3.

Chiral HPLC chromatograms of **TA[9]H**: (top) rac-**TA[9]H**, (middle) (+)-**TA[9]H** (first eluted, ee > 99%), and (bottom) (−)-**TA[9]H** (second eluted, ee > 99%). The enantiomeric excess (ee) of each isolated fraction was >99%.

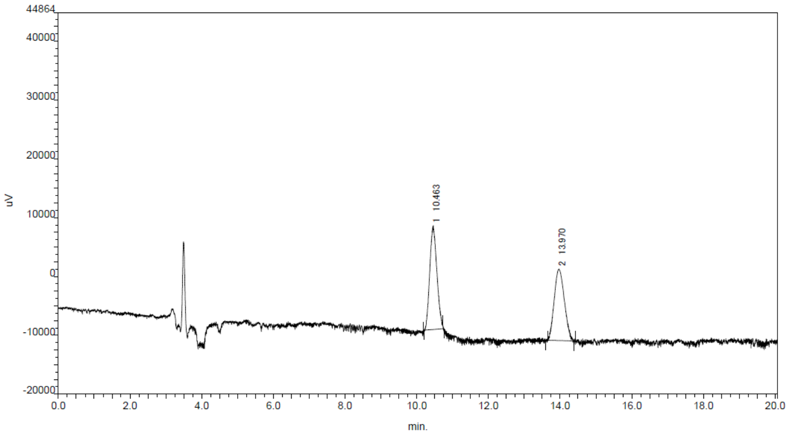

解析結果

No.	Rt(min)	ピーク名	面積	面積(%)	高さ	NTP	対称性	分離度
1	10.463		241661.2	50.3899	17655	12121.3	1.090	7.885
2	13.970		237921.7	49.6101	12157	12035.9	1.112	------
			479582.8	100.0000	29812			

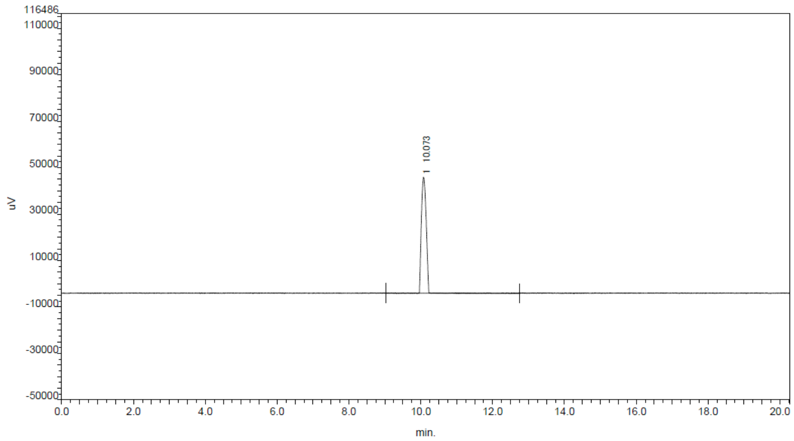

解析結果

No.	Rt(min)	ピーク名	面積	面積(%)	高さ	NTP	対称性	分離度
1	10.073		482387.5	100.0000	50087	29901.5	1.173	------
			482387.5	100.0000	50087			

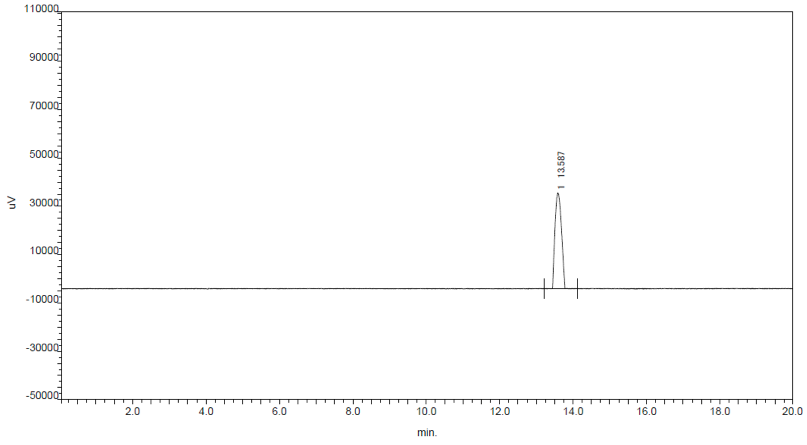

解析結果

No.	Rt(min)	ピーク名	面積	面積(%)	高さ	NTP	対称性	分離度
1	13.587		486338.8	100.0000	39673	32348.4	1.163	------
			486338.8	100.0000	39673			

Chiral HPLC chromatograms of **TA[11]H**: (top) rac-**TA[11]H**, (middle) (+)-**TA[11]H** (first eluted, ee > 99%), and (bottom) (−)-**TA[11]H** (second eluted, ee > 99%). The enantiomeric excess (ee) of each isolated fraction was >99%.

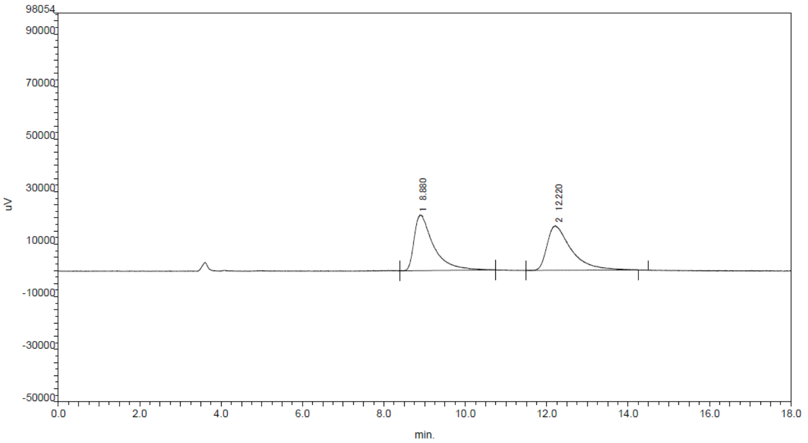

解析結果

No.	Rt(min)	ピーク名	面積	面積(%)	高さ	NTP	対称性	分離度
1	8.880		695861.8	50.0153	21247	1451.8	2.237	3.188
2	12.220		695436.7	49.9847	17032	1766.3	1.894	------
			1391298.5	100.0000	38279			

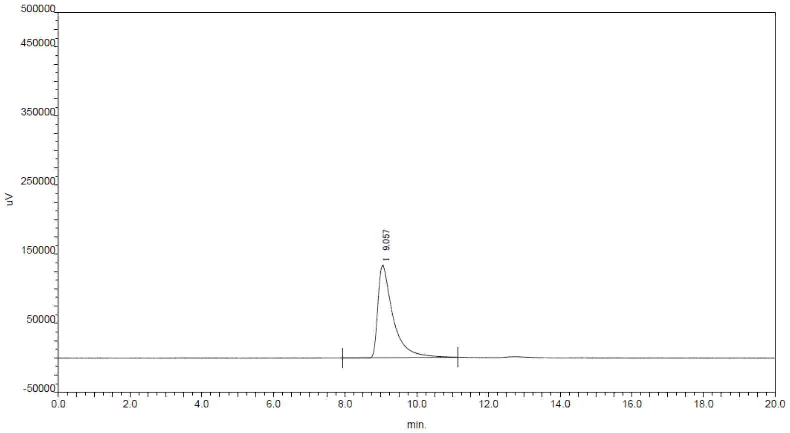

解析結果

No.	Rt(min)	ピーク名	面積	面積(%)	高さ	NTP	対称性	分離度
1	9.057		3910827.9	100.0000	134051	1936.5	2.185	------
			3910827.9	100.0000	134051			

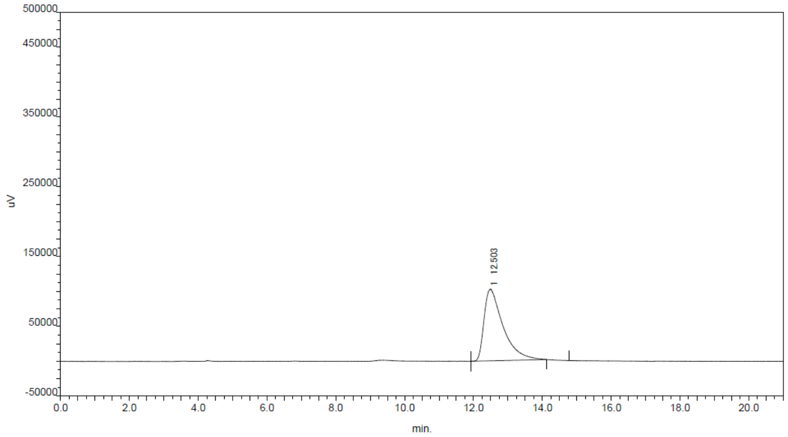

解析結果

No.	Rt(min)	ピーク名	面積	面積(%)	高さ	NTP	対称性	分離度
1	12.503		3870159.2	100.0000	102952	2131.8	1.943	------
			3870159.2	100.0000	102952			

Chiral HPLC chromatograms of **TA[13]H**: (top) rac-**TA[13]H**, (middle) (+)-**TA[13]H** (first eluted, ee > 99%), and (bottom) (−)-**TA[13]H** (second eluted, ee > 99%). The enantiomeric excess (ee) of each isolated fraction was >99%.

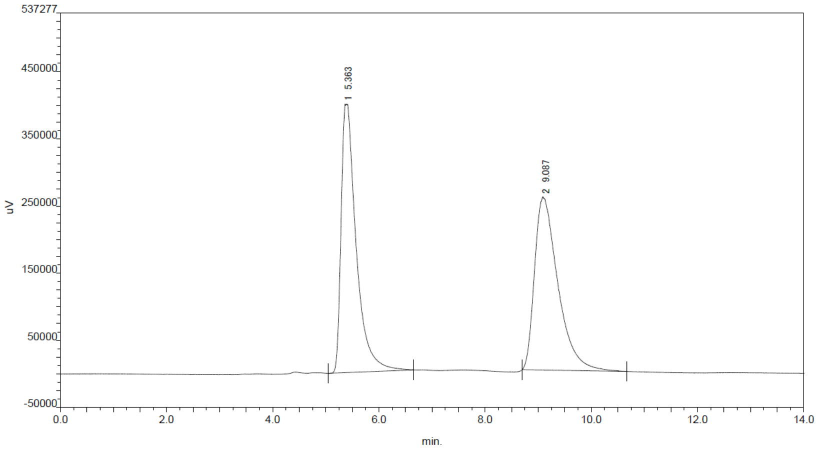

Result

No.	Rt (min)	Peak Name	Area	Area (%)	Height	NTP	TF	RS
1	5.363		7871380.2	50.0229	400088	1632.3	2.193	5.464
2	9.087		7864174.6	49.9771	257903	1908.8	1.698	------
			15735554.8	100.0000	657991			

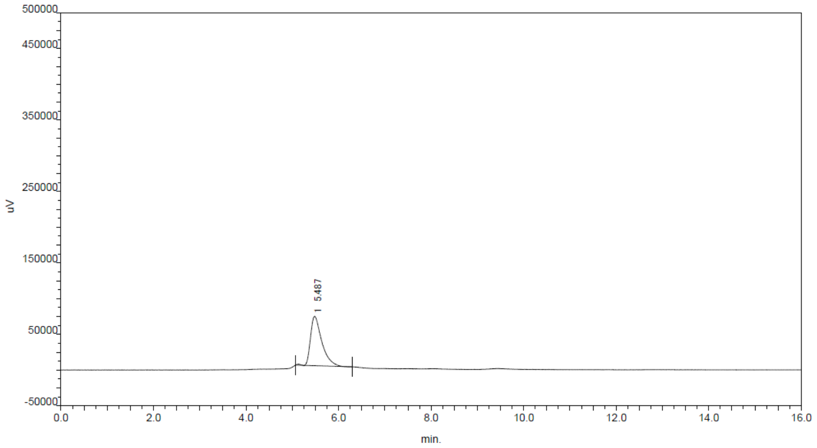

解析結果

No.	Rt (min)	ピーク名	面積	面積 (%)	高さ	NTP	対称性	分離度
1	5.487		1175804.2	100.0000	69304	2099.4	1.687	------
			1175804.2	100.0000	69304			

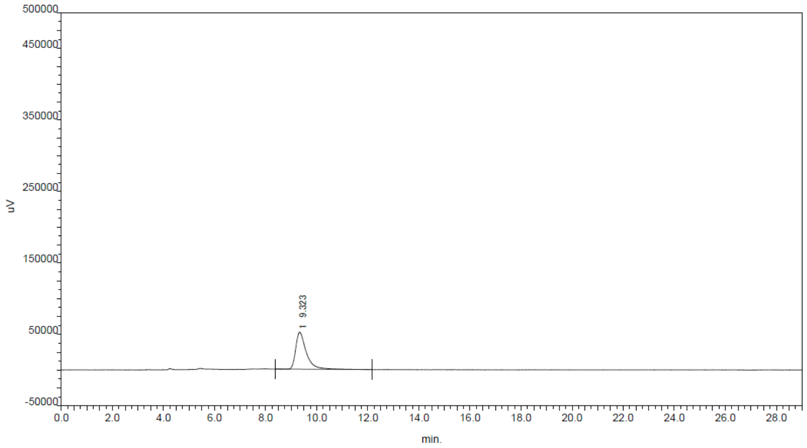

解析結果

No.	Rt (min)	ピーク名	面積	面積 (%)	高さ	NTP	対称性	分離度
1	9.323		1476197.0	100.0000	51991	2284.9	1.702	------
			1476197.0	100.0000	51991			

Chiral HPLC chromatograms of **TA[15]H**: (top) rac-**TA[15]H**, (middle) (−)-**TA[15]H** (first eluted, ee > 99%), and (bottom) (+)-**TA[15]H** (second eluted, ee > 99%). The enantiomeric excess (ee) of each isolated fraction was >99%.

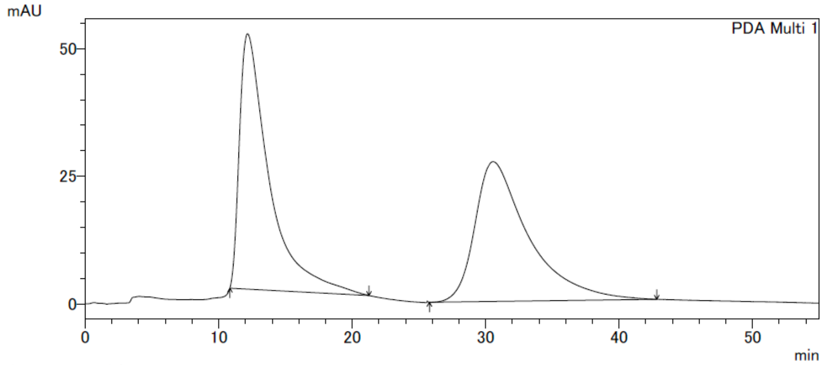

1 PDA Multi 1/254nm 4nm

PeakTable

PDA Ch1 254nm 4nm

Peak#	Ret. Time	Area	Height	Area %	Height %
1	12.162	7735758	50041	50.214	64.628
2	30.540	7669949	27389	49.786	35.372
Total		15405706	77430	100.000	100.000

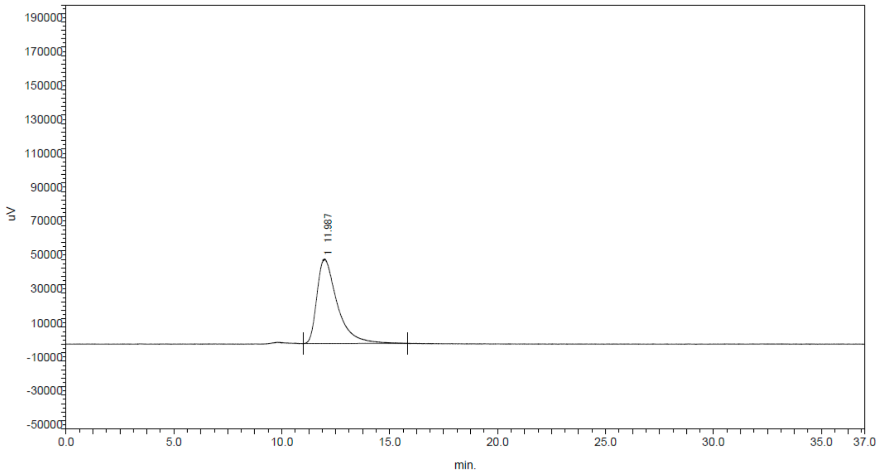

解析結果

No.	Rt (min)	ピーク名	面積	面積 (%)	高さ	NTP	対称性	分離度
1	11.987		3233543.0	100.0000	49862	720.8	1.711	------
			3233543.0	100.0000	49862			

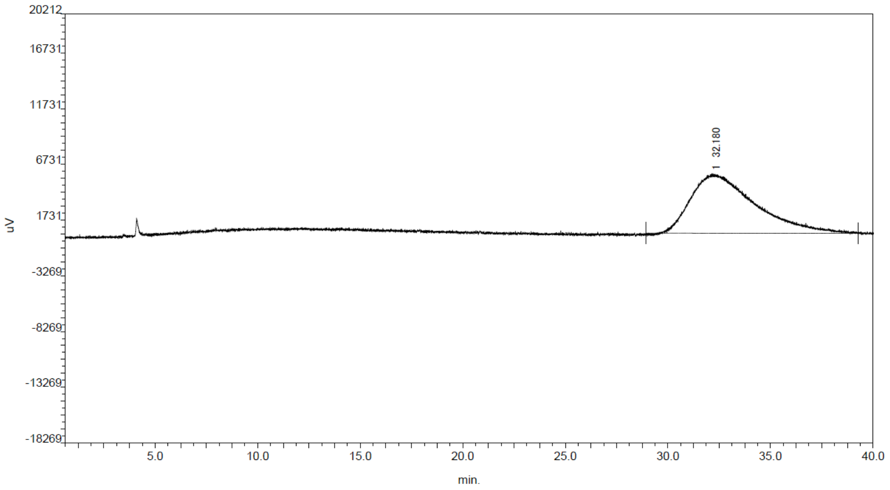

解析結果

No.	Rt (min)	ピーク名	面積	面積 (%)	高さ	NTP	対称性	分離度
1	32.180		1142589.8	100.0000	5371	469.1	1.741	------
			1142589.8	100.0000	5371			

Optical rotations were measured on a JASCO P-2200 polarimeter using a sodium lamp (λ = 589 nm) at 20 °C with a 100 mm cell (φ 3.5 mm). Concentrations (c) are given in g per 100 mL. Measurements were reproducible within an RSD of 5–11%.

Table S1. Optical rotation data for enantiopure tetraaza[n]helicenes.

TA[*n*]H	Enantiomer	Elution order	$[a]_D^{20}$
**TA[9]H**	(+)	First	+4232 (*c* 0.118, $CHCl_3$)
**TA[9]H**	(–)	Second	−4023 (*c* 0.112, $CHCl_3$)
**TA[11]H**	(+)	First	+5107 (*c* 0.146, $CHCl_3$)
**TA[11]H**	(–)	Second	−5419 (*c* 0.144, $CHCl_3$)
**TA[13]H**	(+)	First	+2196 (*c* 0.032, $CHCl_3$)
**TA[13]H**	(–)	Second	−2438 (*c* 0.064, $CHCl_3$)
**TA[15]H**	(–)	First	−7495 (*c* 0.078, $CHCl_3$)
**TA[15]H**	(+)	Second	+6735 (*c* 0.060, $CHCl_3$)

X-ray Crystallographic Analysis

Single-crystal X-ray diffraction analyses were attempted for several tetraaza[n]helicenes. In most cases, however, their high solubility and limited crystallization tendency prevented the growth of single crystals suitable for full structural refinement.

For **TA[11]H**, single crystals of sufficient quality for X-ray analysis were obtained. Diffraction data were collected and the structure was solved and refined using standard methods. Although the resulting CIF shows a CheckCIF Alert A, mainly arising from limited crystal quality and associated disorder leading to relatively high R values, the molecular connectivity and the overall helical framework are clearly and unambiguously resolved.

The crystallographic data for **TA[11]H** have been deposited with the Cambridge Crystallographic Data Centre under deposition number CCDC 2516246. An ORTEP representation of **TA[11]H** is shown in Figure S1 to provide supportive structural evidence for the helical framework.

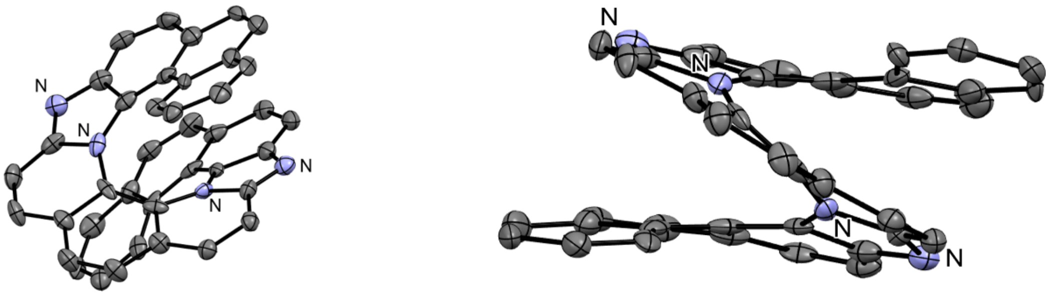

Figure S1. ORTEP representation of **TA[11]H** with thermal ellipsoids drawn at the 50% probability level. Hydrogen atoms are omitted for clarity.

B. Theoretical Calculation

S1 Overall Computational Strategy and Methods

S1.1 Overview of Computational Workflow

The computational study consists of four main components:

(i) Comparison of functionals and basis sets for absorption spectra (Section S2).

Based on these calculations, PBE0/6-31G(d) with the SMD solvent model was selected as the optimal level of theory for subsequent calculations. Supplementary figures that support the PBE0/6-31G(d)/SMD results but are not included in the main text are provided in Sections S2–S6.

(ii) NMR simulations (Sections S7 and S8).

Compared with experimental NMR data, a separate computational protocol with empirically reported scaling factors was employed. These calculations were not performed at the PBE0/6-31G(d)/SMD level.

(iii) Robustness check of observed trends in μ_e, μ_m, and g with respect to the number of rings (n) (Sections S9).

The transition dipole moments and g values discussed in the main text are based on calculations at the PBE0/6-31G(d)/SMD level. To confirm that these trends are not artifacts of the computational level, additional calculations with various functionals and basis sets were carried out (Section S9).

(iv) Additional DFT Analysis of Even-Membered **TA[*n*]H** (Section S10)

S1.2 General Computational Details

All calculations were performed using Gaussian 16 Rev. C.02.[S5] Solvent effects were modeled using the SMD polarizable continuum model (dichloromethane) unless otherwise specified. Ground-state (S_0) and first excited-state (S_1) geometries were optimized at several DFT levels, including PBE0/6-31G(d), B3LYP/6-31G(d), and CAM-B3LYP/6-31G(d). Frequency calculations were carried out at each optimized structure to confirm that they correspond to true minima with no imaginary frequencies. The Cartesian coordinates (xyz format) and vibrational frequency data for **TA[7]–[21]H** are provided in the Supporting Information zip archive. For higher-level TD-DFT calculations, such as those using larger basis sets or long-range corrected functionals (PBE0/def2-TZVP/SMD, PBE0/6-311+G(d,p)/SMD, and CAM-B3LYP/def2-TZVP/SMD), single-point energy calculations were performed on the optimized geometries. These single-point calculations were carried out for both the S_0-optimized and S_1-optimized geometries obtained at the PBE0/6-31G(d) /SMD level.

NMR simulations were performed using a separate computational protocol with empirically reported scaling factors and are independent of the optical property and g-value analyses. NMR chemical shifts were calculated by first optimizing the geometry at the B3LYP/6-31G(d) level in the gas phase, followed by the calculation of NMR shielding constants using the GIAO method at the B3LYP/6-31+G(d,p) level with the SMD($CHCl_3$) solvation model.

S1.3 Transition Dipole Moments *g* value Calculation

Electric (μ_e) and magnetic (μ_m) transition dipole moment vectors were extracted directly from the TD-DFT Gaussian output files. The Gaussian output values, given in atomic units (a.u.), were converted to cgs units prior to further calculations using the following relations: 1 a.u. of electric dipole moment = 2.54×10^{-18} esu·cm; 1 a.u. of magnetic dipole moment = 9.27×10^{-21} erg/G. g was calculated using Eq. (1) in the main text, where θ is the angle between μ_e and μ_m vectors, obtained from their Cartesian components via the scalar product.

S2 Comparison of Functionals and Basis Sets for Absorption Spectra

The performance of different density functionals was compared. Using the 6-31G(d) basis set, ground-state geometries were optimized with each functional, and TD-DFT calculations were carried out based on the optimized structures. The resulting excitation energies were used to simulate the UV–vis absorption spectra with GaussSum 3.0 [S6]. A Gaussian broadening with a full width at half maximum (FWHM) of 3000 cm^{-1} was applied. As shown in Figure S2(a), the lowest-energy absorption bands calculated with B3LYP, M06, and PBE0 were red-shifted relative to the experimental UV–vis spectrum, while those obtained with M06-2X, CAM-B3LYP, ωB97XD, and LC-ωPBE were blue-shifted. The deviation was particularly large in the case of LC-ωPBE. Among these, PBE0 reproduced the experimental results most accurately, indicating that it is the most suitable functional for the calculation of **TA[*n*]H**.

The basis set dependence was also investigated by comparing the calculated UV–vis spectra of **TA[7]H** with experimental data. As shown in Figure S2(b), when PBE0 was employed, the size of the basis set and the inclusion of empirical dispersion corrections (Grimme's D3) had only minor effects on the absorption wavelengths. In contrast, the inclusion of solvent effects consistently improved the agreement between theory and experiment. Between the SMD(CH_2Cl_2) and PCM(CH_2Cl_2) models, the SMD model yielded slightly better reproduction of the experimental spectrum. A similar trend of basis set dependence was observed with B3LYP as shown in Figure S2(c). Even with the 6-31G(d)/SMD(CH_2Cl_2) basis set, PBE0 still showed smaller deviations from experiment compared with B3LYP. Although the experimental spectrum exhibits vibronic splitting, which cannot be captured by TD-DFT, the overall spectral profile calculated with PBE0/6-31G(d)/SMD(CH_2Cl_2) showed the best agreement with experiment.

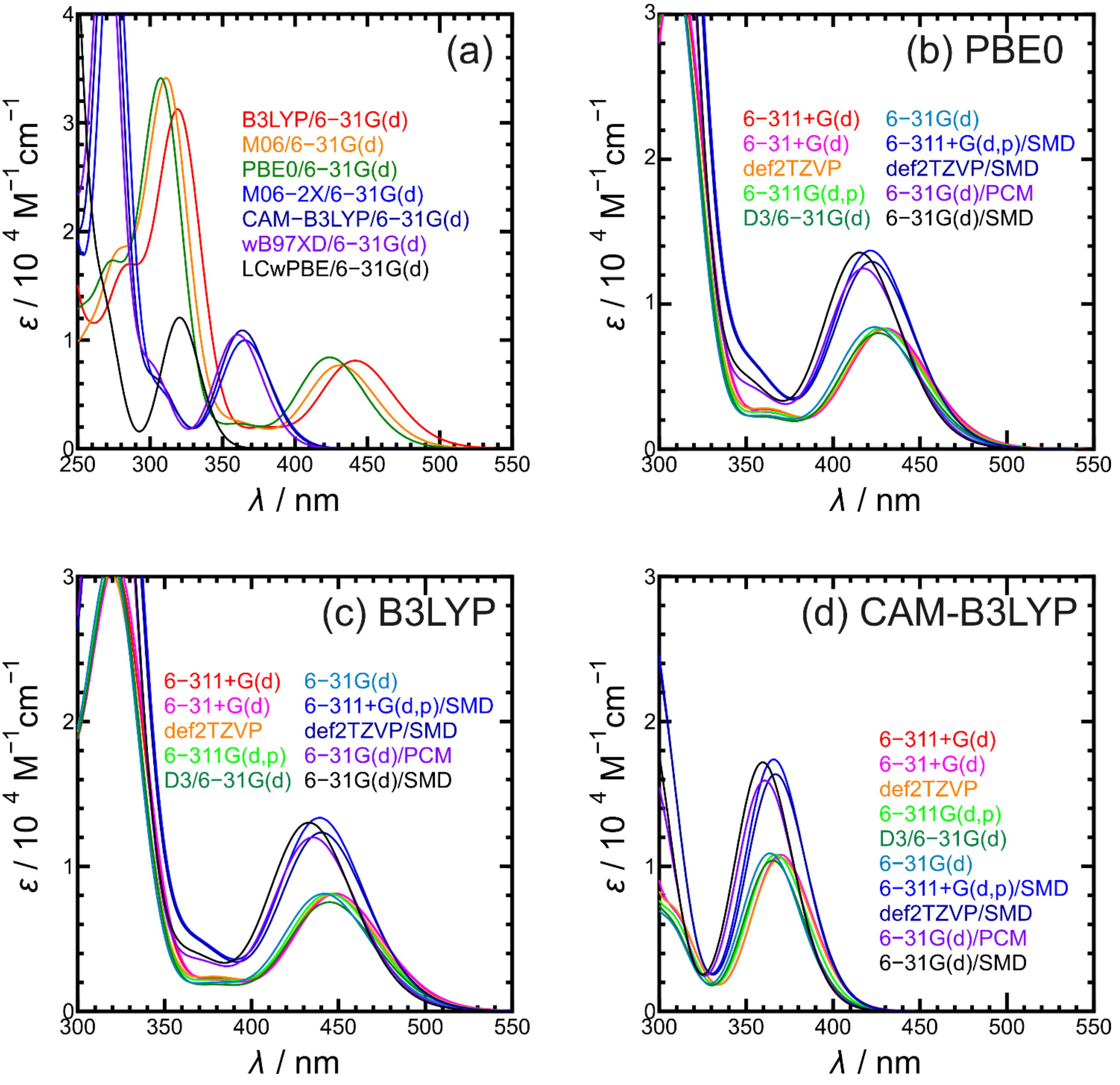

Figure S2. Comparison of calculated UV–vis absorption spectra of **TA[7]H**. (a) Effect of different density functionals (B3LYP, M06, PBE0, M06-2X, CAM-B3LYP, ωB97XD, LC-ωPBE) using the 6-31G(d) basis set. Basis set dependence, dispersion correction (Grimme's D3), and solvent effects for PBE0 (b), B3LYP (c), and CAM-B3LYP (d).

S3 TD-DFT/PBE0 Calculated Spectra of TA[*n*]H

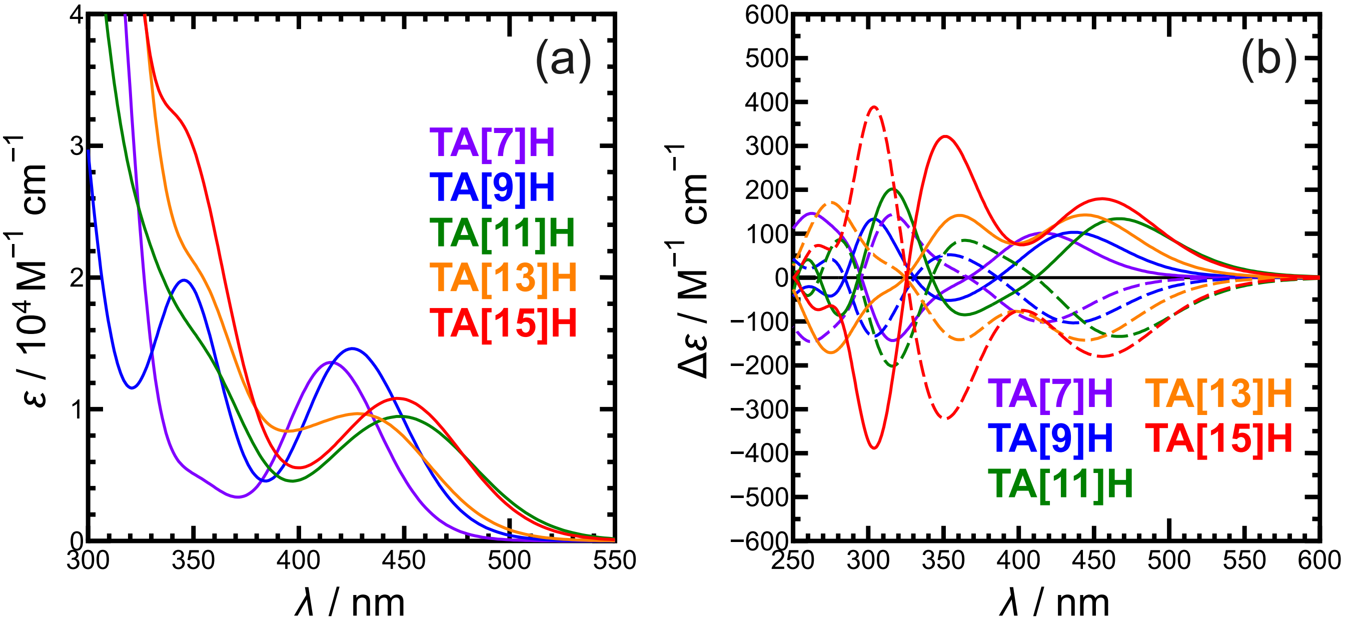

Figure S3. UV-Vis (a) and CD (b) spectra of **TA[*n*]H**, obtained using TD-DFT calculations at the PBE0/6-31G(d)/SMD level: Solid lines in (b) show the simulated CD spectra of the (P)-enantiomers, while dashed lines represent the corresponding inverted spectra. Both UV–Vis and CD spectra were generated with GaussSum using Gaussian broadening: UV–Vis with FWHM = 3000 cm^{-1} and CD with σ = 0.60 eV.

S4 HOMO and LUMO of the TA[17]–[21]H

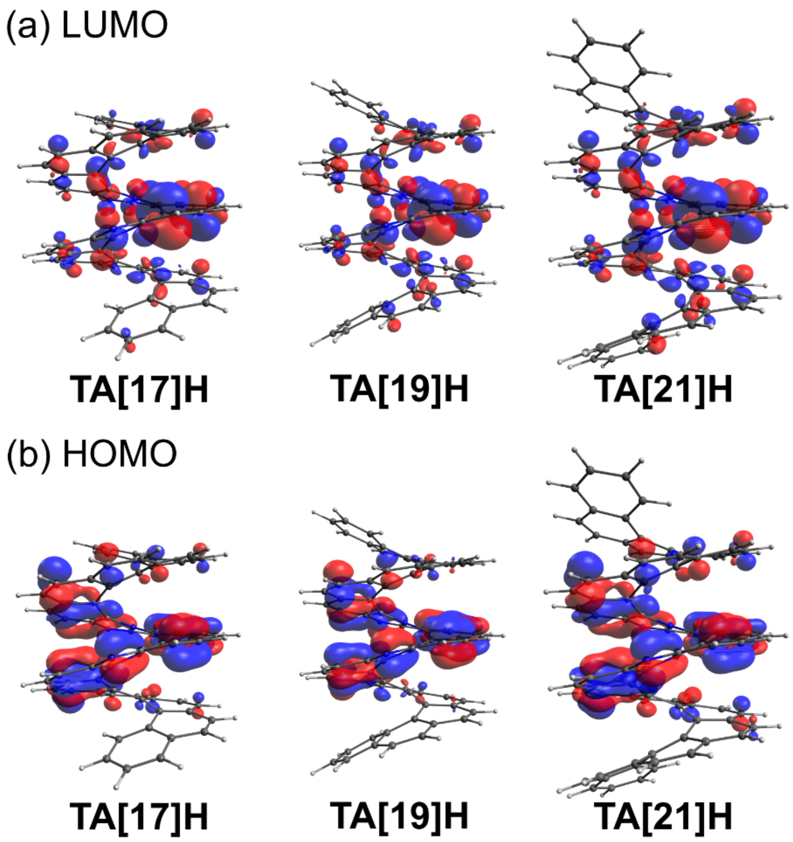

Figure S4. Side views of the HOMO and LUMO of the **TA[17]–[21]H** molecules, calculated at the PBE0/6-31G(d)/SMD level with an isosurface value set to 0.03.

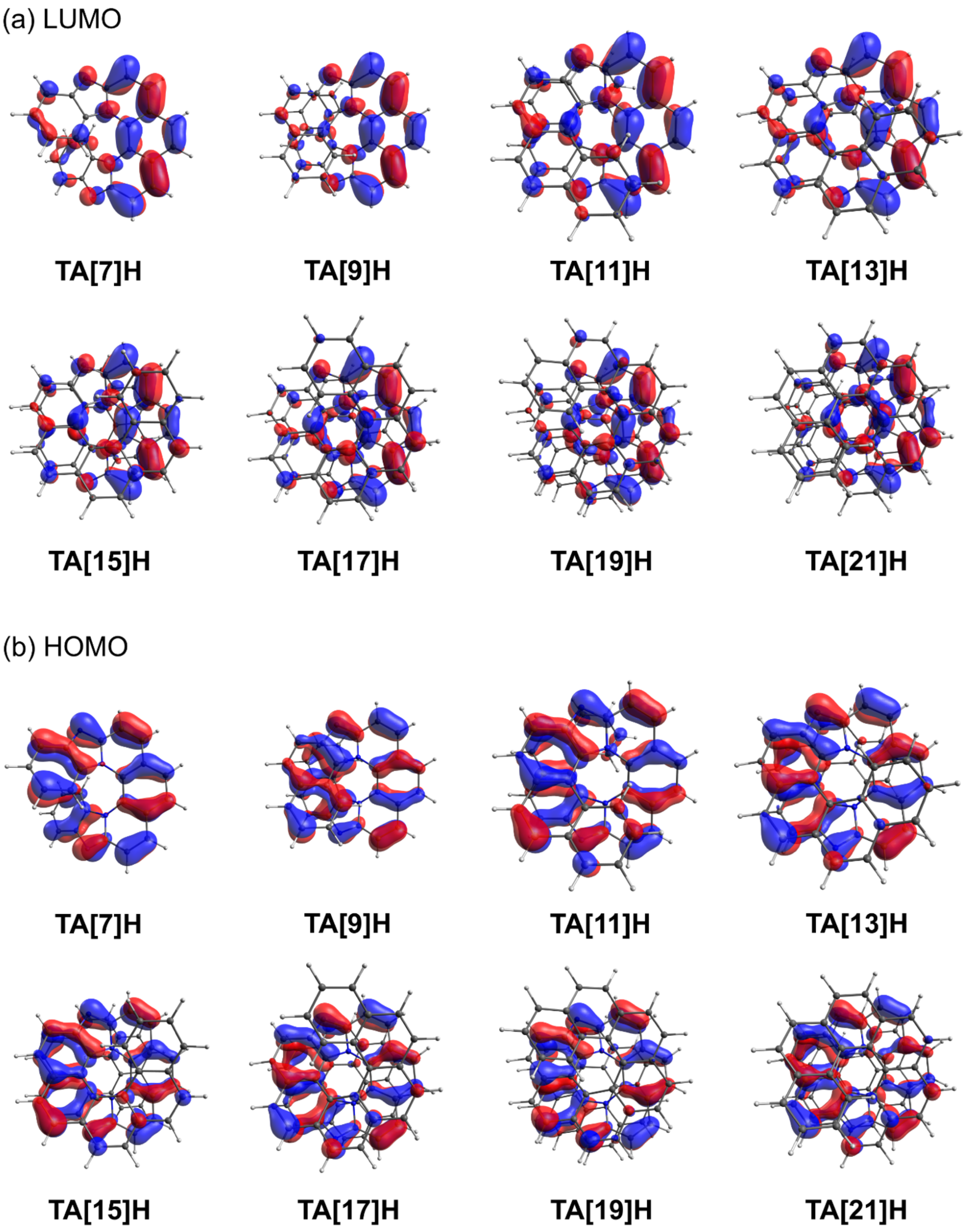

Figure S5. Top views of the HOMO and LUMO of the **TA[7]–[21]H** molecules, calculated at the PBE0/6-31G(d)/SMD level with an isosurface value set to 0.03.

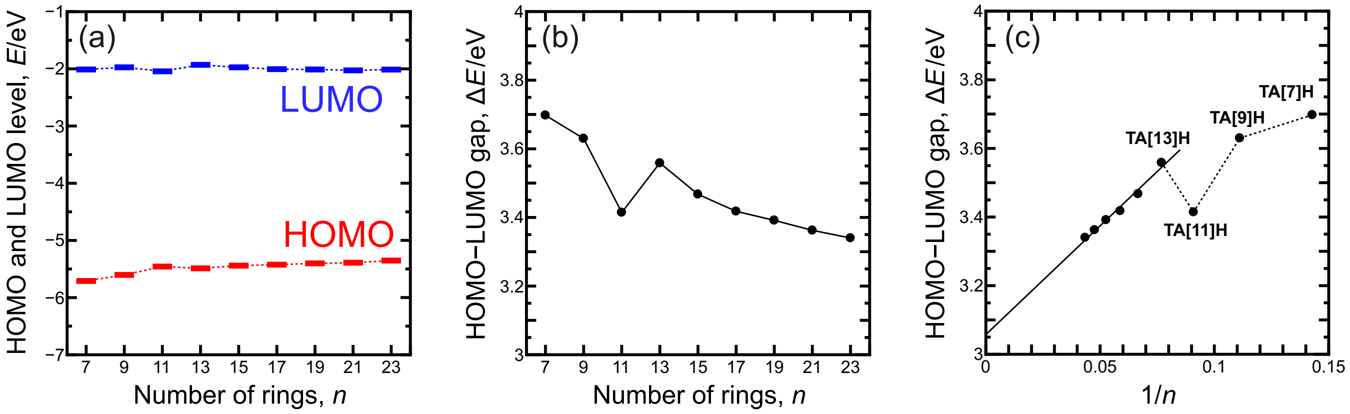

Figure S6. Calculated at the PBE0/6-31G(d)/SMD level: HOMO and LUMO energy levels (a) and the HOMO–LUMO gap of **TA[*n*]H** plotted against *n* (b) and 1/*n* (c). DFT calculations reveal that beyond **TA[13]H** the HOMO–LUMO gap decreases approximately linearly with 1/*n*, consistent with the trends observed for polyacenes and carbohelicenes. The solid line in (c) is a least-squares fit for $n \geq 13$.

S5 Orbital Contributions to the S_0–S_1 Excitation of TA[*n*]H

Tables S2 and S3 summarize the squared orbital contributions (≥ 1%) to the S_0–S_1 transition of **TA[*n*]H**, obtained using TD-DFT at the PBE0/6-31G(d)/SMD level. Table S1 is based on the ground-state (S_0)-optimized geometry, while Table S2 corresponds to the S_1-optimized geometry. In all cases, the S_0–S_1 excitation is dominated by the HOMO→LUMO π–π* transition. Additional calculations with B3LYP and CAM-B3LYP functionals confirmed the same qualitative picture. To further test the robustness of this result, additional TD-DFT calculations were performed using different exchange–correlation functionals (B3LYP and CAM-B3LYP) and larger basis sets. These calculations consistently yielded the same qualitative picture, namely a nearly pure HOMO→LUMO π–π* excitation. For completeness, the molecular orbital coefficients of the S_0–S_1 excitation for all **TA[*n*]H** systems, across all tested functionals and basis sets, are provided in a text file within the Supporting Information zip archive.

Table S2. Squared orbital contributions (≥ 1%) to the S_0–S_1 transition in **TA[*n*]H**, obtained from TD-DFT (PBE0/6-31G(d)/SMD) calculations at the S_0-optimized geometry. Rotational strengths were taken from the Gaussian TD-DFT output (rotational strength in the dipole length representation).

	Contributing Orbitals	Contribution (%)	Oscillator Strength	Excitation Energy (nm)	Rotational strengths (10^{-40}·erg·esu·cm/Gauss)
**TA [7]H**	HOMO → LUMO	98.3	0.1861	415.24	420.6
**TA [9]H**	HOMO → LUMO	97.9	0.1087	431.30	594.5
**TA[11]H**	HOMO → LUMO	98.5	0.0973	460.91	696.0
**TA[13]H**	HOMO → LUMO	97.3	0.0904	441.90	677.4
	HOMO−1 → LUMO+2	1.0			
**TA[15]H**	HOMO → LUMO	97.9	0.1184	454.20	834.3
	HOMO−1 → LUMO+2	1.0			
**TA[17]H**	HOMO → LUMO	97.5	0.1065	464.99	822.7
**TA[19]H**	HOMO → LUMO	96.4	0.0782	468.81	657.3
**TA[21]H**	HOMO → LUMO	95.4	0.1138	474.41	901.5
	HOMO−1 → LUMO+3	1.3			
	HOMO−1 → LUMO+1	1.2			

Table S3. Squared orbital contributions (≥ 1%) to the S_0–S_1 transition in **TA[*n*]H**, obtained from TD-DFT (PBE0/6-31G(d)/SMD) calculations at the S_1-optimized geometry. Rotational strengths were taken from the Gaussian TD-DFT output (rotational strength in the dipole length representation).

	Contributing Orbitals	Contribution (%)	Oscillator Strength	Excitation Energy (nm)	Rotational strengths (10^{-40}·erg·esu·cm/Gauss)
**TA[7]H**	HOMO → LUMO	99.1	0.2014	500.23	427.4
**TA[9]H**	HOMO → LUMO	98.7	0.1347	511.38	638.7
**TA[11]H**	HOMO → LUMO	99.4	0.0905	589.71	694.6
**TA[13]H**	HOMO → LUMO	99.0	0.0686	590.09	559.2
**TA[15]H**	HOMO → LUMO	99.1	0.0663	613.77	586.8
**TA[17]H**	HOMO → LUMO	99.0	0.0539	632.15	523.7
**TA[19]H**	HOMO → LUMO	98.8	0.0420	638.65	430.2
**TA[21]H**	HOMO → LUMO	98.9	0.0441	644.95	462.3

S6 Transition Dipole Orientations and Chiroptical Response

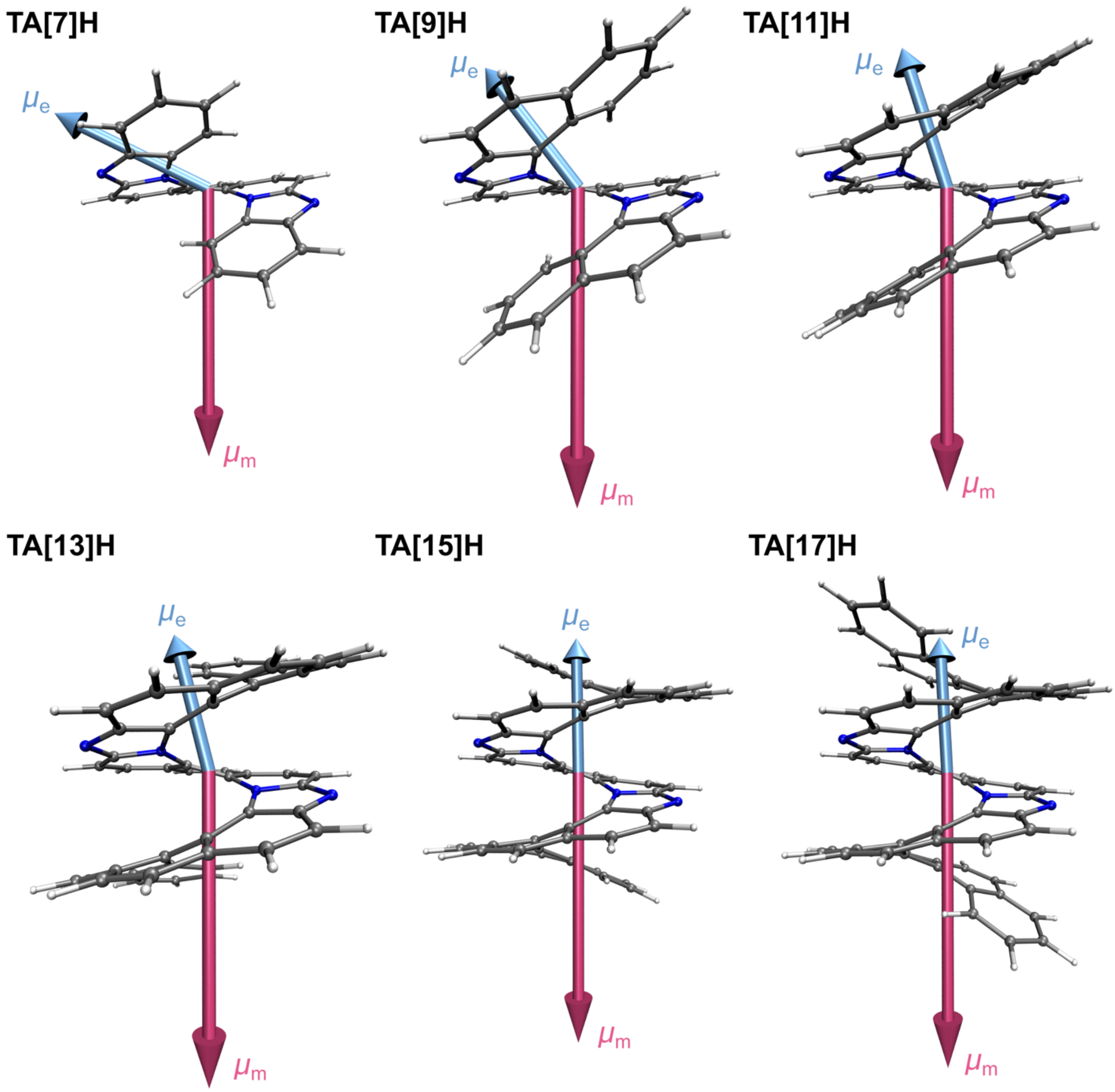

Figure S7. Visualization of the electric (μ_e, light blue arrow) and magnetic (μ_m, pink arrow) transition dipole moment vectors for the absorption of **TA[*n*]H**. Calculations were performed at the TD-DFT level (PBE0/6-31G(d)/SMD) based on the S_0-optimized structure and visualized using VMD [S7].

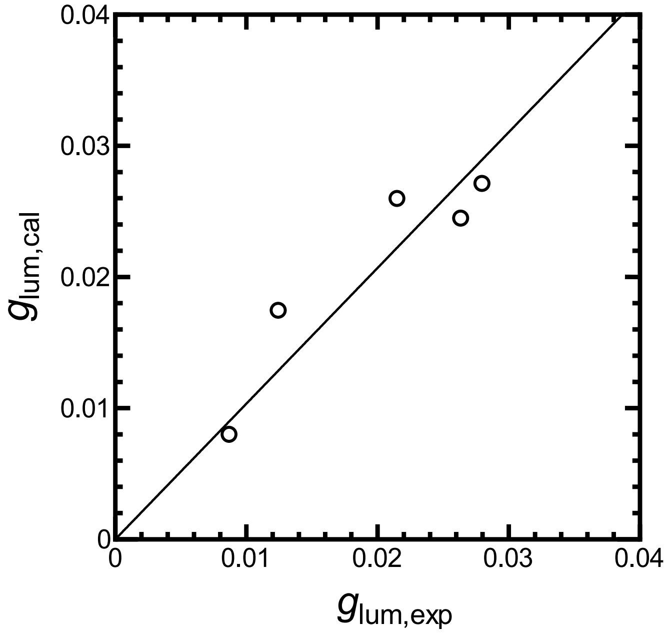

Figure S8. Correlation of calculated ($g_{\text{lum,cal}}$) vs. experimental ($g_{\text{lum,exp}}$) fluorescence g values. The calculated values were obtained using TD-DFT at the PBE0/6-31G(d)/SMD level of theory. The red line is the regression constrained to pass through the origin ($g_{\text{lum,cal}} = 1.03 \times g_{\text{lum,exp}}$). The root-mean-square error is 0.00306. Because the regression is constrained to the origin coefficient of determination was calculated as $R^2 = 1 - \text{SSE}/\sum(y_i - \bar{y})^2$, where SSE is the sum of squared errors, giving $R^2 = 0.980$.

Table S4. Experimental g_{lum} values and TD-DFT calculated g_{lum} values (all including solvent effects via the SMD model). For calculated entries, the values are shown as $g_{\text{lum,cal}}$ ($g_{\text{lum,cal}}/g_{\text{lum,exp}}$).

	**TA[7]H**	**TA[9]H**	**TA[11]H**	**TA[13]H**	**TA[15]H**
$g_{\text{lum, exp}}$	0.0087	0.012	0.026	0.022	0.028
$g_{\text{lum,cal}}$	0.0080	0.017	0.025	0.026	0.027
(PBE0/6-31G(d))	(0.91)	(1.40)	(0.93)	(1.21)	(0.97)
$g_{\text{lum,cal}}$	0.0079	0.017	0.022	0.024	0.025
(B3LYP/6-31G(d))	(0.91)	(1.36)	(0.82)	(1.10)	(0.89)
$g_{\text{lum,cal}}$	0.0078	0.017	0.026	0.027	0.030
(CAM-B3LYP/6-31G(d))	(0.90)	(1.38)	(0.98)	(1.25)	(1.06)
$g_{\text{lum,cal}}$	0.0082	0.018	0.027	0.029	0.030
(PBE0/def2-TZVP)	(0.94)	(1.50)	(1.03)	(1.34)	(1.06)
$g_{\text{lum,cal}}$	0.0078	0.018	0.027	0.028	0.029
(PBE0/6-311+G(d,p))	(0.90)	(1.46)	(1.02)	(1.32)	(1.05)
$g_{\text{lum,cal}}$	0.0083	0.019	0.031	0.032	0.034
(CAM-B3LYP/def2-TZVP)	(0.95)	(1.56)	(1.16)	(1.47)	(1.21)

S7 NMR Calculations

(a) TA[7]H

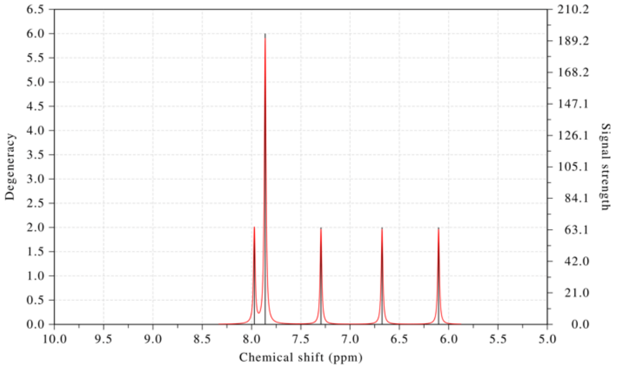

(b) TA[9]H

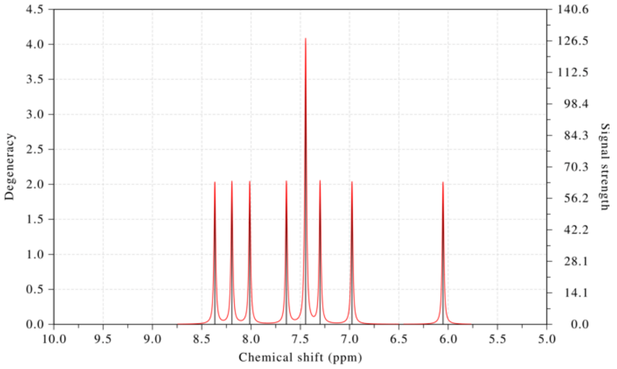

(c) TA[11]H

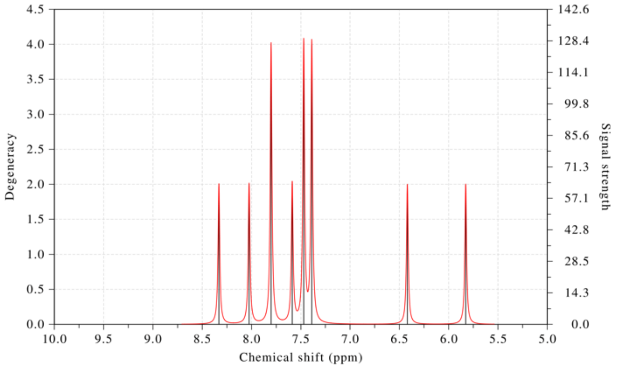

(d) TA[13]H

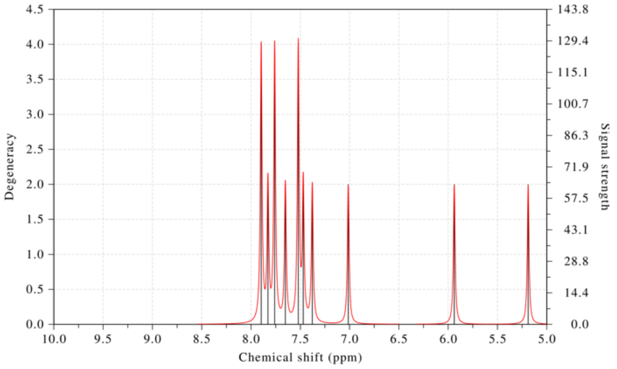

(e) TA[15]H

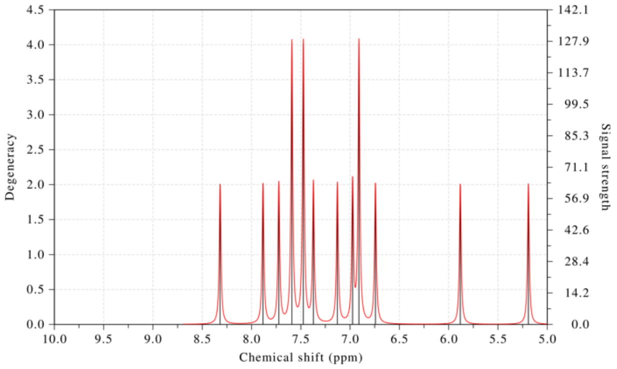

(f) TA[17]H

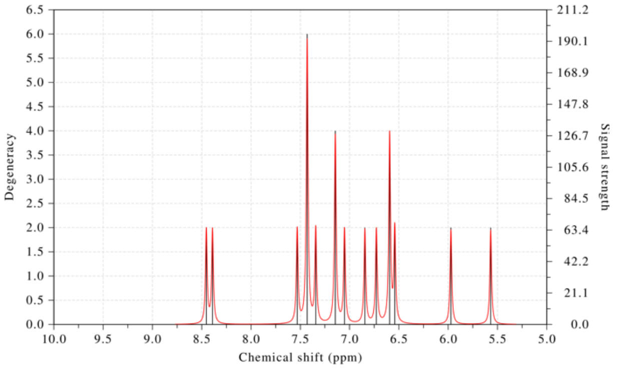

Figure S9. Chemical shifts of ^{1}H NMR for **TA[*n*]H** were calculated using the GIAO method at the B3LYP/6-31+G(d,p) level of theory with SMD solvent simulation, and the calculated values were corrected using an empirical scaling factor (slope: −1.0472; intercept: 31.6874) [S8]. These single point calculations were performed on the structures optimized at the B3LYP/6-31G(d) level of theory in the gas phase. These figures were generated using Multiwfn 3.7 [S9].

S8 HOMA Index Calculation

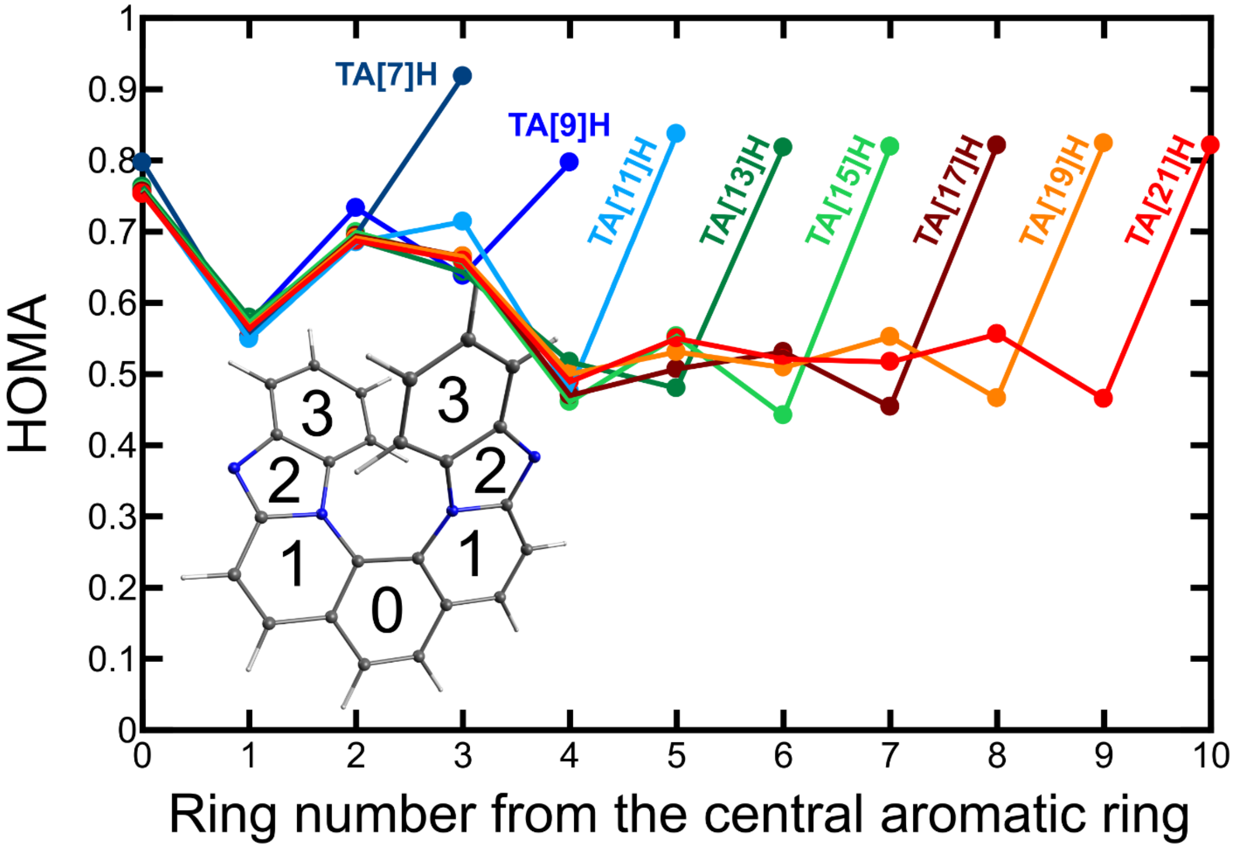

Figure S10. HOMA values for **TA[*n*]H** were calculated based on structures optimized at the B3LYP/6-31G(d) level of theory in the gas phase. The optimal bond lengths and normalization constants were set to 1.388 Å and 257.7 for C–C bonds, and 1.334 Å and 93.52 for C–N bonds. The analysis reveals that HOMA values around the aza-phenanthroline core remain nearly constant regardless of *n*, while the intermediate rings (about four rings away from the core) stabilize around ~0.5 with only minor fluctuations. In contrast, the terminal rings reach values as high as ~0.85, indicating pronounced aromaticity due to reduced structural strain. These trends demonstrate that aromaticity is preserved across the backbone, ensuring structural rigidity even upon molecular elongation.

S9 Robustness Check of μ_e, μ_m, and g Trends

To ensure that the main conclusions are not artifacts of the chosen computational setup, several complementary calculations were performed:

Alternative functionals (full optimization + TD-DFT): TA[*n*]H systems were re-optimized at the B3LYP/6-31G(d)/SMD and CAM-B3LYP/6-31G(d)/SMD levels, followed by TD-DFT calculations of transition dipole moments. In both cases, the qualitative n-dependence of θ and g values was identical to that obtained with PBE0/6-31G(d)/SMD as shown in Figure S11 and S12.

Larger basis sets (single-point TD-DFT): Using the PBE0/6-31G(d)/SMD optimized geometries, TD-DFT single-point calculations for **TA[*n*]H** ($n \leq 15$) were carried out at PBE0/def2-TZVP, PBE0/6-311+G(d,p), and CAM-B3LYP/def2-TZVP levels. The results consistently reproduced the same dipole orientation trends and g value enhancements as shown in Figure S13—15.

Gas-phase tests: For **TA[*n*]H** ($n \leq 15$), calculations in vacuo (i.e., without any solvation model) were carried out. The qualitative trends in g, θ, $|\mu_e|$, and $|\mu_m|$ were essentially the same as in solution, confirming that the mechanism does not depend on the implicit solvent treatment (Figure S16).

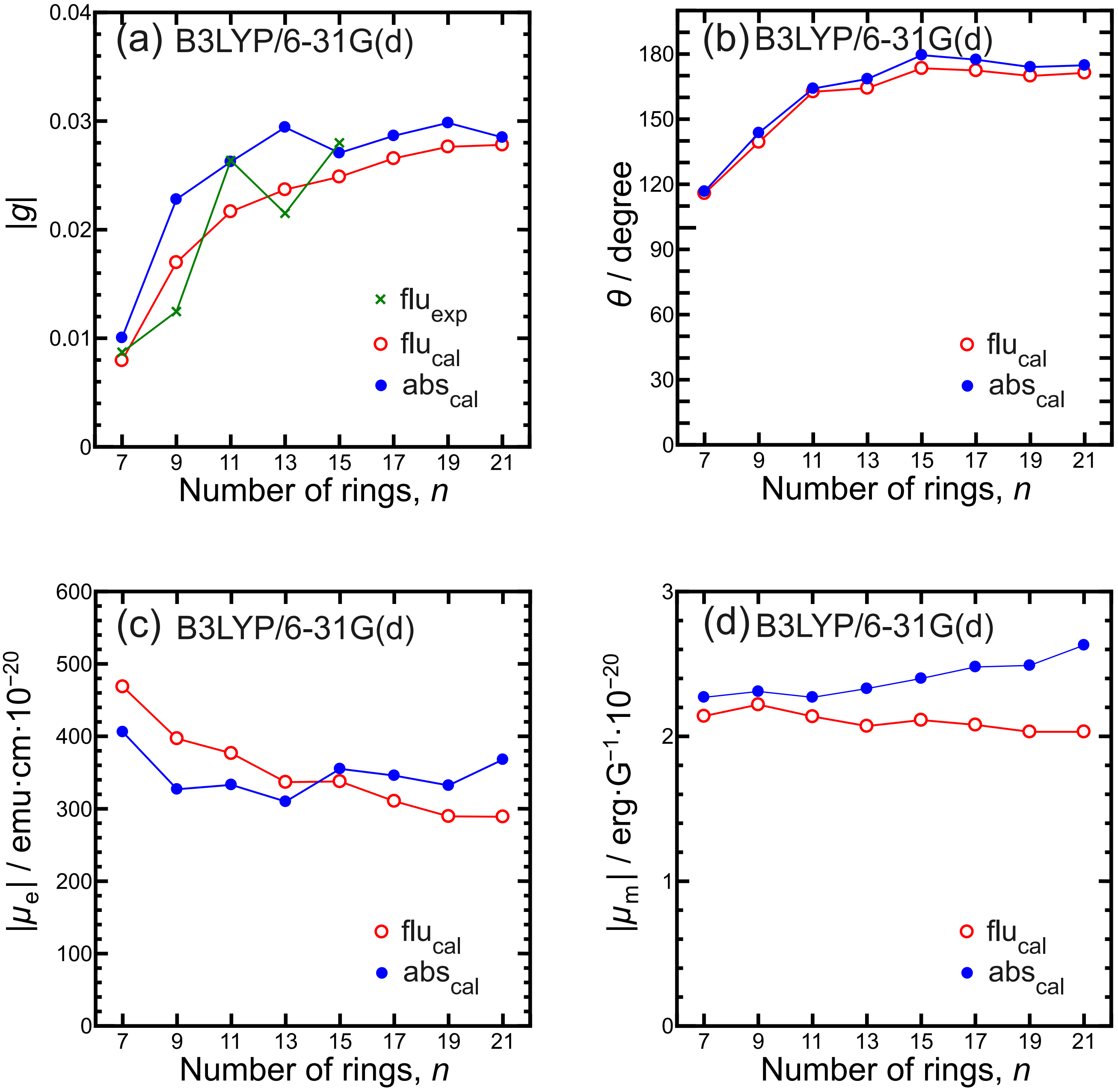

Figure S11. Chiroptical properties of **TA[*n*]H** molecules for different [*n*] values. (a) Calculated g values for absorption (blue closed circles) and fluorescence (red open circles), and experimental fluorescence *g* values (green crosses). (b) Angle θ between the electric (μ_e) and magnetic (μ_m) transition dipole moments. (c) Magnitude of the electric transition dipole moment $|\mu_e|$. (d) Magnitude of the magnetic transition dipole moment $|\mu_m|$. Calculations were performed at the TD-DFT level (B3LYP/6-31G(d)/SMD) based on S_0- and S_1-optimized structures.

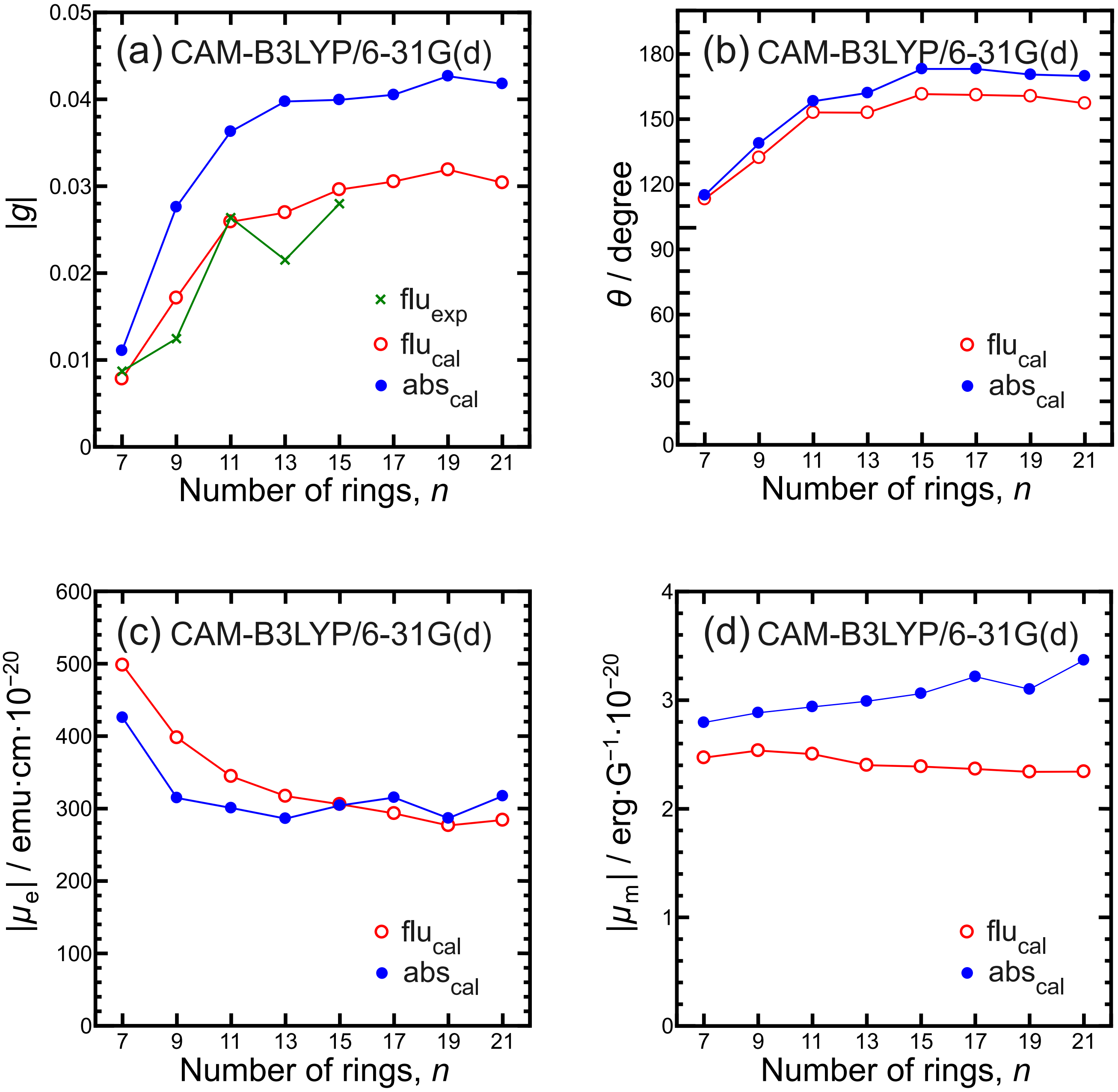

Figure S12. Chiroptical properties of **TA[*n*]H** molecules for different [*n*] values obtained from TD-DFT calculations at the CAM-B3LYP/6-31G(d)/SMD level based on S_0- and S_1-optimized structures. Panels (a–d) are defined as in Figure S11.

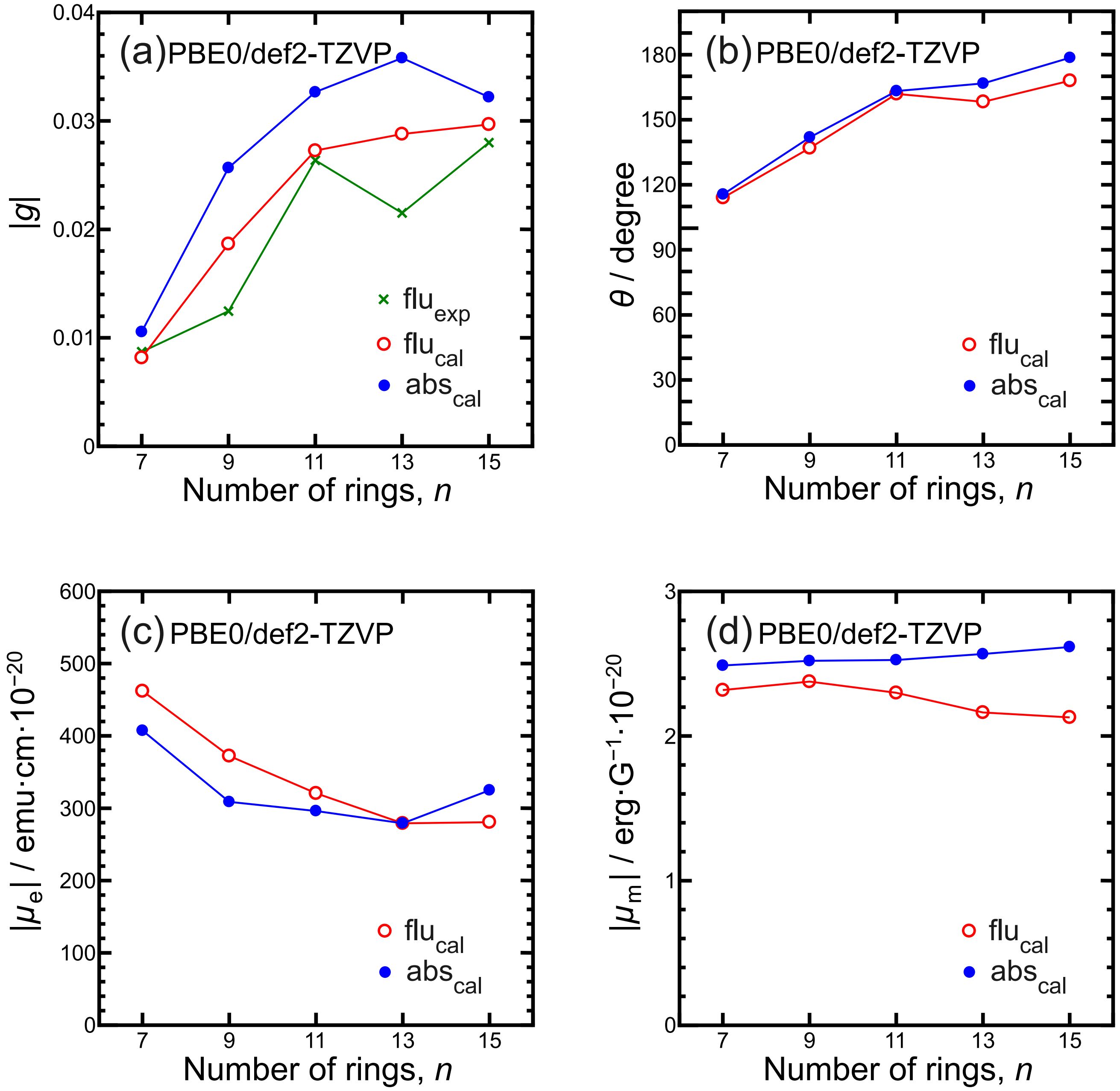

Figure S13. Chiroptical properties of **TA[*n*]H** molecules for different [*n*] values, obtained from single-point TD-DFT calculations at the PBE0/def2-TZVP/SMD level based on the PBE0/6-31G(d)/SMD optimized structures. Panels (a–d) are defined as in Figure S11.

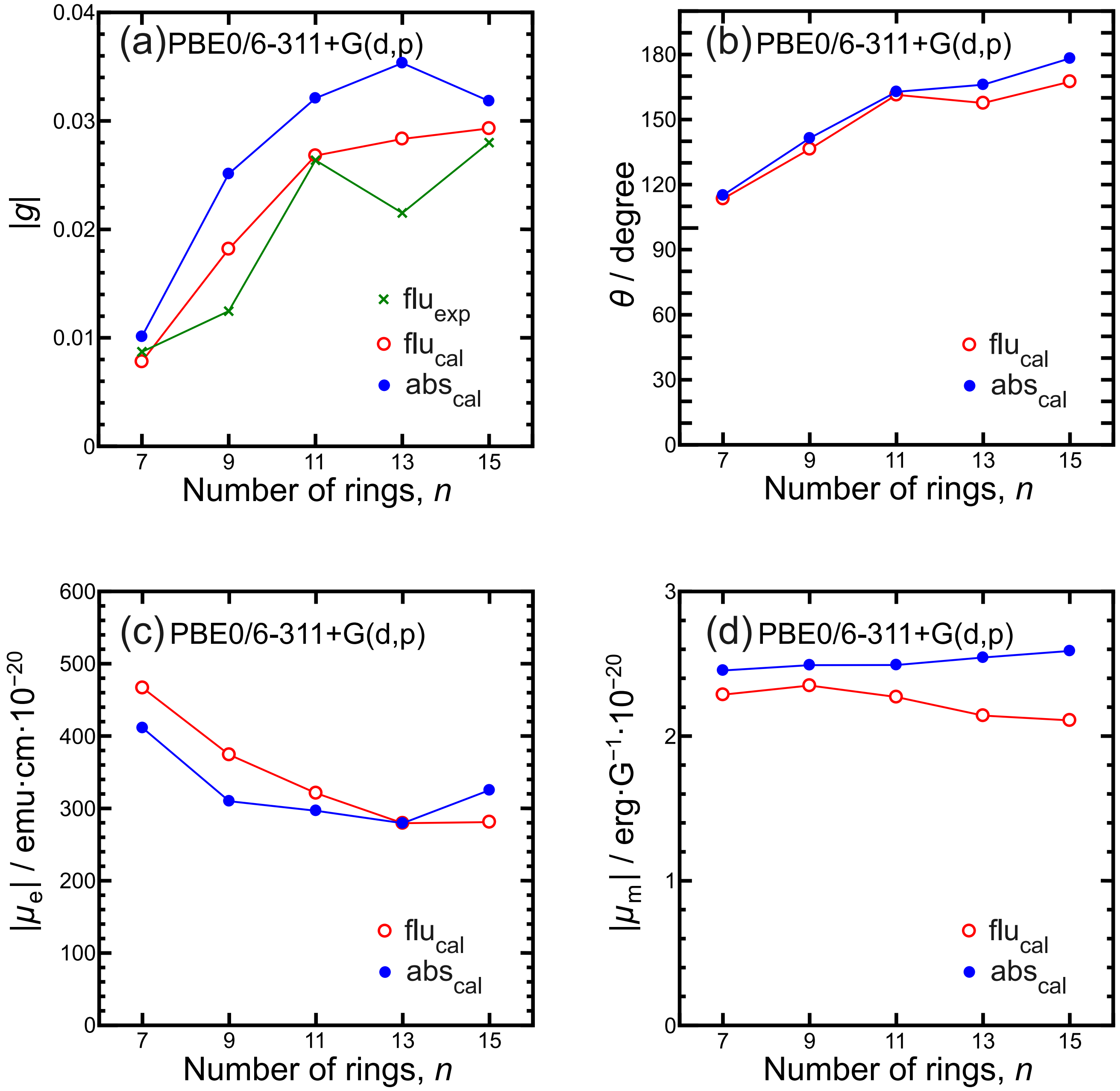

Figure S14. Chiroptical properties of **TA[*n*]H** molecules for different [*n*] values, obtained from single-point TD-DFT calculations at the PBE0/6-311+G(d, p) /SMD level based on the PBE0/6-31G(d)/SMD optimized structures. Panels (a–d) are defined as in Figure S11.

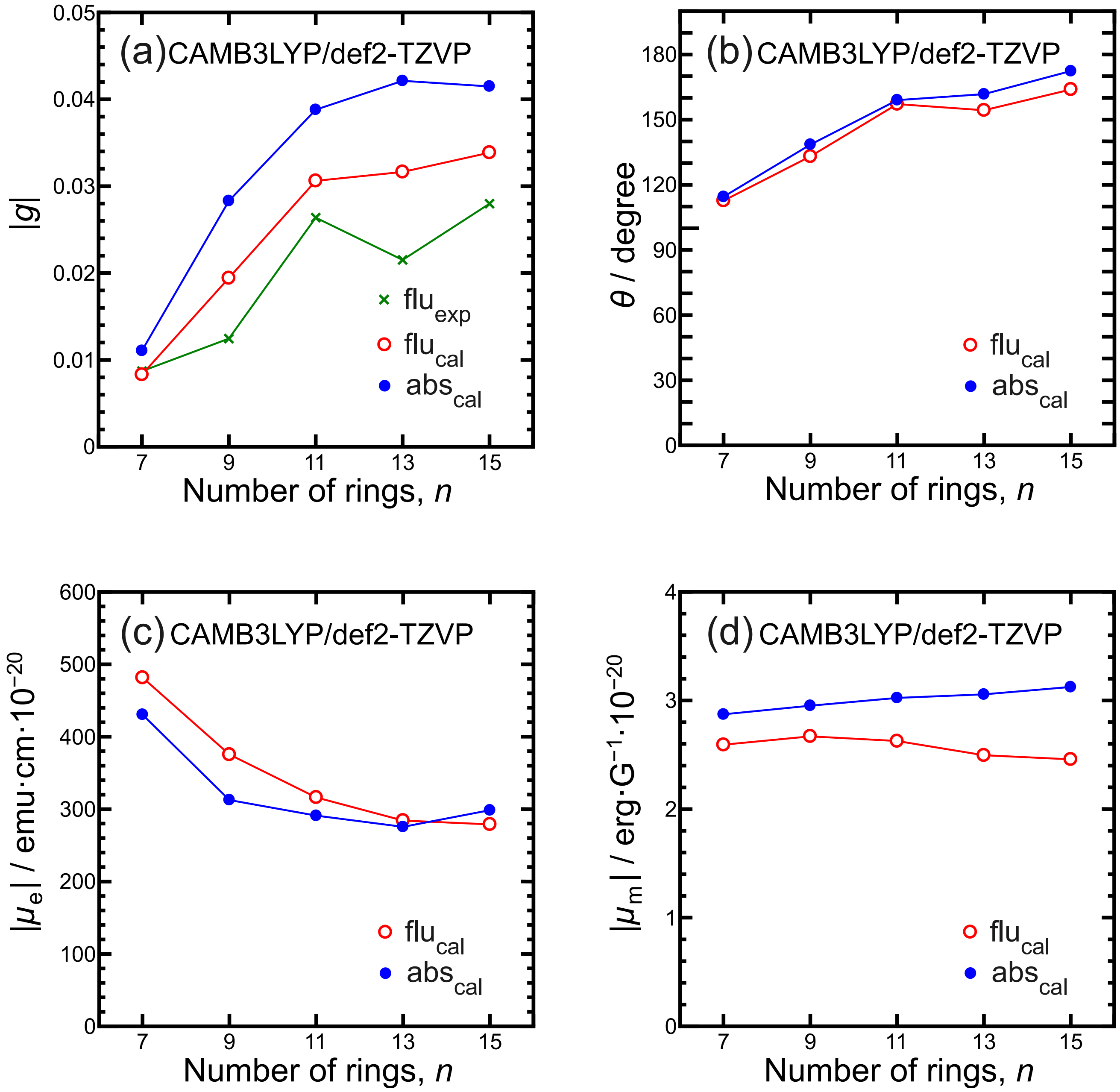

Figure S15. Chiroptical properties of **TA[*n*]H** molecules for different [*n*] values, obtained from single-point TD-DFT calculations at the CAM-B3LYP/def2-TZVP/SMD level based on the PBE0/6-31G(d)/SMD optimized structures. Panels (a–d) are defined as in Figure S11.

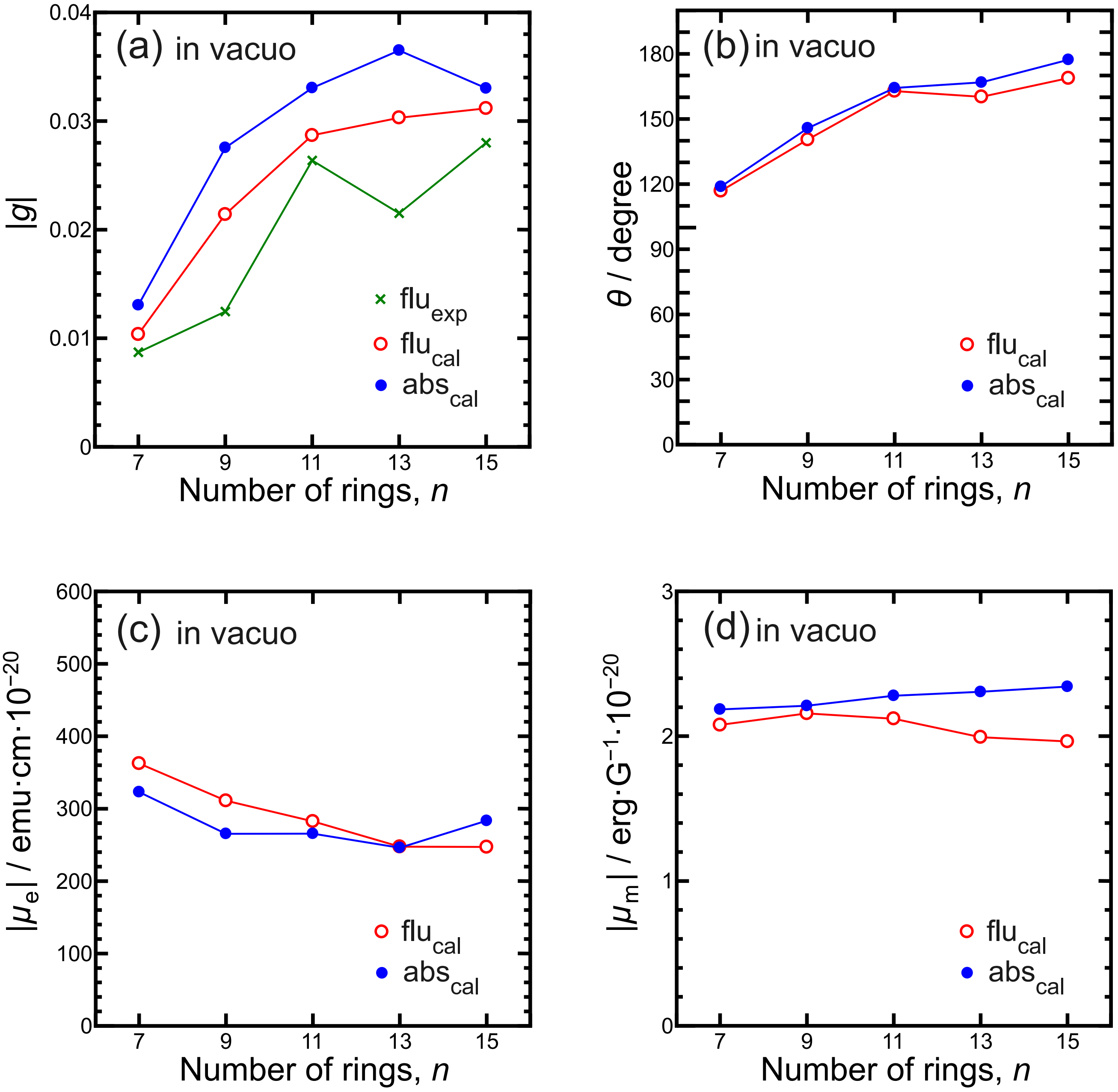

Figure S16. Chiroptical properties of **TA[*n*]H** molecules for different [*n*] values obtained from TD-DFT calculations at the PBE0/6-31G(d) level (in vacuo) based on S_0 and S_1 geometries optimized at the same level of theory. Panels (a–d) are defined as in Figure S11.

S10 Chiroptical Properties of Even-Membered TA[*n*]H

To obtain a broader understanding of the structure–property relationships in this series, we examined whether any odd–even dependence appears in the optical and chiroptical properties as a function of *n*. Because the **TA[*n*]H** series contains a phenanthroline unit at its center, even-membered systems (i.e., even *n*) necessarily possess different numbers of aromatic rings on both sides. We therefore denote these as **TA[*n*]H*ab***, where *a* and *b* represent the numbers of appended aromatic rings on each side. Based on DFT calculations, we compared the chiroptical properties of the odd-membered TA[*n*]H homologues with those of the minimally asymmetric even-membered systems (**TA[8]H12**, **TA[10]H23**, **TA[12]H34**, **TA[14]H45**, and **TA[16]H56**) . In this analysis, the *a* and *b* with $b = a + 1$ were used to minimize the influence of asymmetry originating from the different numbers of rings on each side. The results are summarized in Figure S17. In almost all cases, the even-membered systems fall within the interpolation of the odd–membered data points, indicating that the properties change continuously with respect to *n* and that no pronounced odd–even behavior emerges. It should be noted, however, that even-membered **TA[*n*]H*ab*** are inherently asymmetric, and the optical properties depend on the degree of ring-number asymmetry, as shown in Figure S18. Therefore, the scope of a strict odd–even comparison within this series is inherently limited.

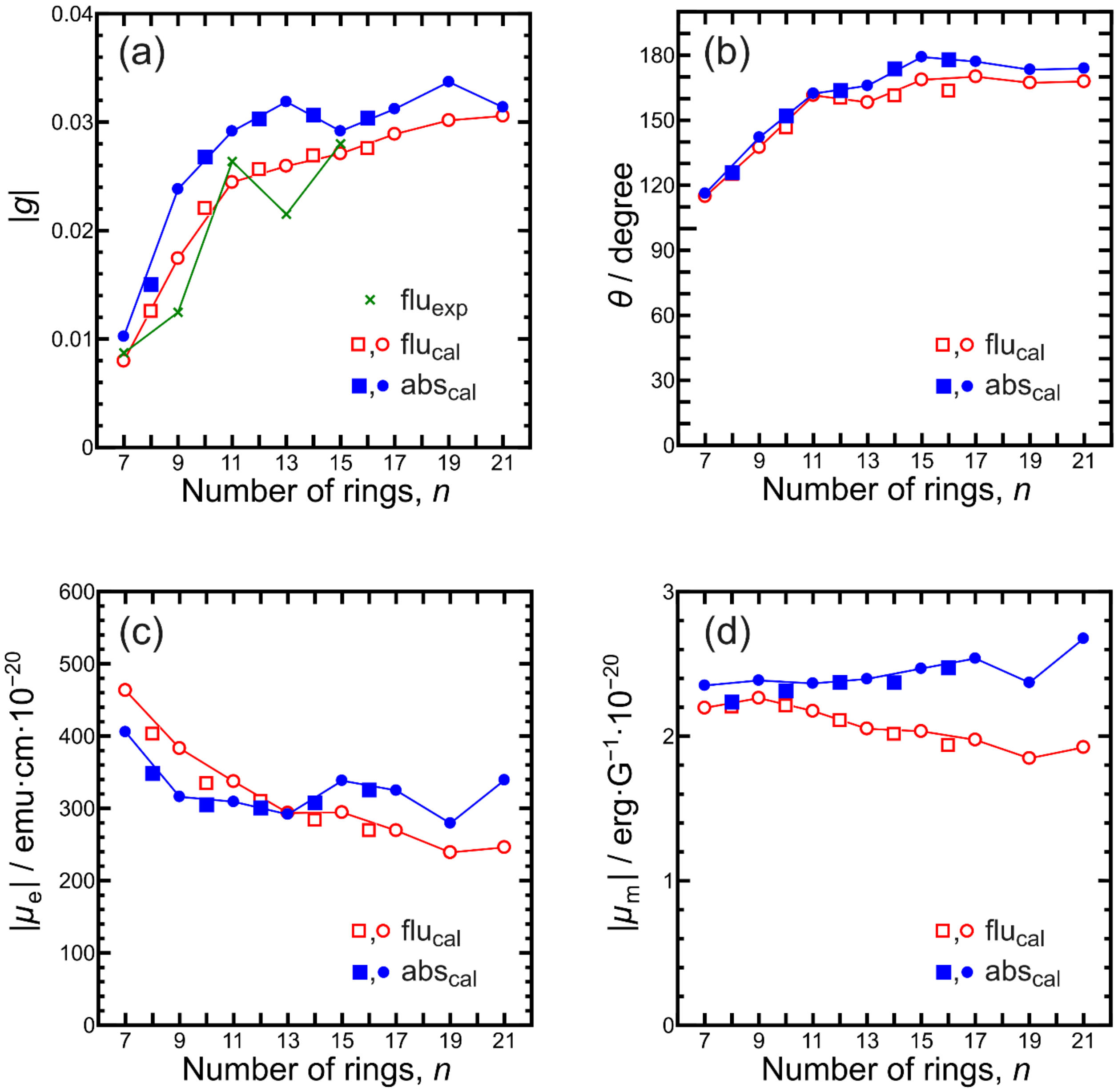

Figure S17. Chiroptical properties of **TA[*n*]H** molecules for different [*n*] values obtained from TD-DFT calculations at the PBE0/6-31G(d)/SMD level based on S_0- and S_1-optimized structures. Panels (a–d) are defined as in Figure S11. Odd-membered **TA[*n*]H** are plotted as closed blue circles (absorption) and open red circles (fluorescence), whereas even-membered systems are shown as closed blue squares and open red squares. For clarity of comparison, the odd-membered data points are connected with lines to provide a reference trend along the homologous series.

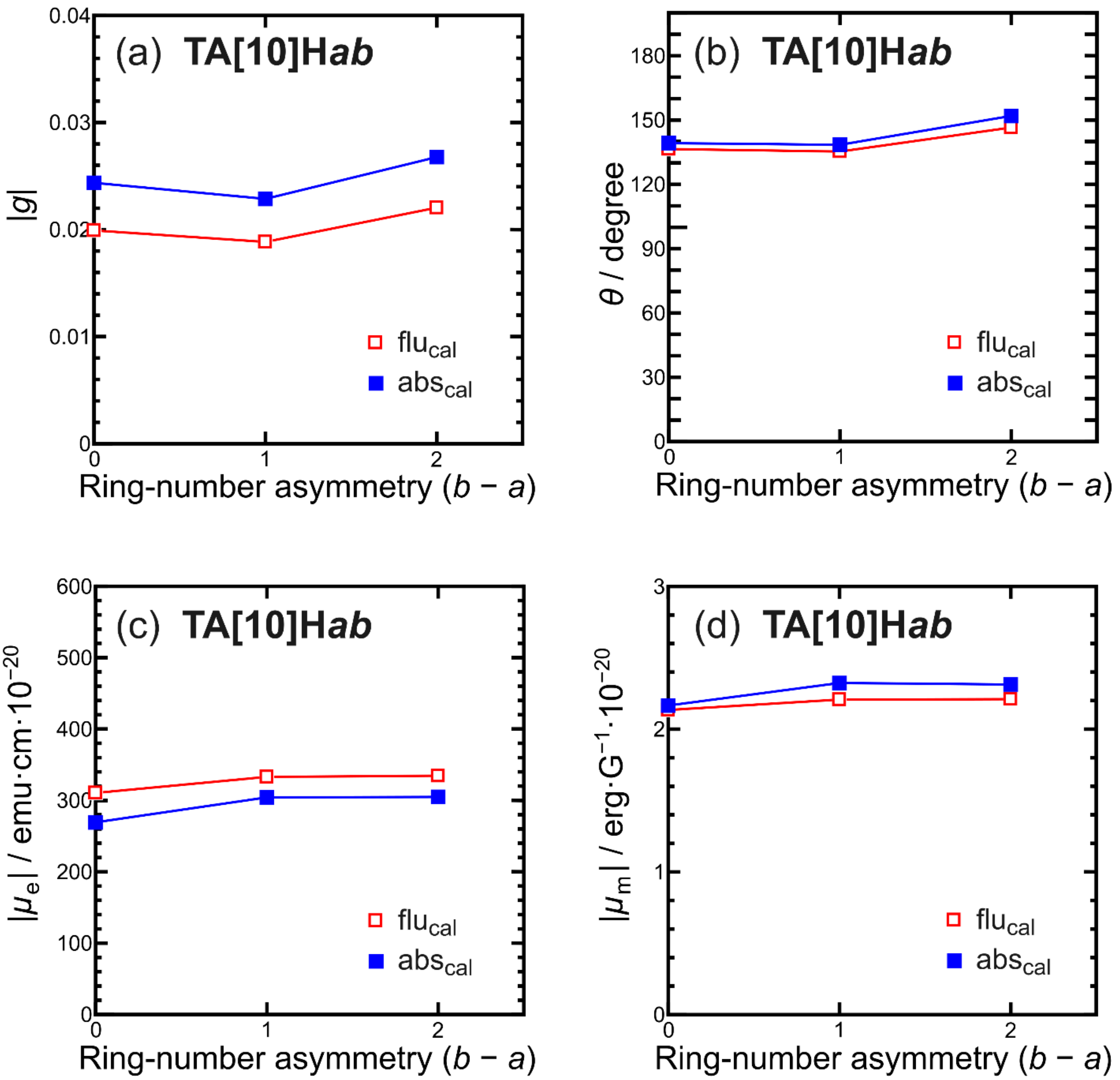

Figure S18. Chiroptical properties of **TA[10]H*ab*** as a function of ring-number asymmetry (*b* – *a*) obtained from TD-DFT calculations at the PBE0/6-31G(d)/SMD level based on S_0- and S_1-optimized structures. Panels (a–d) are defined as in Figure S11. All data points correspond to calculated structures with integer values of a and b (**TA[10]H05**, **TA[10]H14**, and **TA[10]H23**). The rightmost point (*b* – *a* = 2.5) corresponds to a hypothetical symmetric limit of **TA[10]H*ab***, shown only as a reference; no such structure exists experimentally because *a* and *b* must be integers.

C. References

(S1) R. A. Green, J. F. Hartwig, *Org. Lett.* **2014**, *16*, 4388-4391.

(S2) E. B. Prage, S.-C. Pawelzik, L. S. Busenlehner, K. Kim, R. Morgenstern, P.-J. Jakobsson, R. N. Armstrong, *Biochemistry* **2011**, *50*, 7684-7693.

(S3) Macé, A.; Hamrouni, K.; Gauthier, E. S.; Jean, M.; Vanthuyne, N.; Frédéric, L.; Pieters, G.; Caytan, E.; Roisnel, T.; Aloui, F.; Srebro-Hooper, M.; Carboni, B.; Berrée, F.; Crassous, J., Circularly Polarized Fluorescent Helicene-Boranils: Synthesis, Photophysical and Chiroptical Properties. *Chem. Eur. J.* **2021**, *27*, 7959-7967.

(S4) (a) B. Irziqat, A. Cebrat, M. Baljozović, K. Martin, M. Parschau, N. Avarvari, K.-H. Ernst, *Chem. Eur. J.* **2021**, *27*, 13523-13526. (b) M. Jakubec, T. Beránek, P. Jakubík, J. Sýkora, J. Žádný, V. Církva, J. Storch, *J. Org. Chem.* **2018**, *83*, 3607-3616.

(S5) Gaussian 16, Revision C.02, M. J. Frisch, G. W. Trucks, H. B. Schlegel, G. E. Scuseria, M. A. Robb, J. R. Cheeseman, G. Scalmani, V. Barone, G. A. Petersson, H. Nakatsuji, X. Li, M. Caricato, A. V. Marenich, J. Bloino, B. G. Janesko, R. Gomperts, B. Mennucci, H. P. Hratchian, J. V. Ortiz, A. F. Izmaylov, J. L. Sonnenberg, D. Williams-Young, F. Ding, F. Lipparini, F. Egidi, J. Goings, B. Peng, A. Petrone, T. Henderson, D. Ranasinghe, V. G. Zakrzewski, J. Gao, N. Rega, G. Zheng, W. Liang, M. Hada, M. Ehara, K. Toyota, R. Fukuda, J. Hasegawa, M. Ishida, T. Nakajima, Y. Honda, O. Kitao, H. Nakai, T. Vreven, K. Throssell, J. A. Montgomery, Jr., J. E. Peralta, F. Ogliaro, M. J. Bearpark, J. J. Heyd, E. N. Brothers, K. N. Kudin, V. N. Staroverov, T. A. Keith, R. Kobayashi, J. Normand, K. Raghavachari, A. P. Rendell, J. C. Burant, S. S. Iyengar, J. Tomasi, M. Cossi, J. M. Millam, M. Klene, C. Adamo, R. Cammi, J. W. Ochterski, R. L. Martin, K. Morokuma, O. Farkas, J. B. Foresman, and D. J. Fox, Gaussian, Inc., Wallingford CT, 2016.

(S6) N. M. O'boyle, A. L. Tenderholt, K. M. Langner, *J. Comp. Chem.* **2008**, 29, 839-845.

(S7) W. Humphrey, A. Dalke, K. Schulten, *J. Mol. Graphics* **1996**, *14*, 33-38.

(S8) M. W. Lodewyk, M. R. Siebert, D. J. Tantillo, *Chem. Rev.* **2012**, *112*, 1839-1862.

(S9) T. Lu, *J. Chem. Phys.* **2024**, *161*, 082503.

D. Copies of ^{1}H and ^{13}C NMR spectra of new compounds

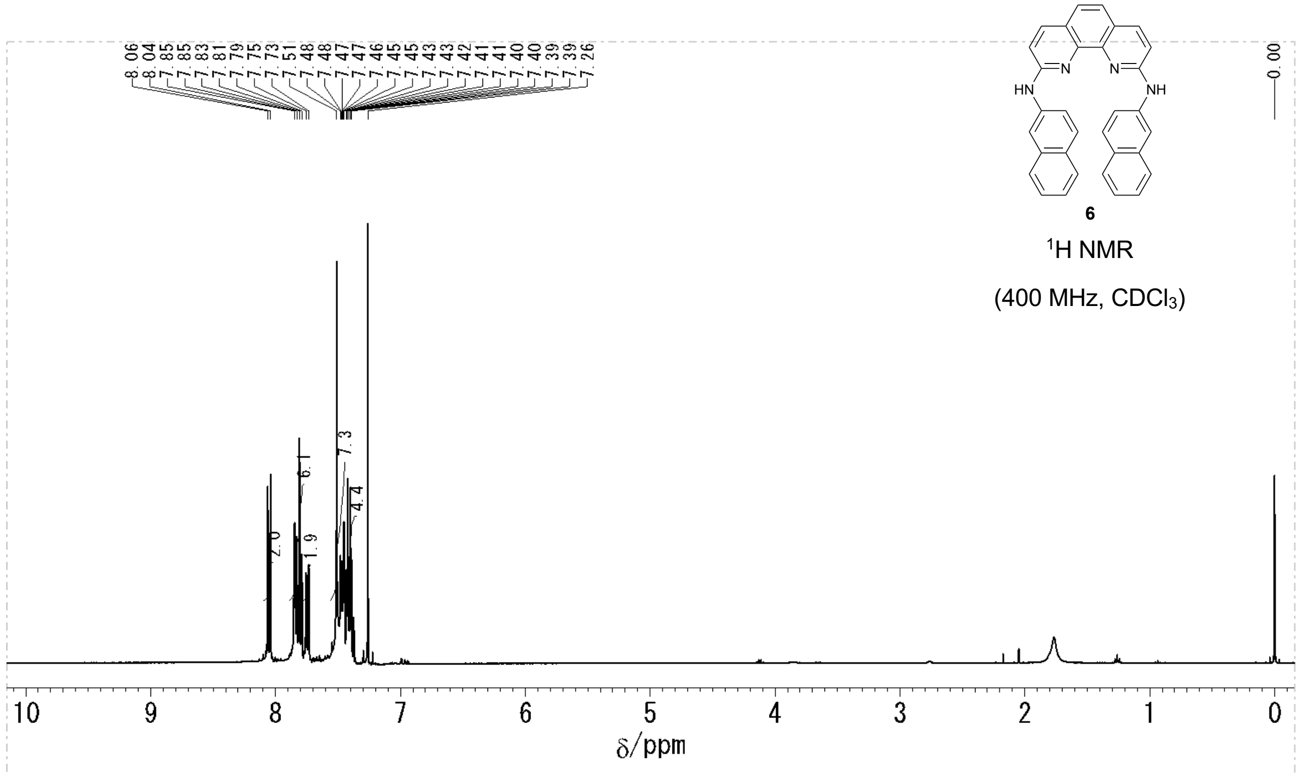

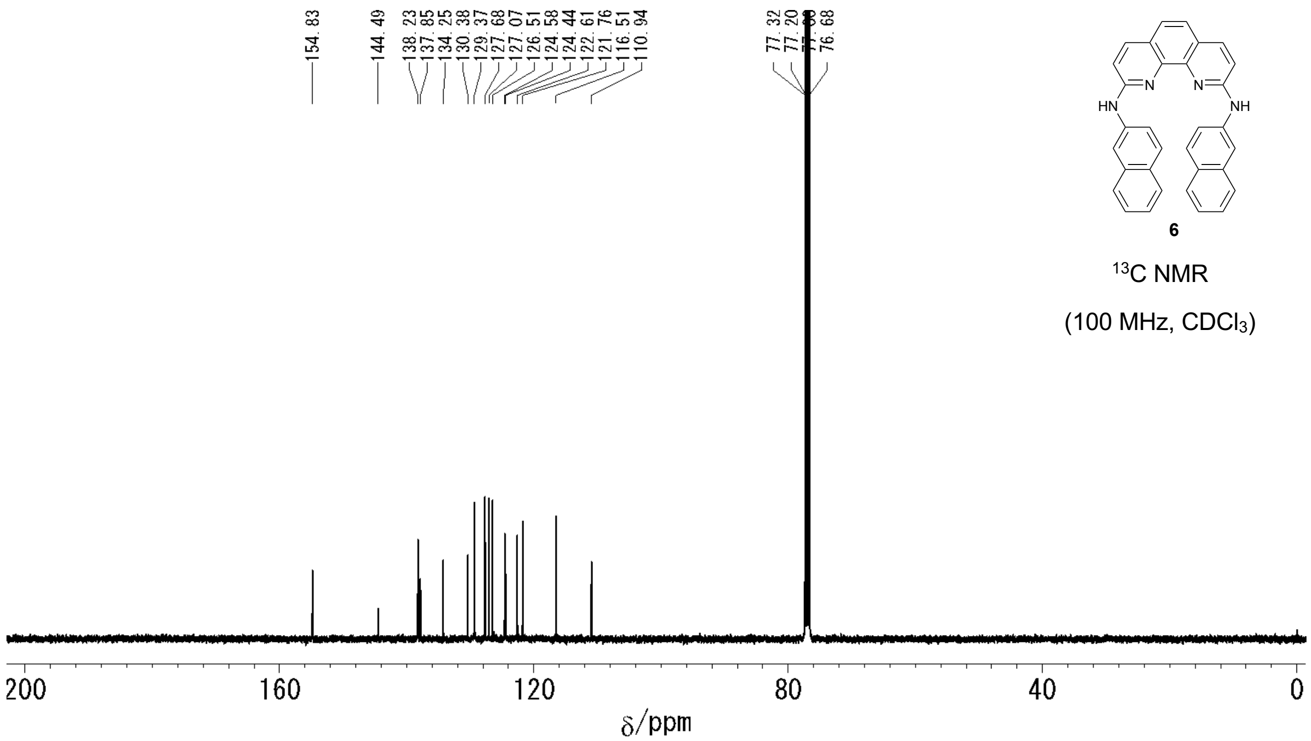

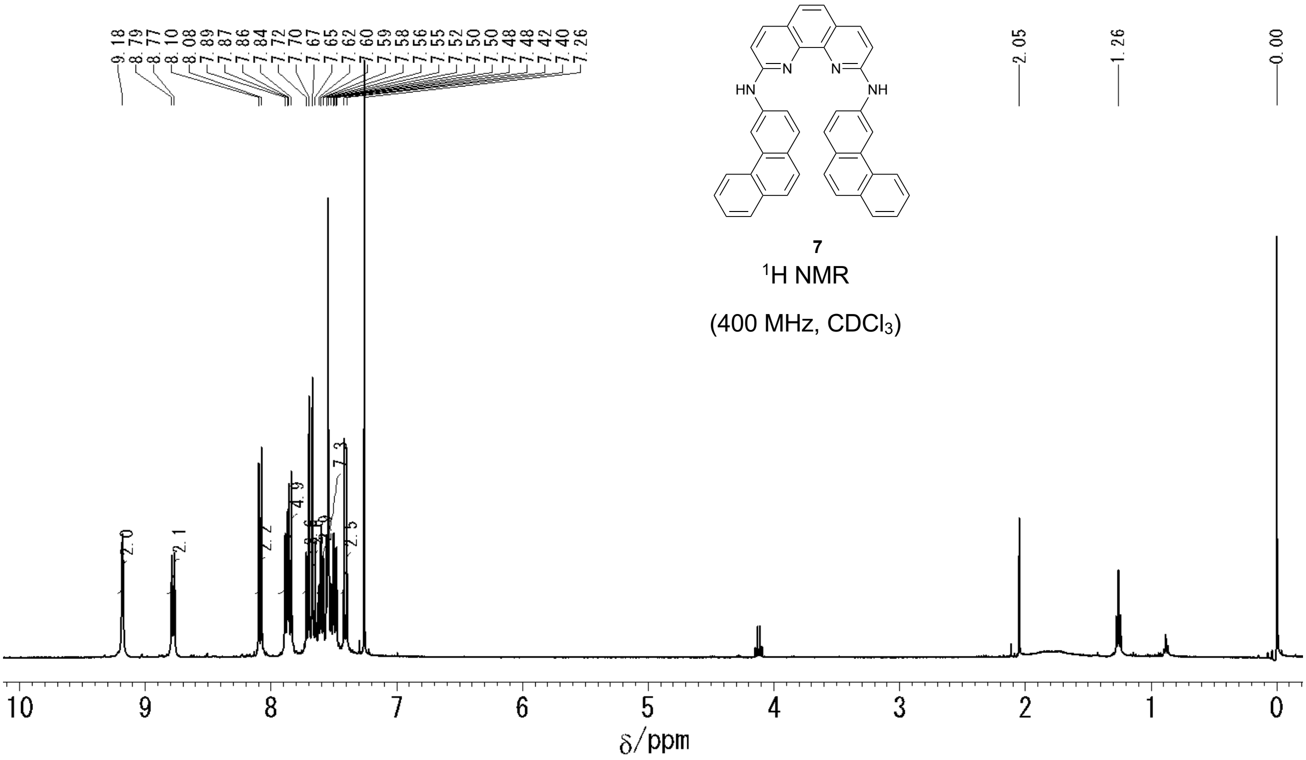

9.18
8.79
8.77
8.10
8.08
7.89
7.87
7.86
7.84
7.72
7.70
7.67
7.65
7.62
7.60
7.59
7.58
7.56
7.55
7.52
7.50
7.50
7.48
7.48
7.42
7.40
7.26
2.05
1.26
0.00
HN
NH
N
N
7
^{1}H NMR
(400 MHz, $CDCl_3$)
2.0
2.1
2.2
4.9
7.3
2.5
δ/ppm

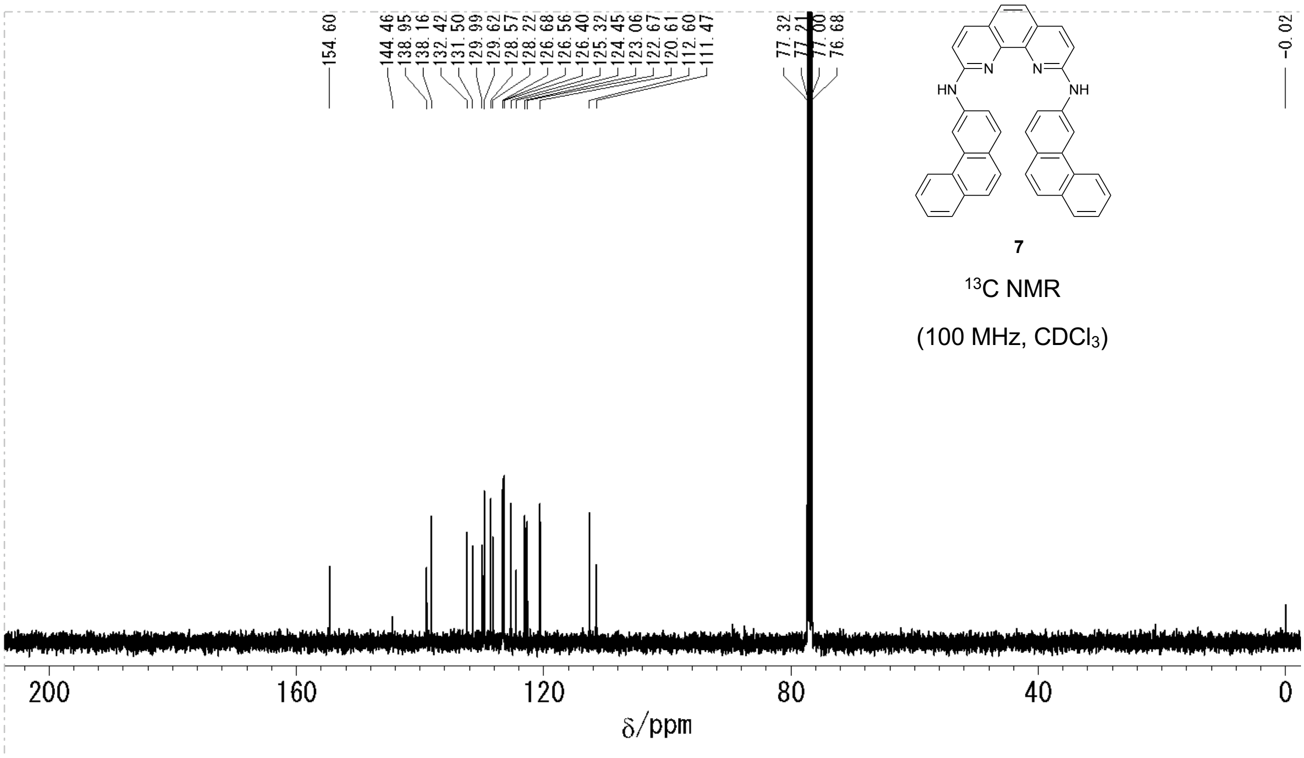

154.60
144.46
138.95
138.16
132.42
131.50
129.99
129.62
128.57
128.22
126.68
126.56
126.40
125.32
124.45
123.06
122.67
120.61
112.60
111.47
77.32
77.00
76.68
-0.02
HN
NH
N
N
7
^{13}C NMR
(100 MHz, $CDCl_3$)
δ/ppm

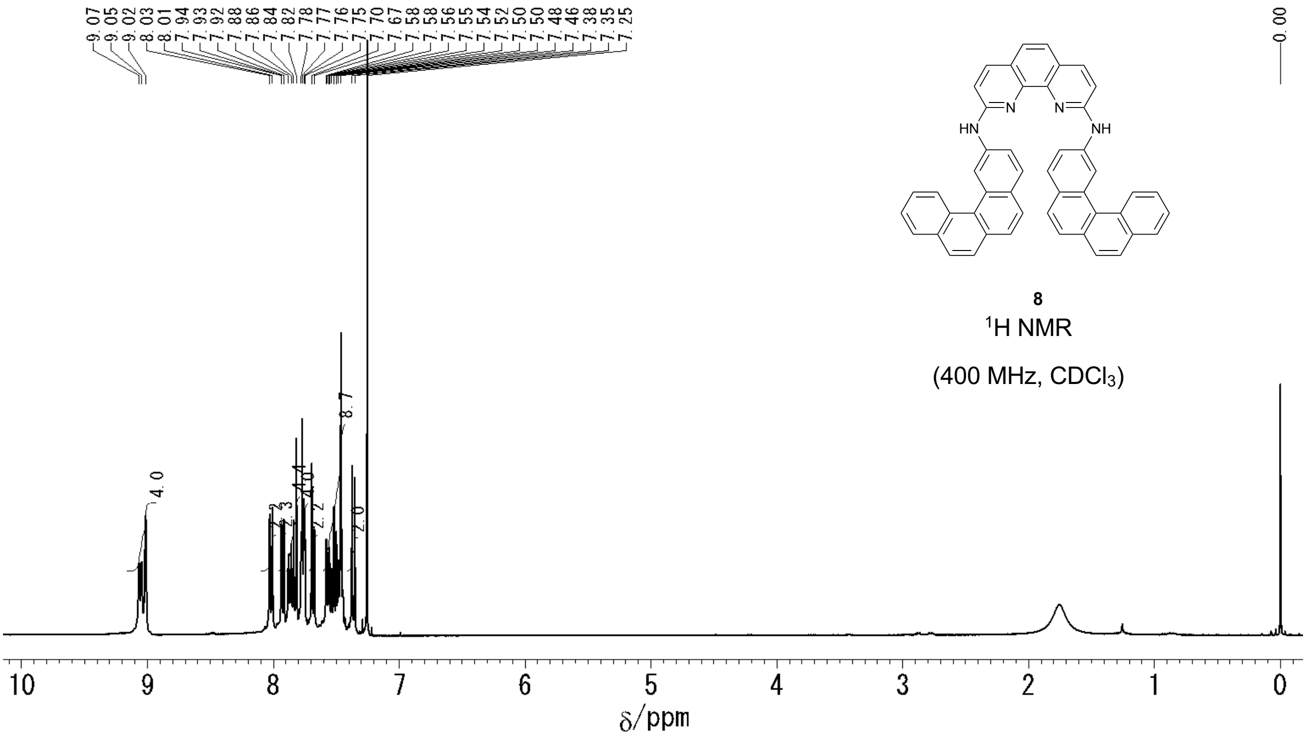
9.07 9.05 9.02 8.03 8.01 7.94 7.93 7.92 7.88 7.86 7.84 7.82 7.78 7.77 7.76 7.75 7.70 7.67 7.58 7.58 7.56 7.55 7.54 7.52 7.50 7.48 7.46 7.38 7.35 7.25
0.00
4.0
8.7
2.0
HN
NH
N
N
8
^{1}H NMR
(400 MHz, $CDCl_3$)
δ/ppm
10 9 8 7 6 5 4 3 2 1 0

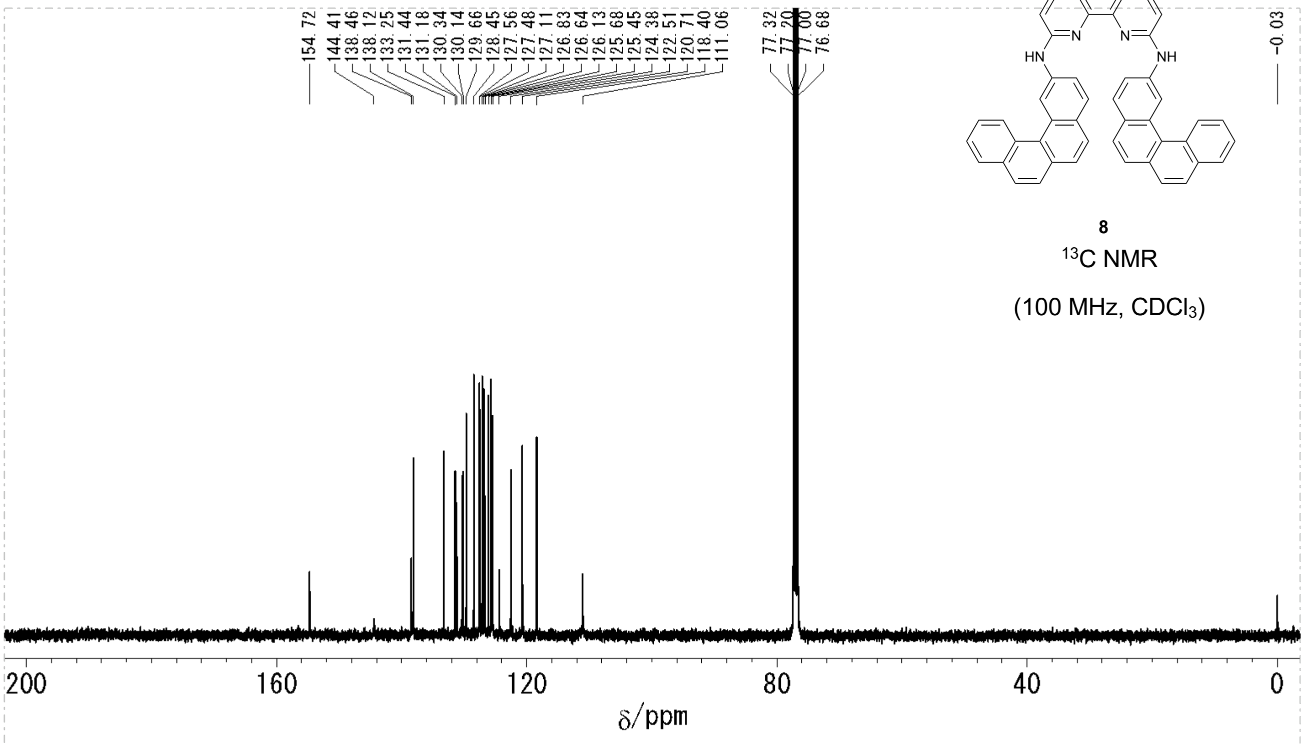
154.72 144.41 138.46 138.12 133.25 131.44 131.18 130.34 130.14 129.66 128.45 127.56 127.48 127.11 126.83 126.64 126.13 125.68 125.45 124.38 122.51 120.71 118.40 111.06
77.32 77.00 76.68
-0.03
HN
NH
N
N
8
^{13}C NMR
(100 MHz, $CDCl_3$)
δ/ppm
200 160 120 80 40 0

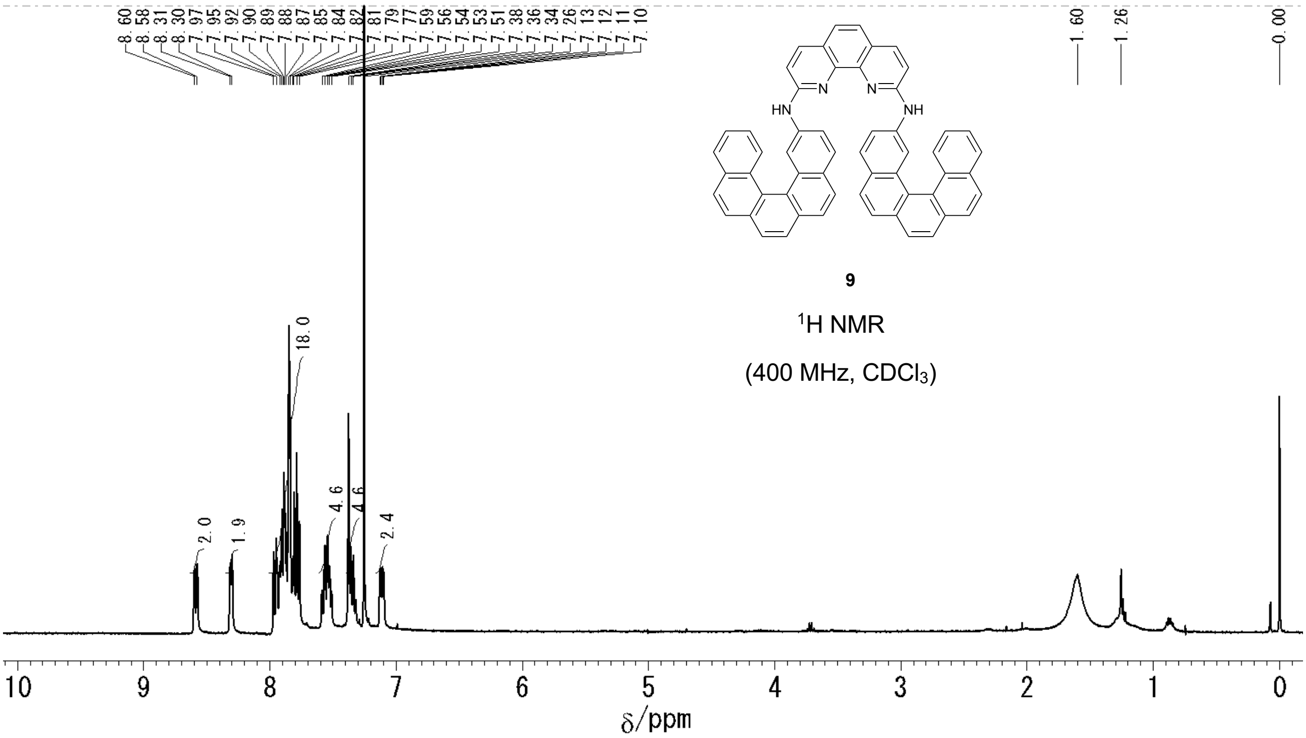
8.60
8.58
8.31
8.30
7.97
7.95
7.92
7.90
7.89
7.88
7.87
7.85
7.84
7.82
7.81
7.79
7.77
7.59
7.56
7.54
7.53
7.51
7.38
7.36
7.34
7.26
7.13
7.12
7.11
7.10
1.60
1.26
0.00
HN
NH
N
N
9
^{1}H NMR
(400 MHz, CDCl$_3$)
2.0
1.9
18.0
4.6
4.6
2.4
10
9
8
7
6
5
4
3
2
1
0
δ/ppm

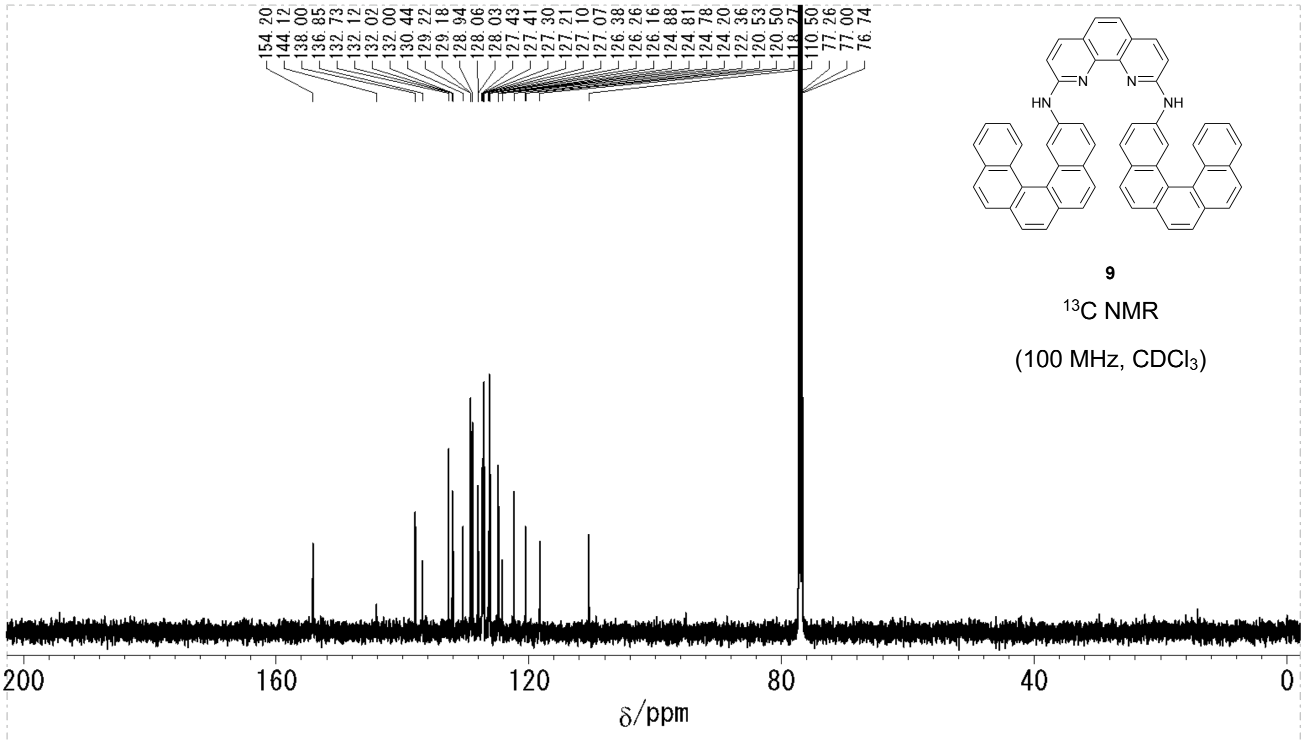
154.20
144.12
138.00
136.85
132.73
132.12
132.02
132.00
130.44
129.22
129.18
128.94
128.06
128.03
127.43
127.41
127.30
127.21
127.10
127.07
126.38
126.26
126.16
124.88
124.81
124.78
124.20
122.36
120.53
120.50
118.27
110.50
77.26
77.00
76.74
HN
NH
N
N
9
^{13}C NMR
(100 MHz, CDCl$_3$)
200
160
120
80
40
0
δ/ppm

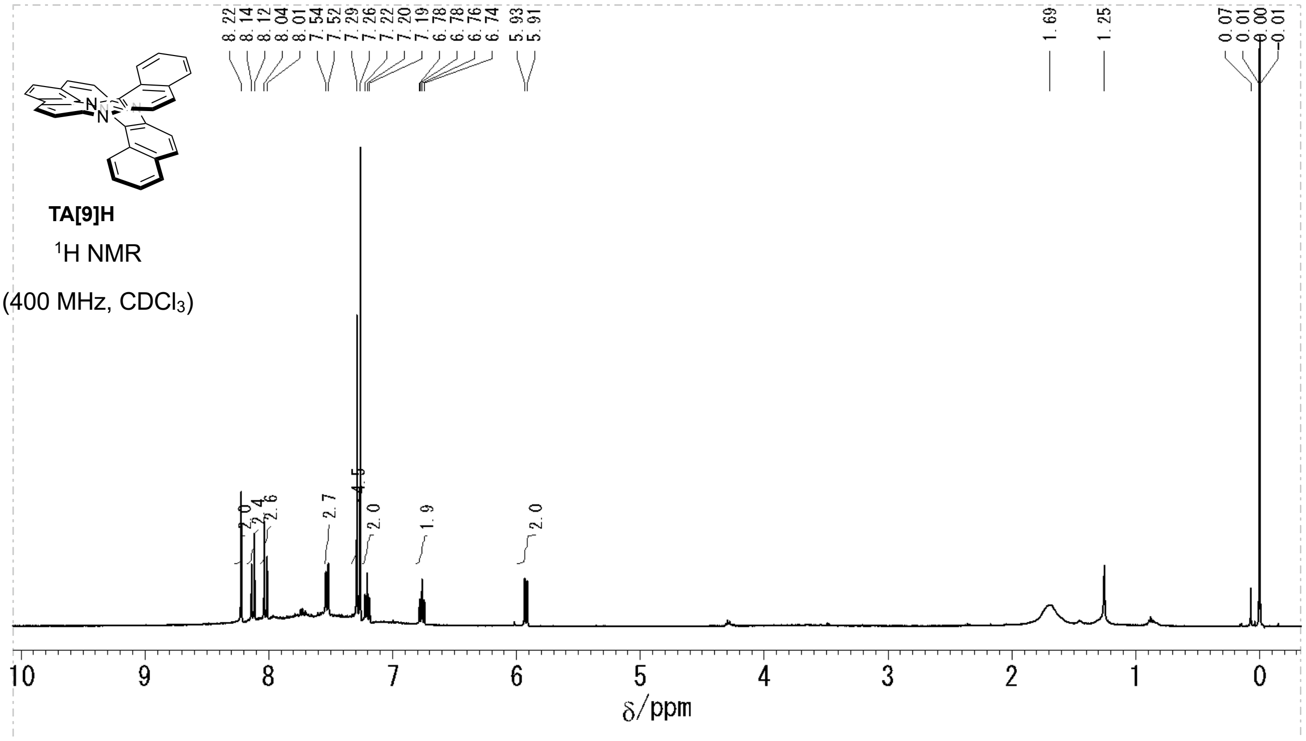
TA[9]H
^{1}H NMR
(400 MHz, $CDCl_3$)
8.22 8.14 8.12 8.04 8.01 7.54 7.52 7.29 7.26 7.22 7.20 7.19 6.78 6.78 6.76 6.74 5.93 5.91
1.69
1.25
0.07 0.01 0.00 -0.01
2.0 2.4 2.6 2.7 4.5 2.0 1.9 2.0
10 9 8 7 6 5 4 3 2 1 0
δ/ppm

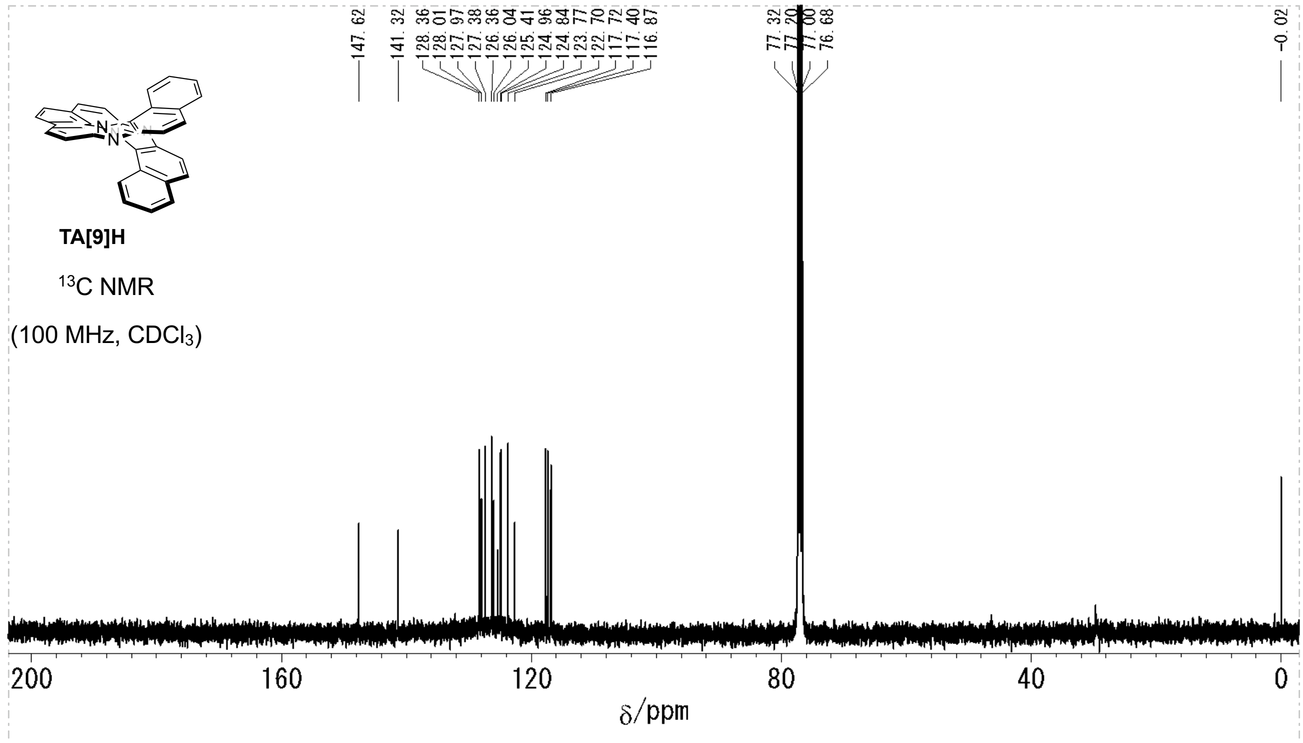
TA[9]H
^{13}C NMR
(100 MHz, $CDCl_3$)
147.62
141.32
128.36 128.01 127.97 127.38 126.36 126.04 125.41 124.96 124.84 123.77 122.70 117.72 117.40 116.87
77.32 77.20 77.00 76.68
-0.02
200 160 120 80 40 0
δ/ppm

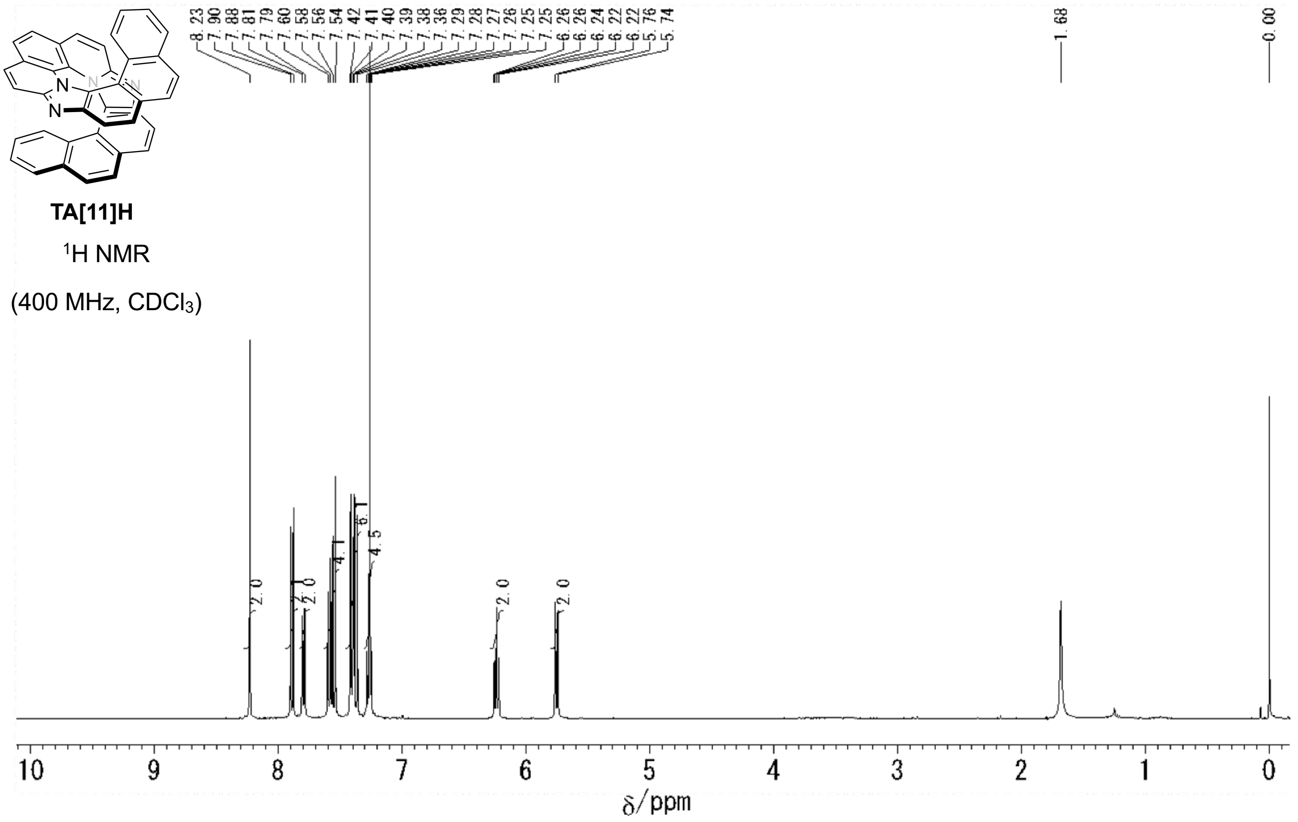

8.23 7.90 7.88 7.81 7.79 7.60 7.58 7.56 7.54 7.42 7.41 7.40 7.39 7.38 7.36 7.29 7.28 7.27 7.26 7.26 7.25 7.25 6.26 6.26 6.24 6.22 6.22 5.76 5.74
1.68
0.00
N
N
TA[11]H
1H NMR
(400 MHz, CDCl3)
2.0
2.0
4.1
6.1
4.5
2.0
2.0
10 9 8 7 6 5 4 3 2 1 0
δ/ppm

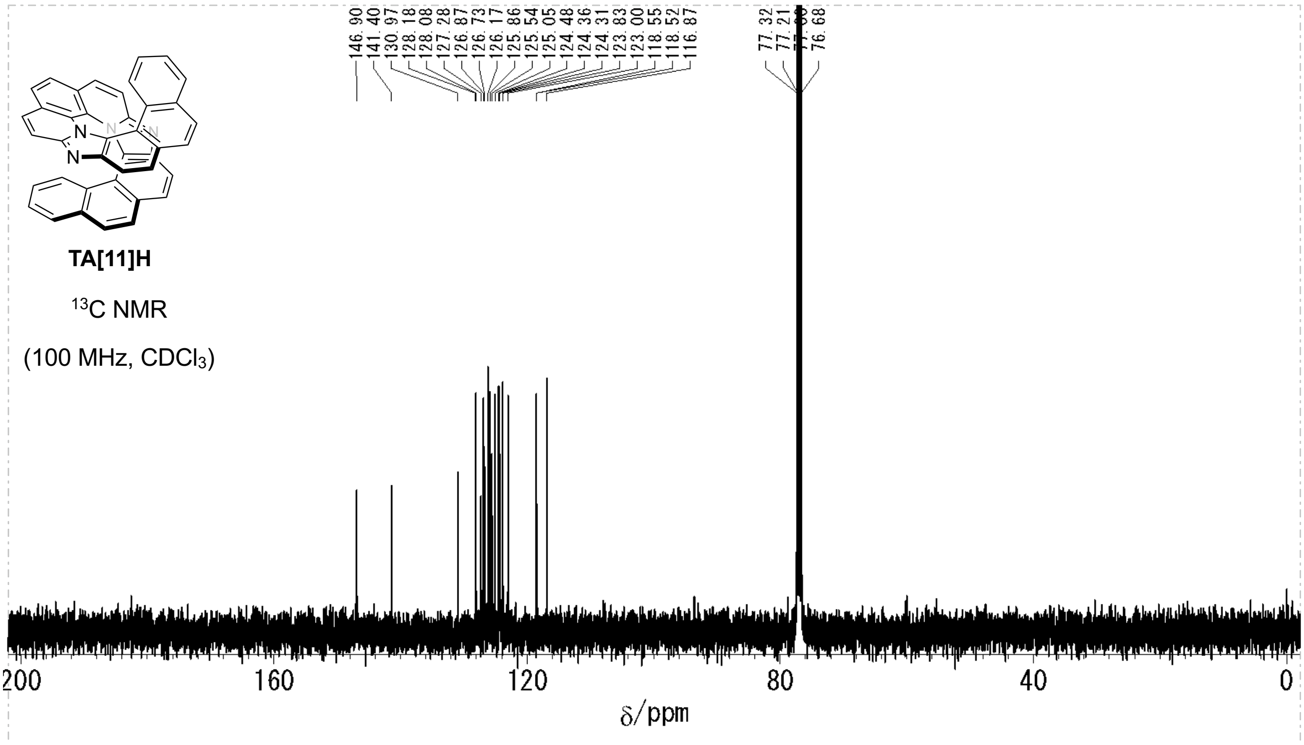

146.90 141.40 130.97 128.18 128.08 127.28 126.87 126.73 126.17 125.86 125.54 125.05 124.48 124.36 124.31 123.83 123.00 118.55 118.52 116.87
77.32 77.21 76.68
N
N
TA[11]H
13C NMR
(100 MHz, CDCl3)
200 160 120 80 40 0
δ/ppm

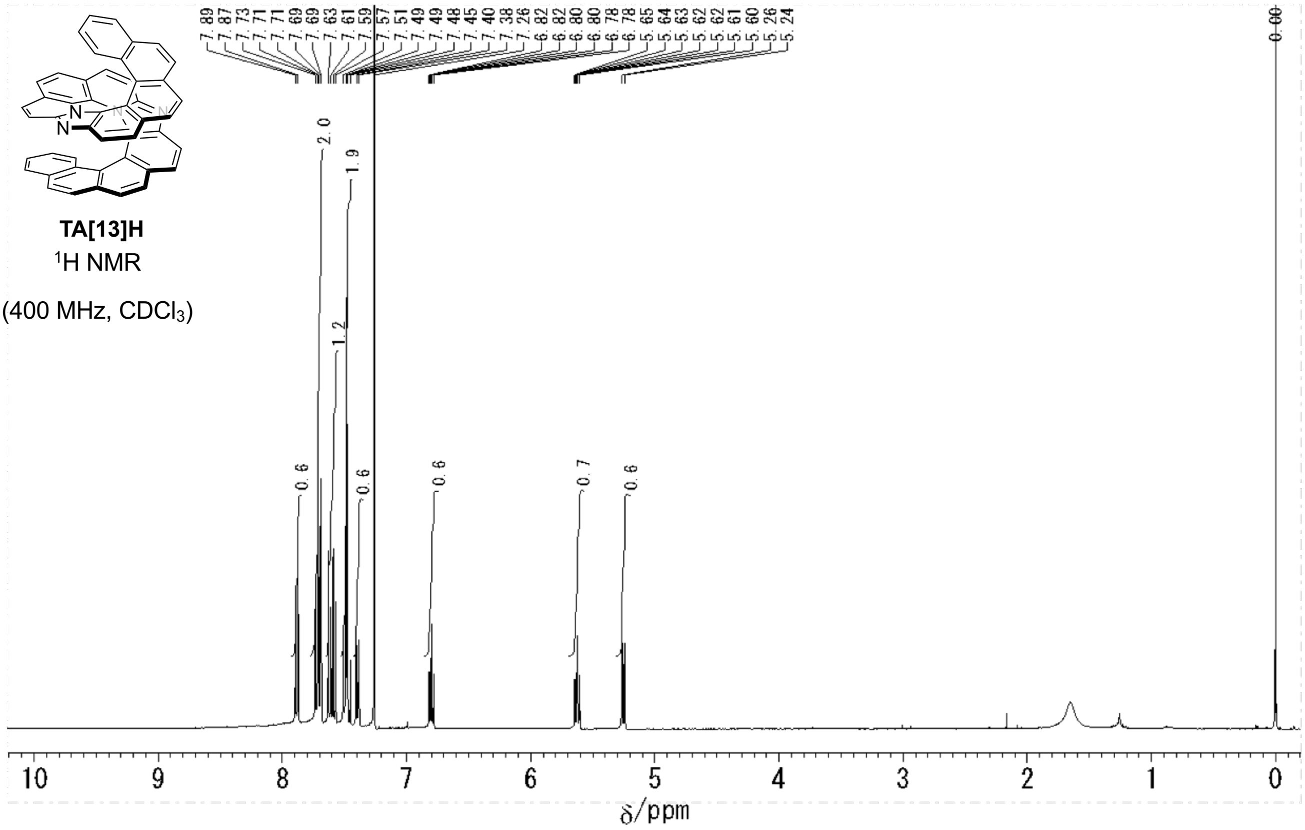
TA[13]H
1H NMR
(400 MHz, CDCl3)
7.89 7.87 7.73 7.71 7.71 7.69 7.69 7.63 7.61 7.59 7.57 7.51 7.49 7.48 7.45 7.40 7.38 7.26 6.82 6.82 6.80 6.80 6.78 6.78 5.65 5.64 5.63 5.62 5.62 5.61 5.60 5.26 5.24
0.00
2.0
1.9
1.2
0.6
0.6
0.6
0.7
0.6
10 9 8 7 6 5 4 3 2 1 0
δ/ppm

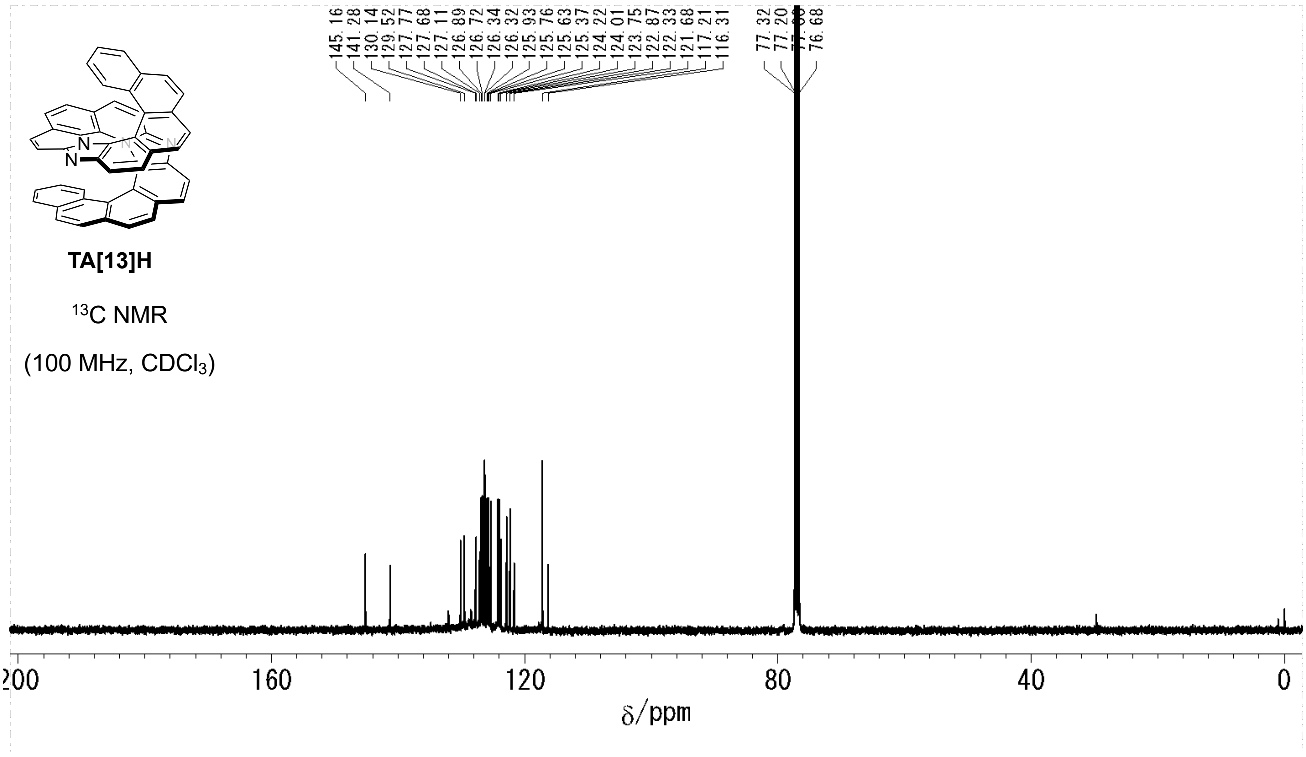
TA[13]H
13C NMR
(100 MHz, CDCl3)
145.16 141.28 130.14 129.52 127.77 127.68 127.11 126.89 126.72 126.34 126.32 125.93 125.76 125.63 125.37 124.22 124.01 123.75 122.87 122.33 121.68 117.21 116.31
77.32 77.20 76.68
200 160 120 80 40 0
δ/ppm

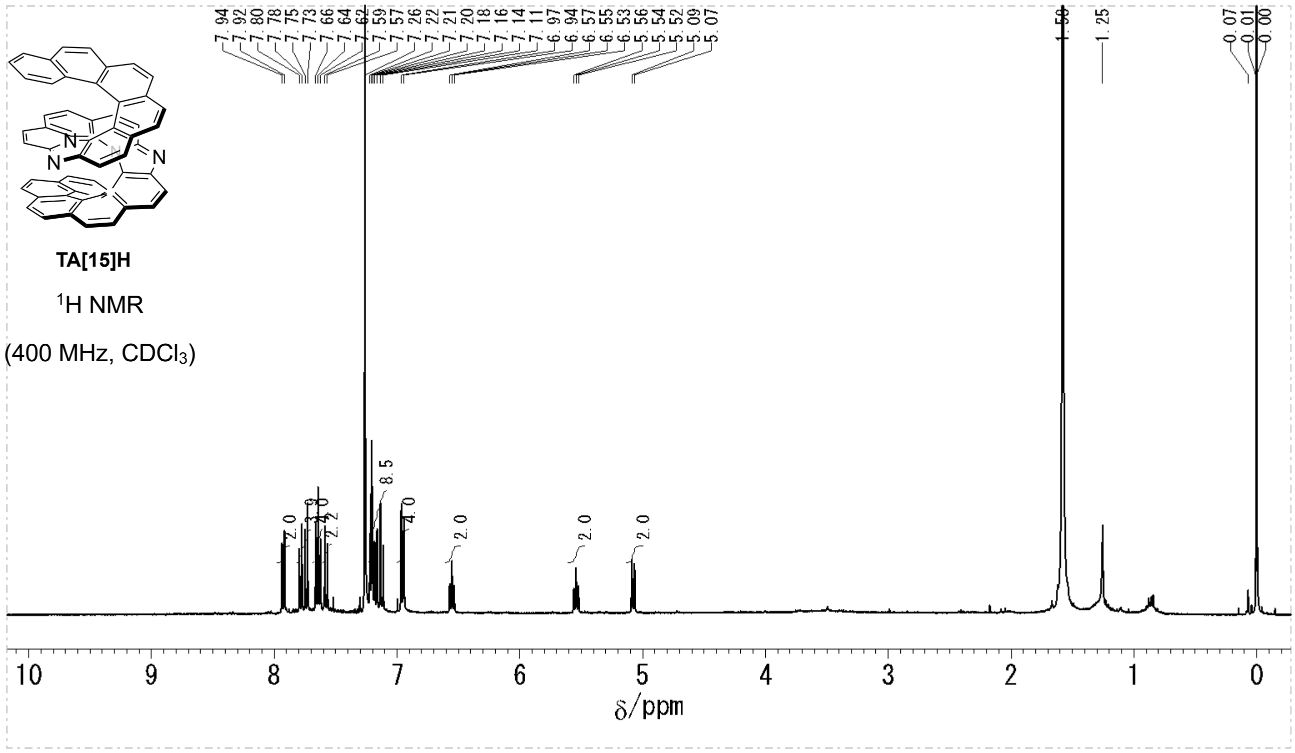
TA[15]H
^{1}H NMR
(400 MHz, CDCl$_3$)
δ/ppm

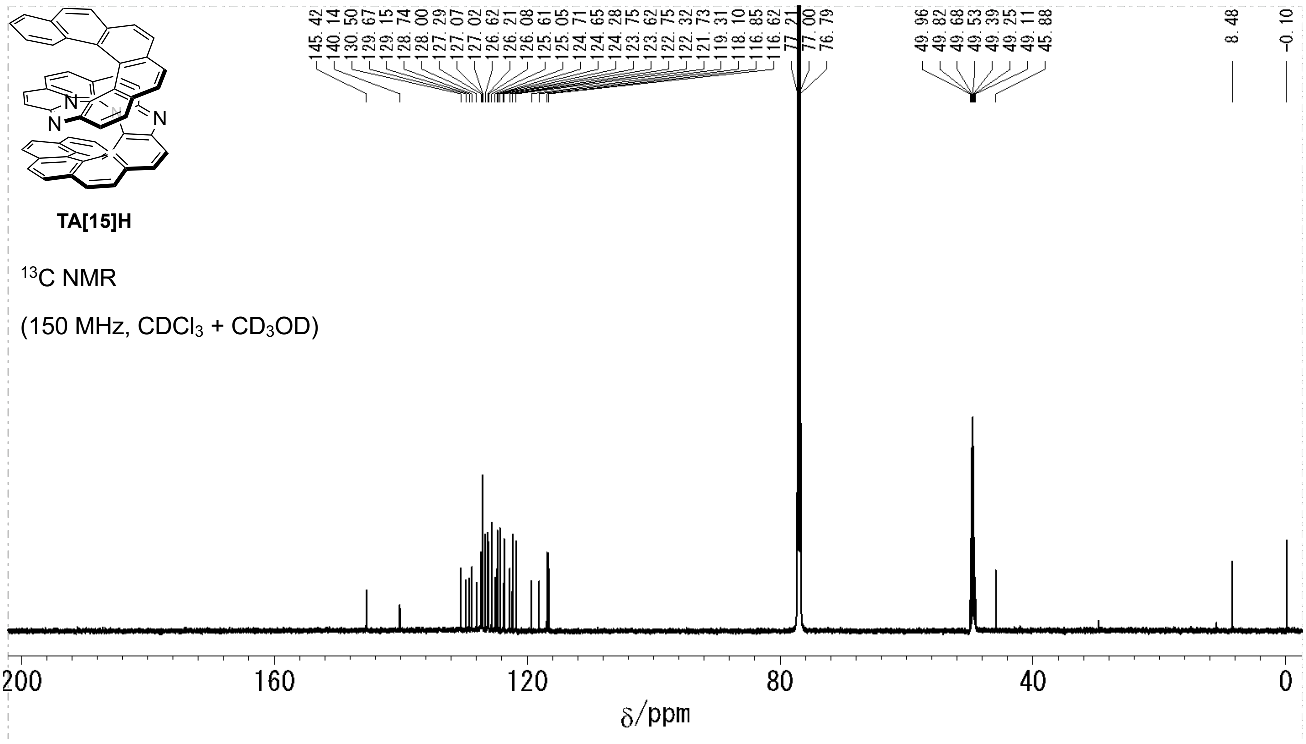
TA[15]H
^{13}C NMR
(150 MHz, CDCl$_3$ + CD$_3$OD)
δ/ppm
